# Biotransformations of Flavones and an Isoflavone (Daidzein) in Cultures of Entomopathogenic Filamentous Fungi

**DOI:** 10.3390/molecules23061356

**Published:** 2018-06-05

**Authors:** Monika Dymarska, Tomasz Janeczko, Edyta Kostrzewa-Susłow

**Affiliations:** Department of Chemistry, Faculty of Biotechnology and Food Science, Wrocław University of Environmental and Life Sciences, 50-375 Wrocław, Poland; janeczko13@interia.pl (T.J.); ekostrzew@gmail.com (E.K.-S.)

**Keywords:** biotransformations, flavone, daidzein, 5-hydroxyflavone, 6-hydroxyflavone, 7-hydroxyflavone, 7-aminoflavone, *Isaria fumosorosea*, *Isaria farinosa*

## Abstract

Entomopathogenic filamentous fungi of the genus *Isaria* are effective biocatalysts in the biotransformation of flavonoids as well as steroids. In the present study, the species *Isaria* *fumosorosea* and *Isaria* *farinosa* isolated from the environment were used. Their catalytic capacity to carry out biotransformations of flavones—unsubstituted, with hydroxy- and amino-substituents as well as a hydroxylated isoflavone—was investigated. Biotransformations of flavone, 5-hydroxyflavone, 6-hydroxyflavone, 7-hydroxyflavone, and daidzein resulted in the formation of *O*-methylglucosides, in the case of flavone and 5-hydroxyflavone with additional hydroxylations. 7-Aminoflavone was transformed into two acetamido derivatives. The following products were obtained: From flavone–flavone 2′-*O*-β-d-(4′′-*O*-methyl)-glucopyranoside, flavone 4′-*O*-β-d-(4′′-*O*-methyl)-glucopyranoside and 3′-hydroxyflavone 4′-*O*-β-d-(4′′-*O*-methyl)-glucopyranoside; from 5-hydroxyflavone–5-hydroxyflavone 4′-*O*-β-d-(4′′-*O*-methyl)-glucopyranoside; from 6-hydroxyflavone–flavone 6-*O*-β-d-(4′′-*O*-methyl)-glucopyranoside; from 7-hydroxyflavone–flavone 7-*O*-β-d-(4′′-*O*-methyl)-glucopyranoside; from daidzein–daidzein 7-*O*-β-d-(4′′-*O*-methyl)-glucopyranoside; and from 7-aminoflavone–7-acetamidoflavone and 7-acetamido-4′-hydroxyflavone. Seven of the products obtained by us have not been previously reported in the literature.

## 1. Introduction

Flavonoid compounds are used in pharmacy [1] and the food industry [2], and their effectiveness in the prevention and treatment of a number of diseases, including civilization diseases, is confirmed by many studies both in vitro on cell lines and in vivo [3,4,5,6,7,8]. However, it is not certain whether the substance taken orally reaches its destination in an unchanged form, whether it undergoes biotransformation in the body and whether the acting therapeutic compound is its metabolite [8,9].

The limitation in the use of many flavonoid compounds with high biological activity proved in vitro is their low bioavailability resulting inter alia from poor solubility in water [7,10,11]. Flavonoid compounds in the small intestine are absorbed only to a small extent (5–10%) [9]. The majority of flavonoid compounds that are taken along with the diet pass to the large intestine, where they undergo intense bacterial metabolism [9,12]. The effect of these transformations is often the creation of products with higher activity than the starting compound. This is the case of soy isoflavones, which can be metabolized to equol—a compound with significantly higher estrogenic activity [13].

Flavonoids in plants are mainly in the form of glycosides. Thermal processing of food does not damage glycosidic bonds, and therefore flavonoid glycosides predominate in products of plant origin taken with the diet. The issue of absorption of sugar glycoside combinations remains unexplained. There are reports of possible absorption of flavonoid glycosides in the small intestine. However, many studies have shown that these compounds reach the large intestine in unchanged form and only after glycosidic bonds are broken by the intestinal microflora can they be absorbed [14,15,16,17,18].

The preparation of flavonoid glycosides by chemical synthesis is often impossible due to the drastic conditions leading to the degradation of substrates, the yields of the process are low, and undesirable anomers are formed [19]. Biotransformations are considered to be an environmentally friendly alternative that allows modification of the structure of compounds to improve their properties [20]. The number of literature reports on the microbiological production of flavonoid sugar derivatives is on the rise. The use of microorganisms makes it possible to obtain compounds with high regio- and stereoselectivity. Many flavonoid glycosides that are naturally present in plants in small amounts and are difficult to isolate along with novel compounds can already be effectively obtained from flavonoid aglycones by using microorganisms as biocatalysts [21,22,23,24,25].

*Isaria fumosorosea* is an entomopathogenic filamentous fungus with a wide range of possible applications. Microorganisms of the species *I. fumosorosea* are used in agriculture as a natural alternative to chemical pesticides [26,27]. Fumosorinone synthesized by *I. fumosorosea* has an anti-diabetic effect [28]. In addition, research carried out by our team has shown that the strain of this fungus, isolated from a spider from urban green areas in Wroclaw, is an excellent biocatalyst in the biotransformations of flavonoid compounds and steroids by carrying out reactions not known in the literature [1,29]. For the first time, we described the transformation in which the sugar unit is attached to the flavonoid aglycone lacking hydroxyl groups in its structure [1]. Additionally, we had 13 strains of *Isaria farinosa* fungi isolated from Lower Silesia adits. So far, we have described catalytic abilities in steroid biotransformations of several strains from the *I. farinosa* species [30]. Initial studies indicate that these strains were also efficient biocatalysts in flavonoid biotransformations. 

The main aim of this study was to evaluate the catalytic capacity of *Isaria fumosorosea* KCH J2, *I. farinosa* J1.4, *I. farinosa* J1.6 and *I. farinosa* KW1.2 to carry out biotransformations of flavone, 5-hydroxyflavone, 6-hydroxyflavone, 7-hydroxyflavone, daidzein, and 7-aminoflavone. For all substrates used, flavonoid *O*-methylglucosides were formed, but not for 7-aminoflavone which resulted in formation of acetamido derivatives.

## 2. Results and Discussion

The present results are a continuation of research into the microbiological transformations of flavonoid compounds in cultures of entomopathogenic filamentous fungi. In our previous publication, we presented a biotransformation of 6-methylflavone in *I. fumosorosea* KCH J2 culture. As a result of 7-day biotransformation, we obtained two new products: 6-methylflavone 8-*O*-β-d-(4′′-*O*-methyl)-glucopyranoside (6.6% yield) and 6-methylflavone 4′-*O*-β-d-(4′′-*O*-methyl)-glucopyranoside (13.2% yield) [1].

In the present paper, we used six flavonoid substrates and four strains of entomopathogenic filamentous fungi that were isolated from the environment. Detailed characteristics of the strains we used can be found in our previous publications [1,30].

In screening, the only strain that carried out biotransformations of all selected substrates was *I. fumosorosea* KCH J2. The yields obtained as a result of the use of this strain were the highest; therefore, it was chosen as a biocatalyst for scaled-up biotransformations. Experiments conducted on a larger scale enabled us to determine the chemical structures of products and their isolated yields. In the course of our study, we obtained nine biotransformation products, seven of which (flavone 2′-*O*-β-d-(4′′-*O*-methyl)-glucopyranoside (**1a**), flavone 4′-*O*-β-d-(4′′-*O*-methyl)-glucopyranoside (**1b**), 3′-hydroxyflavone 4′-*O*-β-d-(4′′-*O*-methyl)-glucopyranoside (**1c**), 5-hydroxyflavone 4′-*O*-β-d-(4′′-*O*-methyl)-glucopyranoside (**2a**), flavone 6-*O*-β-d-(4′′-*O*-methyl)-glucopyranoside (**3a**), flavone 7-*O*-β-d-(4′′-*O*-methyl)-glucopyranoside (**4a**), and 4′-hydroxy-7-acetamidoflavone (**6b**) have not been previously described in the literature.

### 2.1. Biotransformations of Flavone *(**1**)*

**Scheme 1:**
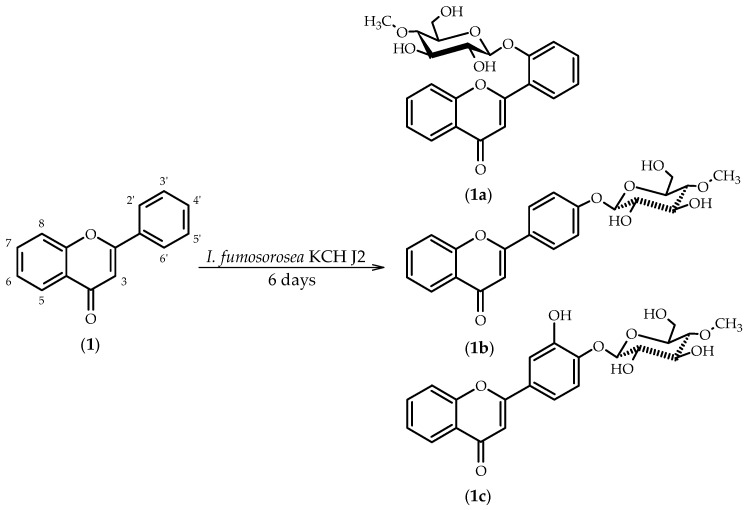
Microbial transformation of flavone (**1**) in *I. fumosorosea* KCH J2 culture.

As a result of 6-day biotransformation of flavone (**1**) in the culture of *I. fumosorosea* KCH J2 we obtained three products: Flavone 2′-*O*-β-d-(4′′-*O*-methyl)-glucopyranoside (**1a**) with 5.5% yield, flavone 4′-*O*-β-d-(4′′-*O*-methyl)-glucopyranoside (**1b**) with 14% yield, and 3′-hydroxyflavone 4′-*O*-β-d-(4′′-*O*-methyl)-glucopyranoside (**1c**) with 4% yield (Scheme 1). Compound **1b** was also formed when we used *I. farinosa* J1.6 as a biocatalyst. 

The presence of a glucose unit in molecules **1a**, **1b**, and **1c** was confirmed by five characteristic carbon signals observed in the region from about δ = 80.0 ppm to about δ = 62.0 ppm in the ^13^C-NMR spectra, along with proton signals of δH ranging from about δ = 3.90 ppm to δ = 3.29 ppm in the ^1^H-NMR spectra. Additionally, the attachment of a sugar unit to substrate **1** was confirmed by a one-proton doublet visible at δ = 5.23 ppm in the ^1^H-NMR spectrum of **1a**, δ = 5.14 ppm in the ^1^H-NMR spectrum of **1b** and δ = 5.00 ppm in the ^1^H-NMR spectrum of **1c**, which are due to protons at the hemiacetal carbon atoms. The β-configuration of the glucose unit was proved by the coupling constant (*J* = 7.6 Hz/*J* = 7.7 Hz/*J* = 7.8 Hz) for the anomeric proton. A three-proton singlet at about δ = 3.60 ppm in the ^1^H-NMR and the corresponding signal at about δ = 60.6 ppm in the ^13^C-NMR proved that one of the hydroxyl groups had been methylated. *O*-methylation occurred in the C-4 hydroxyl group of the glucose moieties, which was confirmed in the correlation spectra, where the proton signals due to -OCH_3_ were coupled with the signal of C-4 (about δ = 80.0 ppm) in the glucose unit. In the case of compound **1a**, the sugar unit was attached to C-2′, because in the correlation spectrum the signal due to the proton at the hemiacetal carbon atom (δ = 5.23 ppm) was coupled with the C-2′ signal (δ = 156.7 ppm), which was shifted from δ = 127.2 ppm, indicating the attachment of an electronegative atom. In addition, protons at C-3′ and C-5′ become non-equivalent, two triplets can be observed from protons at C-4′ (δ = 7.57 ppm) and C-5′ (δ = 7.27 ppm) and two doublets from protons at C-3′ (δ = 7.44 ppm) and C-6′ (δ = 7.99 ppm). In the case of **1b**, the sugar unit was attached to C-4′, because in the correlation spectrum the signal due to the proton at the hemiacetal carbon atom (δ = 5.14 ppm) was coupled with the C-4′ signal (δ = 161.4 ppm), which was shifted from δ = 132.5 ppm. The substitution in position 4′ was also evidenced by the characteristic two doublets in the ^1^H-NMR spectrum from protons at C-2′ and C-6′ (δ = 8.09 ppm) as well as C-3′ and C-5′ (δ = 7.28 ppm). In the case of **1c**, the sugar unit was attached to C-4′, because in the correlation spectrum the signal due to the proton at the hemiacetal carbon atom (δ = 5.14 ppm) was coupled with the C-4′ signal (δ = 148.9 ppm), which was shifted from δ = 132.5 ppm. The substitution at the carbon 3′ was evidenced by the ^1^H-NMR spectrum of **1c**: The signal from the proton at C-2′ was visible as a singlet and there is no signal from the proton at C-3′. Hydroxylation was assumed because of the shift of the C-3′ carbon signal from δ = 129.0 to δ = 148.7. Chemical shifts of the other signals in the ^1^H and ^13^C-NMR spectra have only slightly changed, which indicates that the flavone skeleton remained intact.

### 2.2. Biotransformations of 5-Hydroxyflavone *(**2**)*

**Scheme 2:**
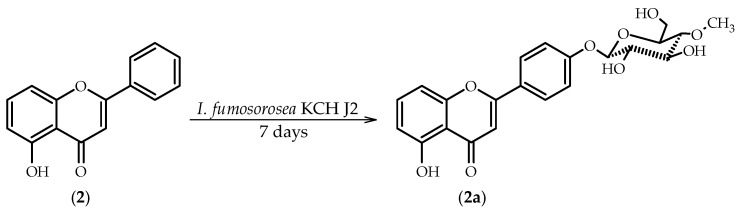
Microbial transformation of 5-hydroxyflavone (**2**) in *I. fumosorosea* KCH J2 culture.

As a result of 7-day biotransformation of 5-hydroxyflavone (**2**) in the culture of *I. fumosorosea* KCH J2, we obtained 5-hydroxyflavone 4′-*O*-β-d-(4′′-*O*-methyl)-glucopyranoside (**2a**) with 29% yield (Scheme 2). Compound **2a** was also formed when we used *I. farinosa* J1.4 as a biocatalyst. 

The presence of a glucose unit in molecule **2a** was confirmed by five characteristic carbon signals observed in the region from δ = 80.1 ppm to δ = 62.0 ppm in the ^13^C-NMR spectra, along with proton signals of δH ranging from about δ = 3.89 ppm to δ = 3.27 ppm in the ^1^H-NMR spectra. The attachment of a sugar unit to substrate **2** was also confirmed by a one-proton doublet visible at δ = 5.15 ppm in the ^1^H-NMR spectrum of **2a**, which was due to protons at the hemiacetal carbon atoms. The β-configuration of the glucose unit was proved by the coupling constant (*J* = 7.7 Hz) for the anomeric proton. A three-proton singlet at δ = 3.61 ppm in the ^1^H-NMR and the corresponding signal at δ = 60.6 ppm in the ^13^C-NMR indicates the *O*-methylation of the hydroxyl group at C-4 of glucose, which was confirmed on the basis of the analysis of the correlation spectra of compound **2a**, where the proton signals due to -OCH_3_ (δ = 3.61 ppm) were coupled with the signal of C-4 (δ = 80.1 ppm) in the glucose unit. The sugar unit was attached to C-4′, because in the correlation spectrum the signal due to the proton at the hemiacetal carbon atom (δ = 5.15 ppm) was coupled with the C-4′ signal (δ = 161.8 ppm). There was no signal from the proton at C-4′ in the ^1^H-NMR spectrum. Two doublets from the protons at C-2′ and C-6′ (δ = 8.10 ppm) and C-3′ and C-5′ (δ = 7.29 ppm) can be observed. 

### 2.3. Biotransformations of 6-Hydroxyflavone *(**3**)*

**Scheme 3:**
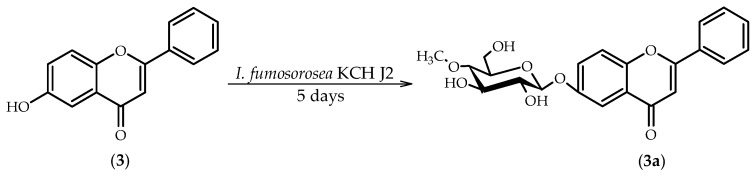
Microbial transformation of 6-hydroxyflavone (**3**) in *I. fumosorosea* KCH J2 culture.

As a result of 5-day biotransformation of 6-hydroxyflavone (**3**) in the culture of *I. fumosorosea* KCH J2, we obtained 6-*O*-β-d-(4′′-*O*-methyl)-glucopyranoside (**3a**) with 13% yield (Scheme 3). Product **3a** was also formed when we used *I. farinosa* J1.6 and *I. farinosa* KW1.2 as biocatalysts. 

The presence of a glucose unit in molecule **3a** was confirmed by five characteristic carbon signals observed in the region from δ = 79.9 ppm to δ = 61.9 ppm in the ^13^C-NMR spectra and proton signals from about δ = 3.89 ppm to δ = 3.31 ppm in the ^1^H-NMR spectra. Additionally, the attachment of a sugar unit to substrate **3** was confirmed by a one-proton doublet at δ = 5.11 ppm in the ^1^H-NMR spectrum of **3a**, which was due to protons at the hemiacetal carbon atoms. The β-configuration of the glucose unit was proved by the coupling constant (*J* = 7.8 Hz) for the anomeric proton. A three-proton singlet at δ = 3.62 ppm in the ^1^H-NMR and the corresponding signal at δ = 60.6 ppm in the ^13^C-NMR prove that one of the hydroxyl groups was methylated. *O*-methylation occurred in the C-4 hydroxyl group of the glucose moiety, which was confirmed in the correlation spectra of compound **3a**, where the proton signals due to -OCH_3_ (δ = 3.62 ppm) were coupled with the signal of C-4 (δ = 79.9 ppm) in the glucose unit. The sugar unit was attached to C-6, because in the correlation spectrum the signal due to the proton at the hemiacetal carbon atom (δ = 5.11 ppm) was coupled with the C-6 signal (δ = 156.0 ppm). The signals in the ^1^H spectrum from the protons at C-5, C-7 and C-8 were shifted towards lower field values, indicating the appearance of a deactivating (electron-withdrawing) substituent in the A ring of flavonoid. 

### 2.4. Biotransformations of 7-Hydroxyflavone *(**4**)*

**Scheme 4:**
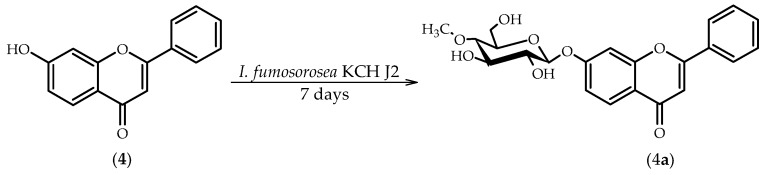
Microbial transformation of 7-hydroxyflavone (**4**) in *I. fumosorosea* KCH J2 culture.

As a result of 7-day biotransformation of 7-hydroxyflavone (**4**) in the culture of *I. fumosorosea* KCH J2, we obtained flavone 7-*O*-β-d-(4′′-*O*-methyl)-glucopyranoside (**4a**) with 16% yield (Scheme 4). Product **4a** was also formed when we used *I. farinosa* J1.4 and *I. farinosa* J1.6 as biocatalysts. 

The presence of a glucose unit in molecule **4a** was confirmed by five characteristic carbon signals observed in the region from δ = 80.0 ppm to δ = 61.9 ppm in the ^13^C-NMR spectra, along with proton signals of δH ranging from about δ = 3.92 ppm to δ = 3.30 ppm in the ^1^H-NMR spectra. Additionally, the attachment of a sugar unit to substrate **4** was confirmed by a one-proton doublet at δ = 5.24 ppm in the ^1^H-NMR spectrum of **4a** from protons at the hemiacetal carbon atoms. The β-configuration of the glucose unit was proved by the coupling constant (*J* = 7.7 Hz) for the anomeric proton. A three-proton singlet at δ = 3.62 ppm in the ^1^H-NMR and the corresponding signal at δ = 60.6 ppm in the ^13^C-NMR proved that one of the hydroxyl groups were methylated. *O*-methylation occurred in the C-4 hydroxyl group of the glucose moiety, which was confirmed in the correlation spectra of compound **4a**, where the proton signals due to -OCH_3_ (δ = 3.62 ppm) were coupled with the signal of C-4 (δ = 80.0 ppm) in the glucose unit. The attachment of a sugar unit to C-7 was proved in the correlation spectrum. The signal due to the proton at the hemiacetal carbon atom (δ = 5.24 ppm) was coupled with the C-7 signal (δ = 162.9 ppm). The signals in the ^1^H spectrum from the protons at C-6 and C-8 were shifted towards lower field values, suggesting the appearance of a strongly electronegative substitute in their vicinity.

### 2.5. Biotransformations of Daidzein *(**5**)*

**Scheme 5:**
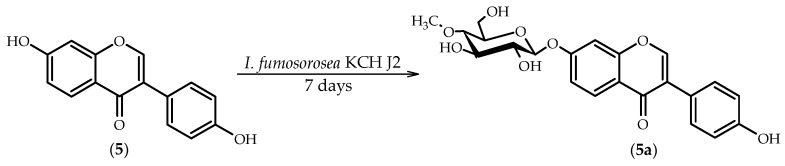
Microbial transformation of daidzein (**5**) in *I. fumosorosea* KCH J2 culture.

As a result of 7-day biotransformation of daidzein (**5**) in the culture of *I. fumosorosea* KCH J2 we obtained 4′-hydroxyisoflavone 7-*O*-β-d-(4′′-*O*-methyl)-glucopyranoside (4′′-*O*-methyldaidzin) (**5a**) with 14.8% yield (Scheme 5). Product **5a** was also formed when we used *I. farinosa* J1.6 and *I. farinosa* KW1.2 as biocatalysts.

The presence of a glucose unit in molecule **5a** was confirmed by five characteristic carbon signals observed in the region from δ = 80.0 ppm to δ = 62.0 ppm in the ^13^C-NMR spectra, along with proton signals of δH ranging from about δ = 3.92 ppm to δ = 3.28 ppm in the ^1^H-NMR spectra. Additionally, the attachment of a sugar unit to substrate **5** was confirmed by a one-proton doublet visible at δ = 5.21 ppm in the ^1^H-NMR spectrum of **5a** that came from protons at the hemiacetal carbon atoms. The β-configuration of the glucose unit was proved by the coupling constant (*J* = 7.7 Hz) for the anomeric proton. A three-proton singlet at δ = 3.62 ppm in the ^1^H-NMR and the corresponding signal at δ = 60.6 ppm in the ^13^C-NMR proved that one of the hydroxyl groups had been methylated. *O*-methylation occurred in the C-4 hydroxyl group of the glucose moiety, which was confirmed in the correlation spectra of compound 5a, where the proton signals coming from-OCH_3_ (δ = 3.62 ppm) were coupled with the signal of C-4 (δ = 80.0 ppm) in the glucose molecule. The sugar unit was attached to C-7, because in the correlation spectrum, the signal due to the proton at the hemiacetal carbon atom (δ = 5.21 ppm) was coupled with the C-7 signal (δ = 162.6 ppm). 

### 2.6. Biotransformations of 7-Aminoflavone *(**6**)*

**Scheme 6:**
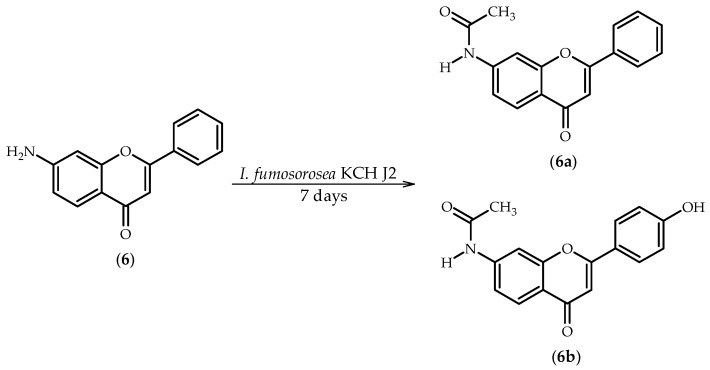
Microbial transformation of 7-aminoflavone (**6**) in *I. fumosorosea* KCH J2 culture.

As a result of 7-day biotransformation of 7-aminoflavone (**6**) in the culture of *I. fumosorosea* KCH J2 we obtained two products: 7-acetamidoflavone (**6a**) with 10% yield and 4′-hydroxy-7-acetamidoflavone (**6b**) with 3% yield (Scheme 6). 

In the case of compound **6a** (**6b**), a broad singlet at δ = 9.78 ppm (δ = 9.73) in the ^1^H-NMR corresponds to the -NH proton attached to C-7 and was not present in the spectrum of the substrate (**6**). For compound **6**, the signal of the two amino protons was observed at δ = 5.82 ppm in the ^1^H-NMR spectrum. In the HMBC spectrum, a -CH_3_ signal, absent in the case of the substrate (singlet at δ = 2.21 ppm on ^1^H-NMR and δ = 24.5 ppm (δ = 24.5 ppm for **6b**) on ^13^C-NMR) correlated with the signal at δ = 169.9 ppm (δ = 169.8 ppm for **6b**) from the carbonyl carbon of the acetamide group. Carbonyl carbon from the acetamide group correlated with the signal at δ = 7.46 ppm that came from the proton at C-6. The signals in the ^1^H spectrum from the protons at C-5, C-6 and C-8 were shifted towards lower field values, suggesting the appearance of a deactivating substituent in their vicinity. In addition, the ^1^H-NMR spectrum of compound **6b** showed a characteristic pattern of signals indicating substitution at position 4′: Two two-proton signals at δ = 8.00 and δ = 7.08, from protons at C-3′ and C-5′ and at C-2′ and C-6′, the signal from the proton at C-4′ was also no longer visible. The C-4′ carbon signal was shifted from δ = 131.9 to δ = 161.6 in the ^13^C-NMR spectrum, which indicates the attachment of a strongly electronegative atom. A broad singlet at δ = 9.27 ppm from the proton of the hydroxyl group can be observed in the ^1^H-NMR spectrum. Chemical shifts of the other signals in the ^1^H and ^13^C-NMR spectra have only slightly changed, which indicates that the flavone skeleton remained intact.

The *I. fumosorosea* KCH J2 strain was characterized by low substrate specificity; it was capable of catalyzing biotransformations of flavones with hydroxyl, methyl, and amino substituents as well as unsubstituted flavone and hydroxylated isoflavone. Biotransformations of flavone confirmed our earlier observations: the sugar derivative of the flavonoid compound can be formed despite the lack of a hydroxyl group in its structure [1]. *I. fumosorosea* KCH J2 was to date the only strain found to possess this unique capability. Our present study proves that *I. farinosa* J1.6 is also capable of performing such bioconversion. 

In the case of hydroxy derivatives of flavonoids, attachment of the sugar unit to the hydroxyl group takes place, but not for 5-hydroxyflavone. This may be due to the proximity of the carbonyl oxygen, which is a steric hindrance and prevents the flavonoid substrate fitting into a pocket in the enzyme’s active site so that the compound can be functionalized in position 5. It may also be due to the formation of hydrogen bonds between the hydroxyl group present in the 5-position and the oxygen from the carbonyl group of the substrate. In the available literature, there are no reports on microbial glycosylation in the 5-position of popular flavones, such as apigenin (5,7,4′-trihydroxyflavone), chrysin (5,7-dihydroxyflavone), baicalein (5,6,7-trihydroxyflavone), or wogonin (5,7-dihydroxy-8-methoxyflavone). In the case of quercetin (3,5,7,3′,4′-pentahydroxyflavone) in the culture of *Cunninghamella elegans* ATCC 9245 the glucose unit was attached in the 3 position [31], the same as when *Gliocladium deliquescens* NRRL 1086 was used as a biocatalyst in biotransformations of quercetin, kaempferol (3,5,7,4′-tetrahydroxyflavone), morin (3,5,7,2′,4′-pentahydroxyflavone), galangin (3,5,7-trihydroxyflavone) and myricetin (3,5,7,3′,4′,5′-hexahydroxyflavone) [23]. Usually when a microbiological catalyst is used, the sugar unit is attached to the flavonoid aglycone in position 3 or 7 [23,25]. A rare example of attaching glucose in the 5 position of flavonoid aglycone was biotransformation of naringenin (5,7,4′-trihydroxyflavanone) in the plant culture of *Eucalyptus perriniana*. The research reports the formation of several glycosides, e.g., when the sugar is attached to hydroxyl groups in positions 5 and 7 [32]. Flavones, unlike flavanones, have a double bond between C2 and C3. The presence of this bond provides the molecule with planarity and is of great importance for interaction with enzymes and the biological activity exhibited by the compounds [7,33].

4′′-*O*-methyldaidzin (**5a**) was obtained earlier as a result of biotransformation of daidzein in the culture of *Beauveria bassiana* AM 278 [34] as well as from a soybean broth fermented by *Paecilomyces militaris* [35]. Both strains used as biocatalysts belong to entomopathogenic filamentous fungi.

7-Aminoflavone was the only substrate for which we did not observe the formation of a glucoside derivative. The only example of biotransformation of 7-aminoflavone available in the literature to date is the transformation of the substrate into 7-hydroxyflavone in the filamentous fungi cultures of *Aspergillus niger* SBJ and *Aspergillus oryzae* 13/5 described by us [36]. The acetamido derivative of aminoflavone was obtained through the microbiological transformation of 6-aminoflavone in the cultures of *Rhodococcus* sp. DSM 364 and *Gordonia* sp. DSM 44456 [37]. The method for the preparation of 7-acetamidoflavone is known by chemical synthesis from 4-acetamido-2-hydroxydibenzoylmethane. The substrate dissolved in cold concentrated sulfuric acid was left for two hours. The product obtained after the addition of ice crystallized from the alcohol in the form of white needles [38].

Flavonoid glycosides are usually far more expensive than their parent aglycones [39]. This is due to the lack of economically viable methods for obtaining those derivatives via chemical or enzymatic synthesis, which we have discussed more broadly in our previous paper [1]. In this study we present a cheap, efficient, and environmentally friendly method for obtaining flavonoid methylglucosides from various flavonoid substrates. The next goal we set for ourselves is to attempt to increase the efficiency of the biotransformations described by us. We plan to check how the earlier addition of substrate to the culture of the microorganism will change the amount of products being formed, as was the case with the cultures of the genera *Aspergillus* and *Penicillium* [20,40]. We will also determine the optimal temperature and composition of the culture medium for the process, in order to conduct large-scale cultures in the bioreactor at the final stage. The next step will be an attempt to thoroughly examine the enzymes involved in the biotransformations described by us. 

## 3. Materials and Methods 

### 3.1. Chemicals

The substrates for biotransformations except for daidzein were purchased from Sigma Chemical Company (St. Louis, MO, USA). 

Daidzein was obtained via column chromatography of soybean (*Glycine max*) extract. Column chromatography (SiO_2_, Kieselgel 60, 230–400 mesh, 40–63 µm, Merck, Darmstadt, Germany) was performed using ethyl acetate: methylene chloride (1:1) mixture as an eluent.

#### 3.1.1. Flavone (**1**)

C_15_H_10_O_2_, *t*_R_ 18.99; ^1^H-NMR, see Table 1; ^13^C-NMR, see Table 2.

#### 3.1.2. 5-Hydroxyflavone (**2**)

C_15_H_10_O_3_, *t*_R_ 22.98; ^1^H-NMR, see Table 3; ^13^C-NMR, see Table 4.

#### 3.1.3. 6-Hydroxyflavone (**3**)

C_15_H_10_O_3_, *t*_R_ 15.25; ^1^H-NMR, see Table 3; ^13^C-NMR, see Table 4.

#### 3.1.4. 7-Hydroxyflavone (**4**)

C_15_H_10_O_3_, *t*_R_ 14.33; ^1^H-NMR, see Table 3; ^13^C-NMR, see Table 4.

#### 3.1.5. 4′,7-Dihydroxyisoflavone (Daidzein) (**5**)

C_15_H_10_O_4_, *t*_R_ 11.32; ^1^H-NMR, see Table 3; ^13^C-NMR, see Table 4.

#### 3.1.6. 7-Aminoflavone (**6**)

C_15_H_11_NO_2_, *t*_R_ 13.56; ^1^H-NMR, see Table 1; ^13^C-NMR, see Table 2.

### 3.2. Microorganism

The description of the collection of the material, propagation of structures of the fungi, as well as genetic identification were detailed described in our previous publications [1,30]. The microorganisms were maintained on potato slants at 4 °C and freshly subcultured before use in the experiments.

### 3.3. Analysis

The course of the biotransformation was evaluated by chromatographic methods (TLC, HPLC). TLC analysis was carried out using TLC Silica gel 60/Kieselguhr F254 plates (Merck, Darmstadt, Germany). The developing system was a mixture of chloroform and methanol in the ratio of 9:1. Compounds were visualized using 5% aluminum chloride solution in ethanol. The plates were observed at two wavelengths: 254 and 365 nm.

HPLC analyses were performed using a Waters 2690 instrument equipped with a Waters 996 photodiode array detector, using an ODS 2 column (4.6 × 250 mm, Waters, Milford, MA, USA) and a Guard-Pak Inserts µBondapak C18 pre-column. Separation conditions were as follows: Gradient elution, using 80% of acetonitrile in 4.5% formic acid solution (eluent A) and 4.5% formic acid (eluent B); flow, 1 mL/min; detection wavelength 280 nm; program: 0–7 min, 10% A 90% B; 7–10 min, 50% A 50% B; 10–13 min, 60% A 40% B; 13–15 min, 70% A 30% B; 15–20 min 80% A 20% B; 20–30 min 90% A 10% B; 30–40 min, 100% A. 

Separation of the products obtained by the scaled-up biotransformation was achieved using 1000 μm preparative TLC silica gel plates (Analtech, Gehrden, Germany). After development of the chromatograms in chloroform:methanol 9:1, compounds were extracted from the selected gel fragments using ethyl acetate (twice) and tetrahydrofuran (once). The extracts were combined and the solvents were removed under reduced pressure.

NMR analysis was carried out using a Bruker Avance 600 MHz NMR spectrometer (Billerica, MA, USA) with an UltraShield Plusmagnet.

Optical rotation was measured using digital polarimeter P-2000-Na (ABL&E-JASCO, Kraków, Poland).

Molecular formulas of products were confirmed using high-pressure liquid chromatography HPLC 1200 Agilent Technologies with DAD, FLD, and Mass detectors Triple Quad LC/MS (Agilent Technologies, Santa Clara, CA, USA).

### 3.4. Screening Procedure

Experiments were carried out using Sabouraud medium (1% peptone, 3% glucose). The microorganism was transferred to a 300 mL flask containing 100 mL of the medium. Pre-incubation was carried out on a rotary shaker (140 rpm) at 25 °C for 72 h. Screening was carried out in 100 mL Erlenmeyer flasks containing 30 mL of Sabouraud liquid medium. The pre-grown culture (0.5 mL) was transferred to a flask and after 72-h incubation, 3 mg of the substrate dissolved in 0.5 mL of tetrahydrofuran was added. The molar concentrations of the substrates were: 0.45 M (flavone), 0.42 M (5-hydroxyflavone, 6-hydroxyflavone, 7-hydroxyflavone, 7-aminoflavone), and 0.39 M (daidzein). We used a separate flask for culture for each sample collection. The biotransformation was carried out under the same conditions as pre-incubation. After 4, 7, and 12 days of biotransformation the mixtures were extracted with 30 mL of ethyl acetate. The extracts were dried over MgSO_4_ (5 min), concentrated in vacuo and analyzed by TLC and HPLC.

Stability of the substrate was evaluated under analogous conditions, without using a biocatalyst.

### 3.5. Scaled-Up Biotransformations

Scaled-up biotransformations were carried out in 2 L flasks containing 500 mL of the medium. The pre-incubation culture (1 mL) was transferred to the flask. After 72 h of incubation, 50 mg of the substrate dissolved in 1 mL of tetrahydrofuran was added. The molar concentrations of the substrates were: 0.45 M (flavone), 0.42 M (5-hydroxyflavone, 6-hydroxyflavone, 7-hydroxyflavone, 7-aminoflavone), and 0.39 M (daidzein). The scale-up biotransformation was carried out under the same conditions as the screening (140 rpm, 25 °C). After complete consumption of the substrate, different for each substrate used, metabolites were extracted three times using each time 200 mL of ethyl acetate. The extracts were dried out using MgSO_4_ and concentrated on a rotary evaporator. Product separation was carried out using preparative TLC plates. Product structure was determined by spectroscopic methods (^1^H-NMR, ^13^C-NMR, COSY, HMBC, HSQC).

The physical and spectral data of the products obtained are presented below (Tables S1–S4) (Supplementary Materials).

#### 3.5.1. Flavone 2′-*O*-β-d-(4′′-*O*-Methyl)-glucopyranoside (**1a**)

C_22_H_22_O_8_, M_w_ = 414.4; *t*_R_ 11.44; ${\left[\alpha \right]}_{D}^{20}=$ −29.7°; ^1^H-NMR, see Table S1; ^13^C-NMR, see Table S2.

#### 3.5.2. Flavone 4′-*O*-β-d-(4′′-*O*-Methyl)-glucopyranoside (**1b**)

C_22_H_22_O_8_, M_w_ = 414.4; *t*_R_ 10.65; ${\left[\alpha \right]}_{D}^{20}=$ −48.0°; ^1^H-NMR, see Table S1; ^13^C-NMR, see Table S2.

#### 3.5.3. 3′-Hydroxyflavone 4′-*O*-β-d-(4′′-*O*-Methyl)-glucopyranoside (**1c**)

C_22_H_22_O_9_, M_w_ = 430.4; *t*_R_ 10.50; ${\left[\alpha \right]}_{D}^{20}=$ −28.0°; ^1^H-NMR, see Table S1; ^13^C-NMR, see Table S2.

#### 3.5.4. 5-Hydroxyflavone 4′-*O*-β-d-(4′′-*O*-Methyl)-glucopyranoside (**2a**)

C_22_H_22_O_9_, M_w_ = 430.4; *t*_R_ 11.72; ${\left[\alpha \right]}_{D}^{20}=$ −76.5°; ^1^H-NMR, see Table S3; ^13^C-NMR, see Table S4.

#### 3.5.5. Flavone 6-*O*-β-d-(4′′-*O*-Methyl)-glucopyranoside (**3a**)

C_22_H_22_O_8_, M_w_ = 414.4; *t*_R_ 11.10; ${\left[\alpha \right]}_{D}^{20}=$ −34.4°; ^1^H-NMR, see Table S3; ^13^C-NMR, see Table S4.

#### 3.5.6. Flavone 7-*O*-β-d-(4′′-*O*-Methyl)-glucopyranoside (**4a**)

C_22_H_22_O_8_, M_w_ = 414.4; *t*_R_ 10.68; ${\left[\alpha \right]}_{D}^{20}=$ −40.3°; ^1^H-NMR, see Table S3; ^13^C-NMR, see Table S4.

#### 3.5.7. 4′-Hydroxyisoflavone 7-*O*-β-d-(4′′-*O*-Methyl)-glucopyranoside (**5a**)

C_22_H_22_O_9_, M_w_ = 430.4; *t*_R_ 10.00; ${\left[\alpha \right]}_{D}^{20}=$ −26.0°; ^1^H-NMR, see Table S3; ^13^C-NMR, see Table S4.

#### 3.5.8. 7-Acetamidoflavone (**6a**)

C_17_H_13_NO_3_, M_w_ = 279.3; *t*_R_ 13.67; ^1^H-NMR, see Table S1; ^13^C-NMR, see Table S2.

#### 3.5.9. 7-Acetamido-4′-hydroxyflavone (**6b**)

C_17_H_13_NO_4_, M_w_ = 295.3; *t*_R_ 11.22; ^1^H-NMR, see Table S1; ^13^C-NMR, see Table S2.

## 4. Conclusions

The catalytic capacity of four strains of entomopathogenic filamentous fungi of the genus *Isaria* was evaluated. Only *I. fumosorosea* KCH J2 was able to effectively carry out biotransformations of all substrates used. In the case of flavone, 5-hydroxyflavone, 6-hydroxyflavone, 7-hydroxyflavone and daidzein we observed the formation of *O*-methylglucosides. 7-Aminoflavone was transformed into two acetamido derivatives. Our study not only allowed us to find new biocatalysts for glycosylation of flavones but also, as a result, we obtained seven new flavonoid derivatives. The substrates and products obtained by us can be used to assess the biological activity and bioavailability of aglycone/glucoside pairs of flavonoids, and in the future to become the basis for the creation of new pharmaceuticals or food additives.

## Figures and Tables

**Table 1:** ^1^H-NMR shifts (δ) of flavone (**1**) and 7-aminoflavone (**6**) and products of their biotransformations (**1a**, **1b**, **1c**, **6a**, **6b**) in Acetone-*d*_6_, 600 MHz (Supplementary Materials).

Proton	Compound						
	1	1a	1b	1c	6	6a	6b
H-3	6.91 (s)	7.15 (s)	6.83 (s)	6.81 (s)	6.69 (s)	6.84 (s)	6.70 (s)
H-5	8.17 (dd) *J*_5,6_ = 7.9, *J* = 1.6	8.16 (d) *J*_5,6_ = 8.0	8.15 (d) *J*_5,6_ = 7.8	8.15 (d) *J*_5,6_ = 7.8	7.86 (d) *J*_5,6_ = 8.4	8.05 (d) *J*_5,6_ = 8.6	8.03 (d) *J*_5,6_ = 8.6
H-6	7.54 (t) *J* = 7.5	7.52 (t) *J* = 7.5	7.52 (t) *J* = 7.4	7.52 (t) *J* = 7.4	6.83 (d) *J*_8,6_ = 2.0	7.46 (dd) *J*_6,5_ = 8.6, *J*_6,8_ = 2.0	7.42 (dd) *J*_6,5_ = 8.6, *J*_6,8_ = 1.9
H-7	7.86 (m)	7.84 (t) *J* = 7.7	7.84 (t) *J* = 7.7	7.85 (t) *J* = 7.8	-	-	-
H-8	7.79 (d) *J*_8,7_ = 8.4	7.73 (d) *J*_8,7_ = 8.4	7.76 (d) *J*_8,7_ = 8.4	7.79 (d) *J*_8,7_ = 8.4	6.82 (t) *J* = 2.7	8.40 (d) *J*_8,6_ = 1.9	8.37 (d) *J*_8,6_ = 1.8
H-2′	8.14 (m)	-	8.09 (d) *J*_2*′*,3*′*_ = 7.9	7.63 (s)	8.05 (dd) *J* = 6.6, 3.0	8.13 (m)	7.08 (m)
H-3′	7.65 (m)	7.44 (d) *J* = 8.3	7.28 (d) *J*_3*′*,2*′*_ = 7.9	-	7.61 (dd) *J* = 6.5, 3.5	7.64 (m)	8.00 (m)
H-4′	7.65 (m)	7.57 (t) *J* = 7.7	-	-	7.61 (dd) *J* = 6.5, 3.5	7.64 (m)	-
H-5′	7.65 (m)	7.27 (t) *J* = 7.5	7.28 (d) *J_5′_,*6′*__* = 7.9	7.38 (d) *J*_5*′*,6*′*_ = 8.3	7.61 (dd) *J* = 6.5, 3.5	7.64 (m)	8.00 (m)
H-6′	8.14 (m)	7.99 (d) *J* = 7.8	8.09 (d) *J*_6,5*′*_ = 7.9	7.60 (d) *J*_6*′*,5*′*_ = 8.4	8.05 (dd) *J* = 6.6, 3.0	8.13 (m)	7.08 (m)
H-1′′	-	5.23 (d) *J* = 7.6	5.14 (d) *J* = 7.7	5.00 (d) *J* = 7.8	-	-	-
H-2′′	-	3.61 (m)	3.56 (t) *J* = 8.4	3.58 (m)	-	-	-
H-3′′	-	3.69 (m)	3.70 (t) *J* = 9.0	3.71 (t) *J* = 9.1	-	-	-
H-4′′	-	3.29 (t) *J* = 9.2	3.28 (t) *J* = 9.3	3.29 (t) *J* = 9.1	-	-	-
H-5′′	-	3.61 (m)	3.59 (m)	3.58 (m)	-	-	-
H-6′′	-	3.89 (d) *J* = 12.2, 3.74 (dd) *J* = 13.6, 3.9	3.90 (d) *J* = 11.9, 3.75 (dd) *J* = 11.6, 4.0	3.91 (d) *J* = 11.5, 3.76 (dd) *J* = 11.3, 3.8	-	-	-
C-4′′-OCH_3_	-	3.61 (m)	3.62 (s)	3.61 (s)	-	-	-
-COCH_3_	-	-	-	-	-	2.21 (s)	2.21 (s)
-NH_2_	-	-	-	-	5.82 (s)	-	-
-NH-	-	-	-	-	-	9.78 (s)	9.73 (s)
-OH	-	-	-	-	-	-	9.27 (s)

**Table 2:** ^13^C-NMR shifts (δ) of flavone (**1**) and 7-aminoflavone (**6**) and products of their biotransformations (**1a**, **1b**, **1c**, **6a**, **6b**) in Acetone-*d*_6_, 600 MHz (Supplementary Materials).

Carbon	Compound						
	1	1a	1b	1c	6	6a	6b
C-2	163.9	161.6	163.8	163.7	162.5	163.8	164.1
C-3	108.0	113.4	106.8	107.2	107.6	107.9	106.0
C-4	178.0	178.3	177.8	177.8	177.1	177.2	177.1
C-4a	124.8	124.7	124.8	124.8	115.1	120.2	120.2
C-5	126.0	125.9	126.0	125.9	127.3	126.6	126.5
C-6	126.2	125.9	126.0	126.0	114.2	117.4	117.1
C-7	134.9	134.7	134.7	134.7	155.1	145.4	145.1
C-8	119.3	119.3	119.2	119.2	99.7	107.5	107.4
C-8a	157.1	157.5	157.1	157.1	159.4	158.0	157.9
C-1′	132.8	122.6	126.2	127.9	133.2	132.9	123.9
C-2′	127.2	156.7	128.8	114.5	126.9	127.1	129.1
C-3′	130.0	116.7	117.7	148.7	129.9	130.0	116.8
C-4′	132.5	133.3	161.4	148.9	131.9	132.3	161.6
C-5′	130.0	123.0	117.7	118.3	129.9	130.0	116.8
C-6′	127.2	130.1	128.8	119.3	126.9	127.1	129.1
C-1′′	-	101.6	101.2	107.2	-	-	-
C-2′′	-	74.7	74.8	74.7	-	-	-
C-3′′	-	78.1	77.9	77.3	-	-	-
C-4′′	-	80.00	80.1	80.0	-	-	-
C-5′′	-	77.2	77.2	77.4	-	-	-
C-6′′	-	61.9	61.9	61.9	-	-	-
C-4′′-OCH_3_	-	60.6	60.6	60.6	-	-	-
-COCH_3_	-	-	-	-	-	169.9	169.8
-COCH_3_	-	-	-	-	-	24.5	24.5

**Table 3:** ^1^H-NMR shifts (δ) of 5-hydroxyflavone (**2**), 6-hydroxyflavone (**3**), 7-hydroxyflavone (**4**) and 4′,7-dihydroxyisoflavone (daidzein) (**5**) and products of their biotransformations (**2a**, **3a**, **4a**, **5a**) in Acetone-*d*_6_, 600 MHz (Supplementary Materials).

Proton	Compound							
	2	2a	3	3a	4	4a	5	5a
H-2	-	-	-	-	-	-	8.16 (s)	8.24 (s)
H-3	6.96 (s)	6.87 (s)	6.85 (s)	6.88 (s)	6.79 (s)	6.84 (s)	-	-
H-5	-	-	7.53 (d) *J*_5,7_ = 3.0	7.73 (d) *J*_5,7_ = 1.9	8.02 (d) *J*_5,6_ = 8.7	8.07 (d) *J*_5,6_ = 8.8	8.09 (d) *J*_5,6_ = 8.7	8.15 (d) *J*_5,6_ = 8.7
H-6	6.84 (d) *J*_6,7_ = 8.2	6.81 (d) *J*_6,7_ = 8.4	-	-	7.03 (dd) *J*_6,5_ = 8.7, *J*_6,8_ = 2.2	7.19 (d) *J*_6,5_ = 8.8	7.02 (d) *J*_6,5_ = 8.8	7.18 (d) *J*_6,5_ = 8.8
H-7	7.72 (t) *J* = 8.3	7.69 (t) *J* = 8.3	7.36 (dd) *J*_7,8_ = 9.0, *J*_7,5_ = 3.0	7.57 (dd) *J*_7,8_ = 8.8, *J*_7,5_ = 2.0	-	-	-	-
H-8	7.21 (d) *J*_8,7_ = 8.4	7.18 (d) *J*_8,7_ = 8.4	7.68 (d) *J*_8,7_ = 9.0	7.76 (d) *J*_8,7_ = 9.0	7.11 (d) *J*_8,6_ = 2.2	7.40 (s)	6.93 (m)	7.22 (s)
H-2′	8.16 (m)	8.10 (d) *J*_2′,3′_ = 8.6	8.12 (m)	8.14 (d) *J* = 7.1	8.10 (dd) *J* = 6.5, 2.9	8.10 (d) *J* = 7.3	7.51 (d) *J* = 8.3	7.52 (d) *J*_2′,3′_ = 8.0
H-3′	7.67 (m)	7.29 (d) *J*_3′,2′_ = 8.6	7.64 (m)	7.64 (m)	7.63 (m)	7.64 (d) *J* = 5.9	6.91 (m)	6.93 (d) *J*_3′,2′_ = 7.9
H-4′	7.67 (m)	-	7.64 (m)	7.64 (m)	7.63 (m)	7.64 (d) *J* = 5.9	-	-
H-5′	7.67 (m)	7.29 (d) *J*_5′,6′_ = 8.6	7.64 (m)	7.64 (m)	7.63 (m)	7.64 (d) *J* = 5.9	6.91 (m)	6.93 (d) *J*_5′,6′_ = 7.9
H-6′	8.16 (m)	8.10 (d) *J*_6′,5′_ = 8.6	8.12 (m)	8.14 (d) *J* = 7.1	8.10 (dd) *J* = 6.5, 2.9	8.10 (d) *J* = 7.3	7.51 (d) *J* = 8.3	7.52 (d) *J*_6′,5′_ = 8.0
H-1′′	-	5.15 (d) *J* = 7.7	-	5.11 (d) *J =* 7.8	-	5.24 (d) *J* = 7.7	-	5.21 (d) *J* = 7.7
H-2′′	-	3.56 (m)	-	3.54 (m)	-	3.58 (t) *J* = 8.4	-	3.57 (t) *J* = 8.2
H-3′′	-	3.70 (m)	-	3.73 (m)	-	3.72 (t) *J* = 9.3	-	3.71 (m)
H-4′′	-	3.27 (m)	-	3.31 (t) *J* = 9.4	-	3.30 (t) *J* = 9.3	-	3.28 (t) *J* = 9.3
H-5′′	-	3.58 (m)	-	3.60 (dd) *J* = 9.9, 2.5	-	3.65 (m)	-	3.67 (m)
H-6′′	-	3.89 (m) 3.76 (m)	-	3.89 (t) *J* = 10.7 3.67 (m)	-	3.92 (d) *J* = 11.3 3.75 (m)	-	3.92 (m) 3.75 (m)
C-4′′-OCH_3_	-	3.61 (s)	-	3.62 (s)	-	3.62 (s)	-	3.62 (s)
-OH	12.74	-	9.14	-	9.86	-	-	-

**Table 4:** ^13^C-NMR shifts (δ) of 5-hydroxyflavone (**2**), 6-hydroxyflavone (**3**), 7-hydroxyflavone (**4**) and 4′,7-dihydroxyisoflavone (daidzein) (**5**) and products of their biotransformations (**2a**, **3a**, **4a**, **5a**) in Acetone-*d*_6_, 600 MHz (Supplementary Materials).

Carbon	Compound							
	2	2a	3	3a	4	4a	5	5a
C-2	164.7	165.4	163.6	163.8	163.4	163.7	153.1	153.5
C-3	105.7	105.3	107.0	102.7	107.7	107.9	125.2	124.2
C-4	183.6	184.3	177.9	177.7	177.3	177.3	175.7	175.7
C-4a	110.6	111.3	125.7	125.5	118.0	119.6	118.3	120.2
C-5	160.8	161.7	108.9	111.1	127.7	127.4	128.4	128.1
C-6	111.1	111.8	155.9	156.0	115.6	116.3	115.8	116.4
C-7	135.8	136.4	123.7	124.9	163.5	162.9	163.6	162.6
C-8	107.2	108.0	120.6	120.6	103.6	104.9	103.1	104.4
C-8a	156.5	157.3	151.1	152.5	158.9	158.5	158.1	158.3
C-1′	131.2	125.5	133.0	132.8	132.9	132.8	124.4	125.4
C-2′	126.6	129.2	127.1	127.2	127.0	127.1	131.1	131.1
C-3′	129.2	117.7	130.0	130.0	129.9	130.0	115.8	115.9
C-4′	132.2	161.8	132.3	132.5	132.2	132.4	158.9	158.4
C-5′	129.2	117.7	130.0	130.0	129.9	130.0	115.8	115.9
C-6′	126.6	129.2	127.1	127.2	127.0	127.1	131.1	131.1
C-1′′	-	101.1	-	102.2	-	101.3	-	101.3
C-2′′	-	74.9	-	74.89	-	74.8	-	74.8
C-3′′	-	77.9	-	77.6	-	77.8	-	77.9
C-4′′	-	80.1	-	79.9	-	80.0	-	80.0
C-5′′	-	77.2	-	77.1	-	77.3	-	77.3
C-6′′	-	62.0	-	61.9	-	61.9	-	62.0
C-4′′-OCH_3_	-	60.6	-	60.6	-	60.6	-	60.6

## Supplementary Materials

The supplementary materials are available online.
