# Supplementary material for: Biotransformations of Flavones and an Isoflavone (Daidzein) in Cultures of Entomopathogenic Filamentous Fungi
Source: Molecules. 2018 Jun 5;23(6):1356. doi: 10.3390/molecules23061356 (PMC6100588; doi:10.3390/molecules23061356)

Supplementary data

Biotransformations of flavones and an isoflavone (daidzein) in cultures of entomopathogenic filamentous fungi

Monika Dymarska*, Tomasz Janeczko and Edyta Kostrzewa-Susłow

Department of Chemistry, Faculty of Biotechnology and Food Science, Wrocław University of Environmental and Life Sciences, Wrocław, Poland; janeczko13@interia.pl (T.J.); ekostrzew@gmail.com (E.K.-S.)

* Correspondence: monika.dymarska@gmail.com

Content

Figure S1. ^{1}H NMR spectrum of flavone (1) (Acetone-d_6, 600 MHz)

Figure S2. ^{13}C NMR spectrum of flavone (1) (Acetone-d_6, 151 MHz)

Figure S3. ^{13}C NMR spectrum of flavone (1) (Acetone-d_6, 151 MHz)

Figure S4. HSQC NMR spectrum of flavone (1) (Acetone-d_6, 151 MHz)

Figure S5. HMBC NMR spectrum of flavone (1) (Acetone-d_6, 151 MHz)

Figure S6. ^{1}H NMR spectrum of flavone 2′-*O*-*β*-D-(4″-*O*-methyl)-glucopyranoside (1a) (Acetone-d_6, 600 MHz)

Figure S7. ^{1}H NMR spectrum of flavone 2′-*O*-*β*-D-(4″-*O*-methyl)-glucopyranoside (1a) (Acetone-d_6, 600 MHz)

Figure S8. ^{13}C NMR spectrum of flavone 2′-*O*-*β*-D-(4″-*O*-methyl)-glucopyranoside (1a) (Acetone-d_6, 151 MHz)

Figure S9. HSQC NMR spectrum of flavone 2′-*O*-*β*-D-(4″-*O*-methyl)-glucopyranoside (1a) (Acetone-d_6, 151 MHz)

Figure S10. HMBC NMR spectrum of flavone 2′-*O*-*β*-D-(4″-*O*-methyl)-glucopyranoside (1a) (Acetone-d_6, 151 MHz)

Figure S11. ^{1}H NMR spectrum of flavone 4′-*O*-*β*-D-(4″-*O*-methyl)-glucopyranoside (1b) (Acetone-d_6, 600 MHz)

Figure S12. ^{1}H NMR spectrum of flavone 4′-*O*-*β*-D-(4″-*O*-methyl)-glucopyranoside (1b) (Acetone-d_6, 600 MHz)

Figure S13. ^{13}C NMR spectrum of flavone 4′-*O*-*β*-D-(4″-*O*-methyl)-glucopyranoside (1b) (Acetone-d$_6$, 151 MHz)
Figure S14. HSQC NMR spectrum of flavone 4′-*O*-*β*-D-(4″-*O*-methyl)-glucopyranoside (1b) (Acetone-d$_6$, 151 MHz)
Figure S15. HMBC NMR spectrum of flavone 4′-*O*-*β*-D-(4″-*O*-methyl)-glucopyranoside (1b) (Acetone-d$_6$, 151 MHz)
Figure S16. ^{1}H NMR spectrum of 3′-hydroxyflavone 4′-*O*-*β*-D-(4″-*O*-methyl)-glucopyranoside (1c) (Acetone-d$_6$, 600 MHz)
Figure S17. ^{1}H NMR spectrum of 3′-hydroxyflavone 4′-*O*-*β*-D-(4″-*O*-methyl)-glucopyranoside (1c) (Acetone-d$_6$, 600 MHz)
Figure S18. ^{13}C NMR spectrum of 3′-hydroxyflavone 4′-*O*-*β*-D-(4″-*O*-methyl)-glucopyranoside (1c) (Acetone-d$_6$, 151 MHz)
Figure S19. HSQC NMR spectrum of 3′-hydroxyflavone 4′-*O*-*β*-D-(4″-*O*-methyl)-glucopyranoside (1c) (Acetone-d$_6$, 151 MHz)
Figure S20. HMBC NMR spectrum of 3′-hydroxyflavone 4′-*O*-*β*-D-(4″-*O*-methyl)-glucopyranoside (1c) (Acetone-d$_6$, 151 MHz)
Figure S21. ^{1}H NMR spectrum of 5-hydroxyflavone (2) (Acetone-d$_6$, 600 MHz)
Figure S22. ^{13}C NMR spectrum of 5-hydroxyflavone (2) (Acetone-d$_6$, 151 MHz)
Figure S23. HSQC NMR spectrum of 5-hydroxyflavone (2) (Acetone-d$_6$, 151 MHz)
Figure S24. HMBC NMR spectrum of 5-hydroxyflavone (2) (Acetone-d$_6$, 151 MHz)
Figure S25. ^{1}H NMR spectrum of 5-hydroxyflavone 4′-*O*-*β*-D-(4″-*O*-methyl)-glucopyranoside (2a) (Acetone-d$_6$, 600 MHz)
Figure S26. ^{1}H NMR spectrum of 5-hydroxyflavone 4′-*O*-*β*-D-(4″-*O*-methyl)-glucopyranoside (2a) (Acetone-d$_6$, 600 MHz)
Figure S27. ^{13}C NMR spectrum of 5-hydroxyflavone 4′-*O*-*β*-D-(4″-*O*-methyl)-glucopyranoside (2a) (Acetone-d$_6$, 151 MHz)
Figure S28. HSQC NMR spectrum of 5-hydroxyflavone 4′-*O*-*β*-D-(4″-*O*-methyl)-glucopyranoside (2a) (Acetone-d$_6$, 151 MHz)
Figure S29. HMBC NMR spectrum of 5-hydroxyflavone 4′-*O*-*β*-D-(4″-*O*-methyl)-glucopyranoside (2a) (Acetone-d$_6$, 151 MHz)
Figure S30. ^{1}H NMR spectrum of 6-hydroxyflavone (3) (Acetone-d$_6$, 600 MHz)
Figure S31. ^{13}C NMR spectrum of 6-hydroxyflavone (3) (Acetone-d$_6$, 151 MHz)
Figure S32. HSQC NMR spectrum of 6-hydroxyflavone (3) (Acetone-d$_6$, 151 MHz)
Figure S33. HMBC NMR spectrum of 6-hydroxyflavone (3) (Acetone-d$_6$, 151 MHz)
Figure S34. ^{1}H NMR spectrum of flavone 6-*O*-*β*-D-(4″-*O*-methyl)-glucopyranoside (3a) (Acetone-d$_6$, 600 MHz)
Figure S35. ^{1}H NMR spectrum of flavone 6-*O*-*β*-D-(4″-*O*-methyl)-glucopyranoside (3a) (Acetone-d$_6$, 600 MHz)
Figure S36. ^{13}C NMR spectrum of flavone 6-*O*-*β*-D-(4″-*O*-methyl)-glucopyranoside (3a) (Acetone-d$_6$, 151 MHz)
Figure S37. HSQC NMR spectrum of flavone 6-*O*-*β*-D-(4″-*O*-methyl)-glucopyranoside (3a) (Acetone-d$_6$, 151 MHz)
Figure S38. HMBC NMR spectrum of flavone 6-*O*-*β*-D-(4″-*O*-methyl)-glucopyranoside (3a) (Acetone-d$_6$, 151 MHz)
Figure S39. ^{1}H NMR spectrum of 7-hydroxyflavone (4) (Acetone-d$_6$, 600 MHz)
Figure S40. ^{13}C NMR spectrum of 7-hydroxyflavone (4) (Acetone-d$_6$, 151 MHz)
Figure S41. HSQC NMR spectrum of 7-hydroxyflavone (4) (Acetone-d$_6$, 151 MHz)
Figure S42. HMBC NMR spectrum of 7-hydroxyflavone (4) (Acetone-d$_6$, 151 MHz)

Figure S43. ^{1}H NMR spectrum of flavone 7-*O*-*β*-D-(4″-*O*-methyl)-glucopyranoside (4a) (Acetone-d_6, 600 MHz)
Figure S44. ^{1}H NMR spectrum of flavone 7-*O*-*β*-D-(4″-*O*-methyl)-glucopyranoside (4a) (Acetone-d_6, 600 MHz)
Figure S45. ^{13}C NMR spectrum of flavone 7-*O*-*β*-D-(4″-*O*-methyl)-glucopyranoside (4a) (Acetone-d_6, 151 MHz)
Figure S46. HSQC NMR spectrum of flavone 7-*O*-*β*-D-(4″-*O*-methyl)-glucopyranoside (4a) (Acetone-d_6, 151 MHz)
Figure S47. HMBC NMR spectrum of flavone 7-*O*-*β*-D-(4″-*O*-methyl)-glucopyranoside (4a) (Acetone-d_6, 151 MHz)
Figure S48. ^{1}H NMR spectrum of 4′,7-dihydroxyisoflavone (5) (Acetone-d_6, 600 MHz)
Figure S49. ^{13}C NMR spectrum of 4′,7-dihydroxyisoflavone (5) (Acetone-d_6, 151 MHz)
Figure S50. HSQC NMR spectrum of 4′,7-dihydroxyisoflavone (5) (Acetone-d_6, 151 MHz)
Figure S51. HMBC NMR spectrum of 4′,7-dihydroxyisoflavone (5) (Acetone-d_6, 151 MHz)
Figure S52. ^{1}H NMR spectrum of 4′-hydroxyisoflavone 7-*O*-*β*-D-(4″-*O*-methyl)-glucopyranoside (5a) (Acetone-d_6, 600 MHz)
Figure S53. ^{1}H NMR spectrum of 4′-hydroxyisoflavone 7-*O*-*β*-D-(4″-*O*-methyl)-glucopyranoside (5a) (Acetone-d_6, 600 MHz)
Figure S54. ^{13}C NMR spectrum of 4′-hydroxyisoflavone 7-*O*-*β*-D-(4″-*O*-methyl)-glucopyranoside (5a)(Acetone-d_6, 151 MHz)
Figure S55. HSQC NMR spectrum of 4′-hydroxyisoflavone 7-*O*-*β*-D-(4″-*O*-methyl)-glucopyranoside (5a) (Acetone-d_6, 151 MHz)
Figure S56. HMBC NMR spectrum of 4′-hydroxyisoflavone 7-*O*-*β*-D-(4″-*O*-methyl)-glucopyranoside (5a) (Acetone-d_6, 151 MHz)
Figure S57. ^{1}H NMR spectrum of 7-aminoflavone (6) (Acetone-d_6, 600 MHz)
Figure S58. ^{13}C NMR spectrum of 7-aminoflavone (6) (Acetone-d_6, 151 MHz)
Figure S59. HSQC NMR spectrum of 7-aminoflavone (6) (Acetone-d_6, 151 MHz)
Figure S60. HMBC NMR spectrum of 7-aminoflavone (6) (Acetone-d_6, 151 MHz)
Figure S61. ^{1}H NMR spectrum of 7-acetamidoflavone (6a) (Acetone-d_6, 600 MHz)
Figure S62. ^{13}C NMR spectrum of 7-acetamidoflavone (6a) (Acetone-d_6, 151 MHz)
Figure S63. HSQC NMR spectrum of 7-acetamidoflavone (6a) (Acetone-d_6, 151 MHz)
Figure S64. HMBC NMR spectrum of 7-acetamidoflavone (6a) (Acetone-d_6, 151 MHz)
Figure S65. ^{1}H NMR spectrum of 4′-hydroxy-7-acetamidoflavone (6b) (Acetone-d_6, 600 MHz)
Figure S66. ^{13}C NMR spectrum of 4′-hydroxy-7-acetamidoflavone (6b) (Acetone-d_6, 151 MHz)
Figure S67. HSQC NMR spectrum of 4′-hydroxy-7-acetamidoflavone (6b) (Acetone-d_6, 151 MHz)
Figure S68. HMBC NMR spectrum of 4′-hydroxy-7-acetamidoflavone (6b) (Acetone-d_6, 151 MHz)
Figure S69. HPLC chromatogram of flavone (1)
Figure S70. UV maxima of flavone (1) obtained after HPLC analysis
Figure S71. HPLC chromatogram of flavone 2′-*O*-*β*-D-(4″-*O*-methyl)-glucopyranoside (1a)
Figure S72. UV maxima of flavone 2′-*O*-*β*-D-(4″-*O*-methyl)-glucopyranoside (1a) obtained after HPLC analysis
Figure S73. HPLC chromatogram of flavone 4′-*O*-*β*-D-(4″-*O*-methyl)-glucopyranoside (1b)
Figure S74. UV maxima of flavone 4′-*O*-*β*-D-(4″-*O*-methyl)-glucopyranoside (1b) obtained after HPLC analysis
Figure S75. HPLC chromatogram of 3′-hydroxyflavone 4′-*O*-*β*-D-(4″-*O*-methyl)-glucopyranoside (1c)
Figure S76. UV maxima of 3′-hydroxyflavone 4′-*O*-*β*-D-(4″-*O*-methyl)-glucopyranoside (1c) obtained after HPLC analysis

Figure S77. HPLC chromatogram of 5-hydroxyflavone (2)
Figure S78. UV maxima of 5-hydroxyflavone (2) obtained after HPLC analysis
Figure S79. HPLC chromatogram of 5-hydroxyflavone 4′-*O*-*β*-D-(4″-*O*-methyl)-glucopyranoside (2a)
Figure S80. UV maxima of 5-hydroxyflavone 4′-*O*-*β*-D-(4″-*O*-methyl)-glucopyranoside (2a) obtained after HPLC analysis
Figure S81. HPLC chromatogram of 6-hydroxyflavone (3)
Figure S82. UV maxima of 6-hydroxyflavone (3) obtained after HPLC analysis
Figure S83. HPLC chromatogram of flavone 6-*O*-*β*-D-(4″-*O*-methyl)-glucopyranoside (3a)
Figure S84. UV maxima of flavone 6-*O*-*β*-D-(4″-*O*-methyl)-glucopyranoside (3a) obtained after HPLC analysis
Figure S85. HPLC chromatogram of 7-hydroxyflavone (4)
Figure S86. UV maxima of 7-hydroxyflavone (4) obtained after HPLC analysis
Figure S87. HPLC chromatogram of flavone 7-*O*-*β*-D-(4″-*O*-methyl)-glucopyranoside (4a)
Figure S88. UV maxima of flavone 7-*O*-*β*-D-(4″-*O*-methyl)-glucopyranoside (4a) obtained after HPLC analysis
Figure S89. HPLC chromatogram of 4′,7-dihydroxyisoflavone (5)
Figure S90. UV maxima of 4′,7-dihydroxyisoflavone (5) obtained after HPLC analysis
Figure S91. HPLC chromatogram of 4′-hydroxyisoflavone 7-*O*-*β*-D-(4″-*O*-methyl)-glucopyranoside (5a)
Figure S92. UV maxima of 4′-hydroxyisoflavone 7-*O*-*β*-D-(4″-*O*-methyl)-glucopyranoside (5a) obtained after HPLC analysis
Figure S93. HPLC chromatogram of 7-aminoflavone (6)
Figure S94. UV maxima of 7-aminoflavone (6) obtained after HPLC analysis
Figure S95. HPLC chromatogram of 7-acetamidoflavone (6a)
Figure S96. UV maxima of 7-acetamidoflavone (6a) obtained after HPLC analysis
Figure S97. HPLC chromatogram of 4′-hydroxy-7-acetamidoflavone (6b)
Figure S98. UV maxima of 4′-hydroxy-7-acetamidoflavone (6b) obtained after HPLC analysis

Figure S1. ^{1}H NMR spectrum of flavone (1) (Acetone-d$_6$, 600 MHz)

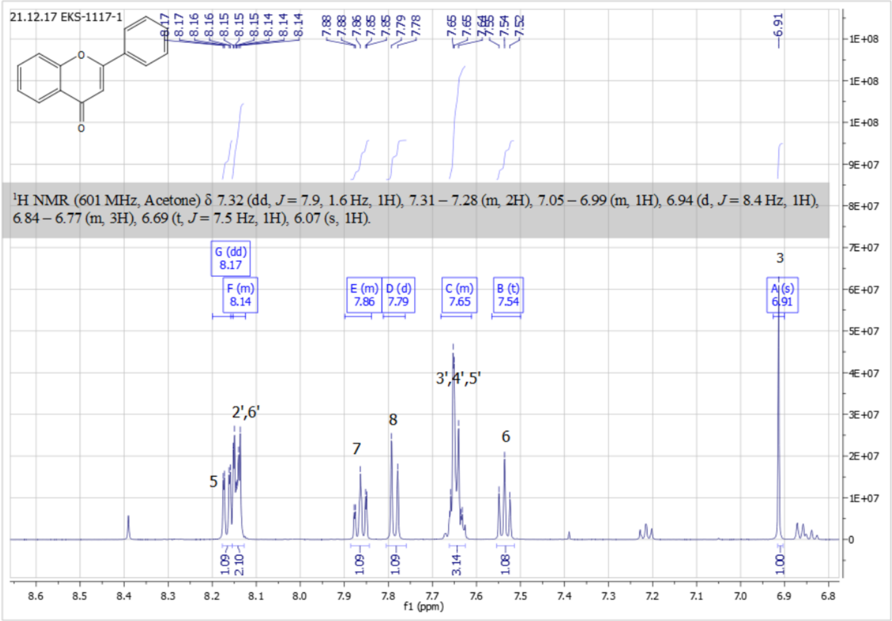

Figure S2. ^{13}C NMR spectrum of flavone (1) (Acetone-d$_6$, 151 MHz)

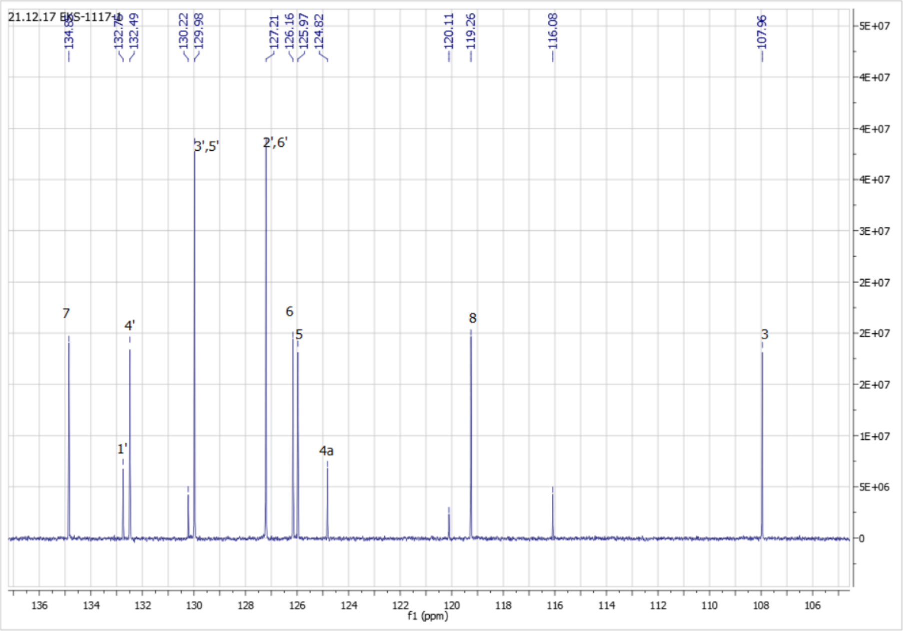

Figure S3. ^{13}C NMR spectrum of flavone (1) (Acetone-d_6, 151 MHz)

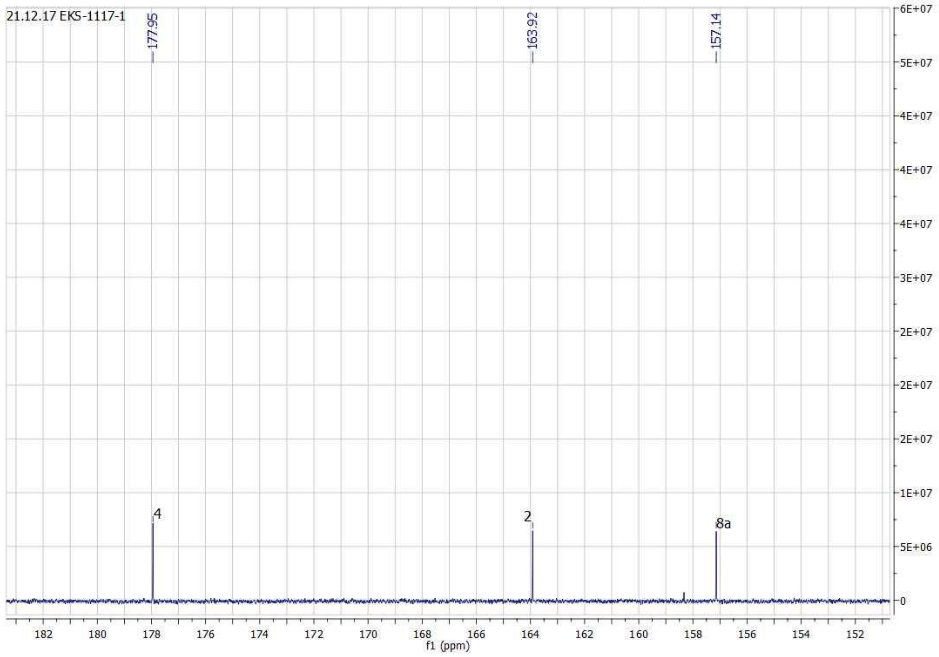

Figure S4. HSQC NMR spectrum of flavone (1) (Acetone-d_6, 151 MHz)

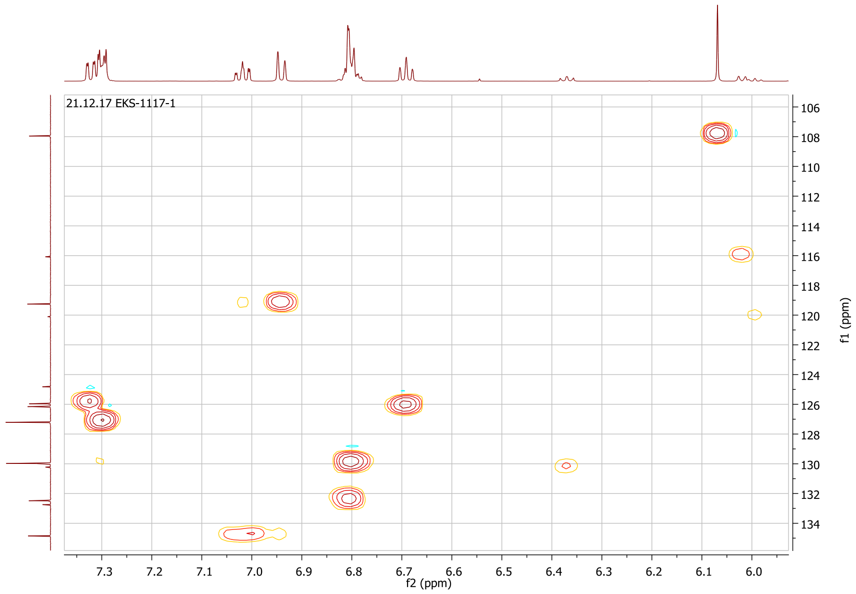

Figure S5. HMBC NMR spectrum of flavone (1) (Acetone-d6, 151 MHz)

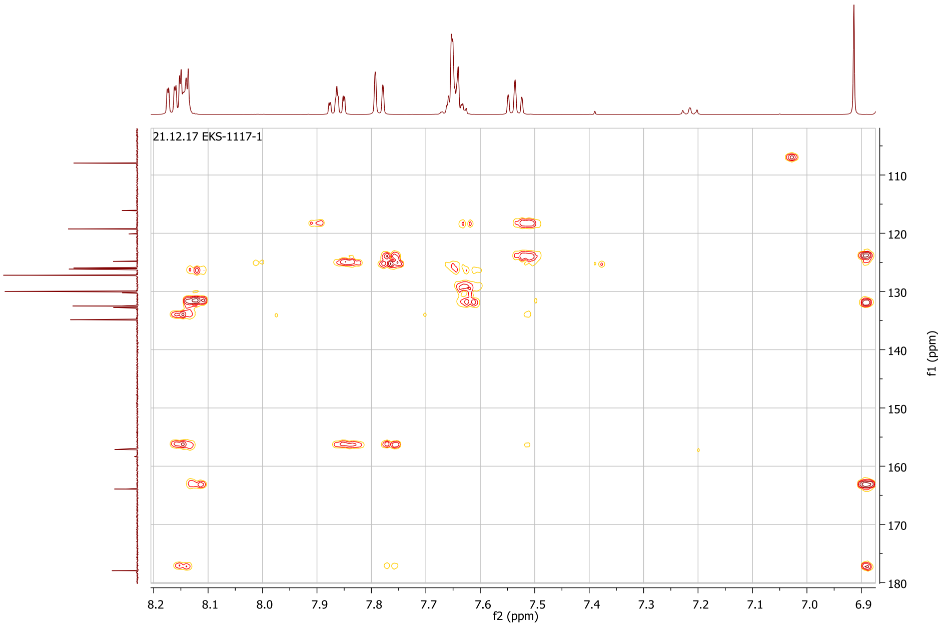

Figure S6. ^{1}H NMR spectrum of flavone 2′-*O*-*β*-D-(4″-*O*-methyl)-glucopyranoside (1a) (Acetone-d6, 600 MHz)

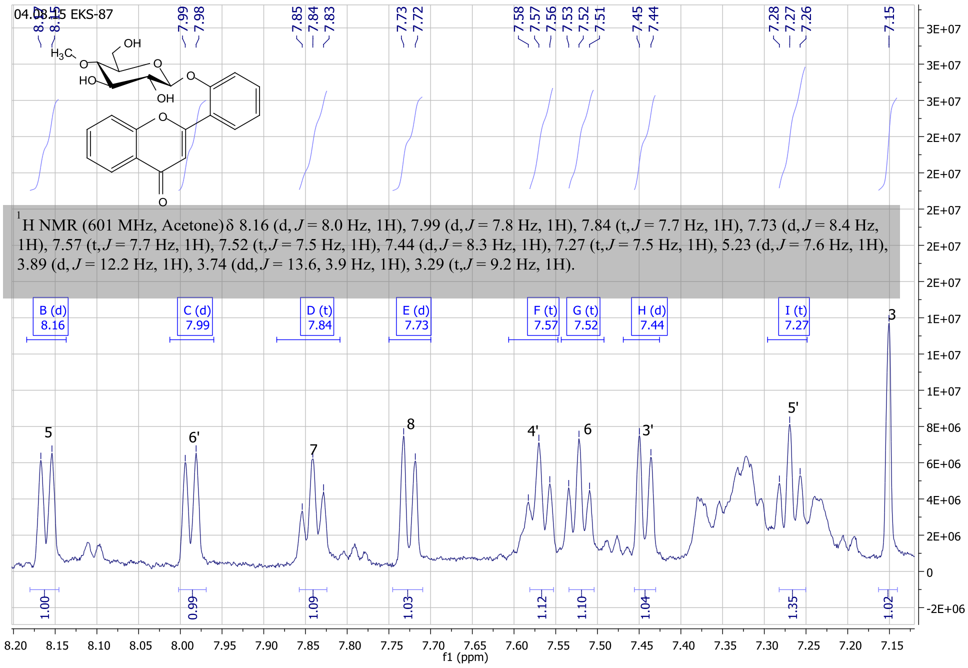

Figure S7. ^{1}H NMR spectrum of flavone 2′-*O*-*β*-D-(4″-*O*-methyl)-glucopyranoside (1a) (Acetone-d6, 600 MHz)

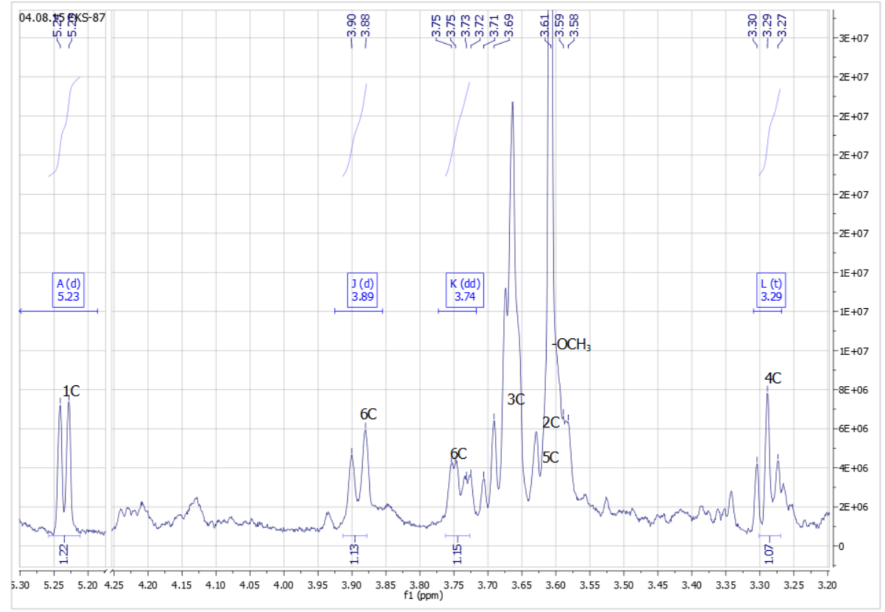

Figure S8. ^{13}C NMR spectrum of flavone 2′-*O*-*β*-D-(4″-*O*-methyl)-glucopyranoside (1a) (Acetone-d6, 151 MHz)

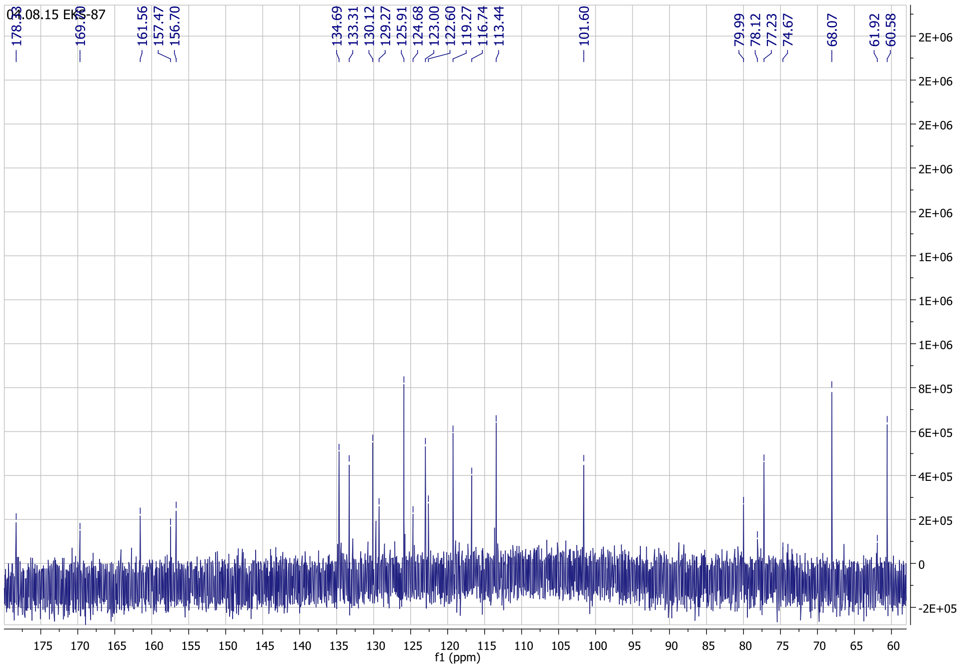

Figure S9. HSQC NMR spectrum of flavone 2′-*O*-*β*-D-(4″-*O*-methyl)-glucopyranoside (1a) (Acetone-d6, 151 MHz)

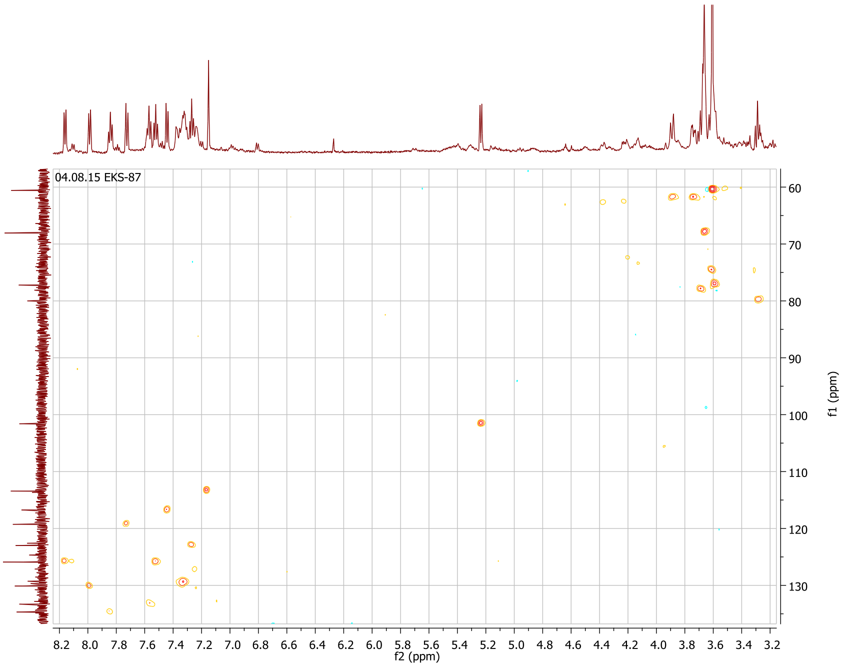

Figure S10. HMBC NMR spectrum of flavone 2′-*O*-*β*-D-(4″-*O*-methyl)-glucopyranoside (1a) (Acetone-d6, 151 MHz)

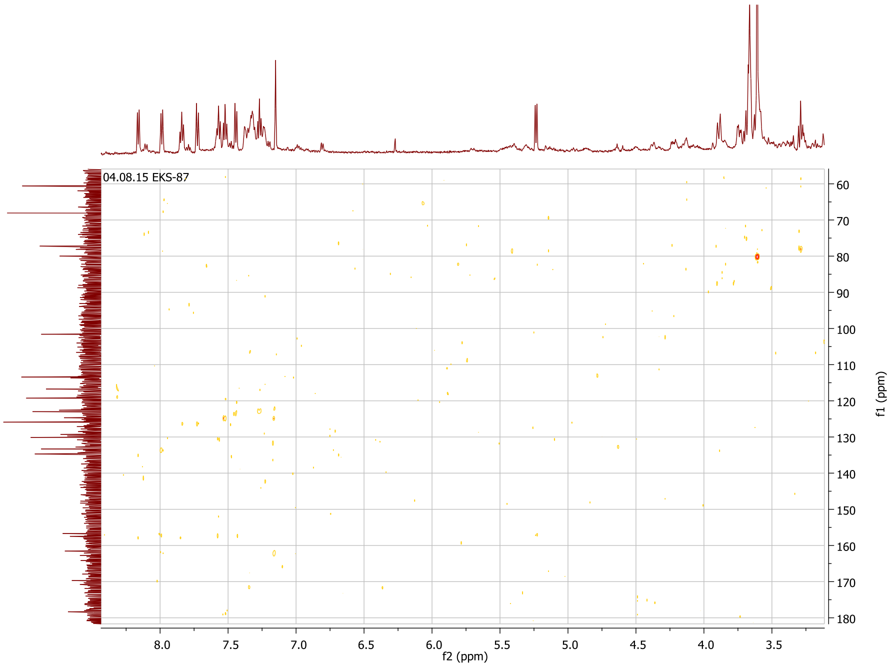

Figure S11. ^{1}H NMR spectrum of flavone 4′-*O*-*β*-D-(4″-*O*-methyl)-glucopyranoside (1b) (Acetone-d$_6$, 600 MHz)

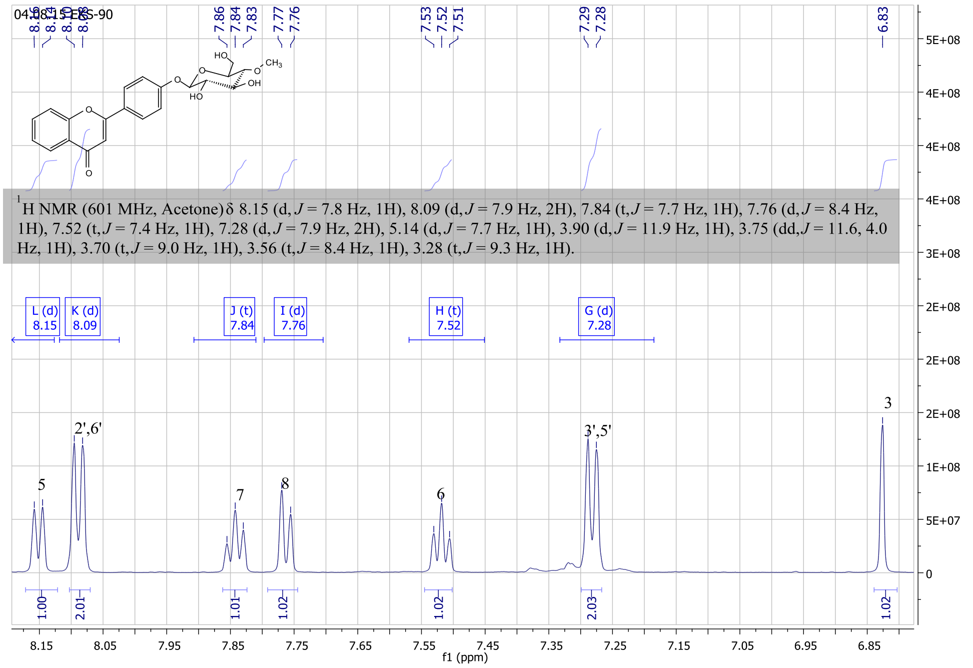

Figure S12. ^{1}H NMR spectrum of flavone 4′-*O*-*β*-D-(4″-*O*-methyl)-glucopyranoside (1b) (Acetone-d$_6$, 600 MHz)

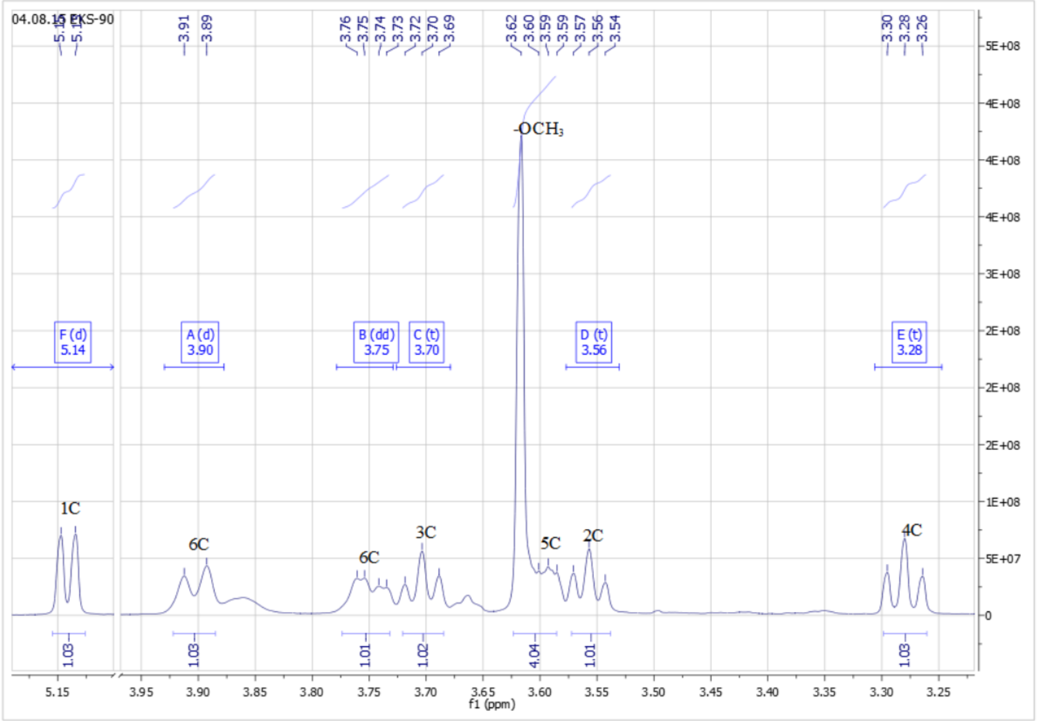

Figure S13. ^{13}C NMR spectrum of flavone 4′-*O*-*β*-D-(4″-*O*-methyl)-glucopyranoside (1b) (Acetone-d$_6$, 151 MHz)

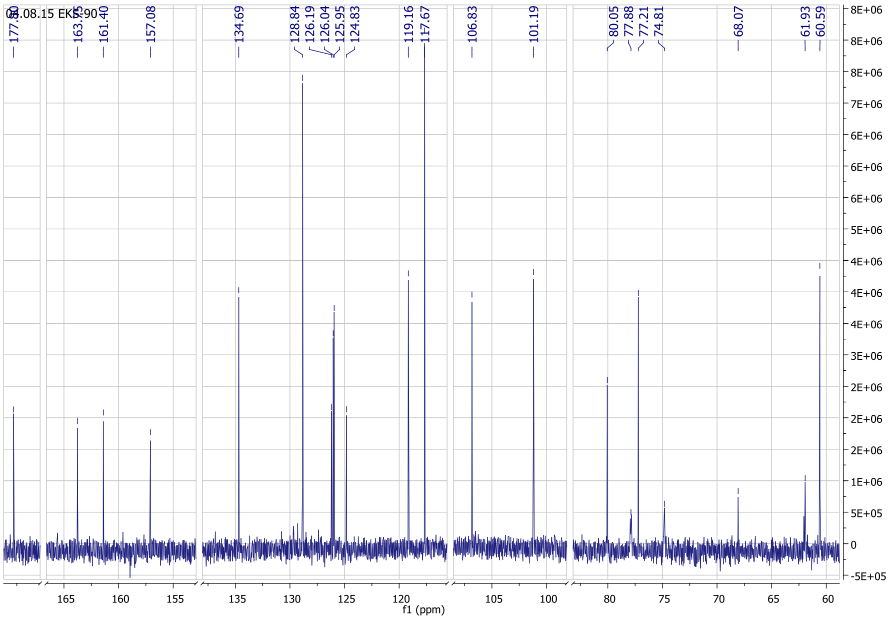

Figure S14. HSQC NMR spectrum of flavone 4′-*O*-*β*-D-(4″-*O*-methyl)-glucopyranoside (1b) (Acetone-d$_6$, 151 MHz)

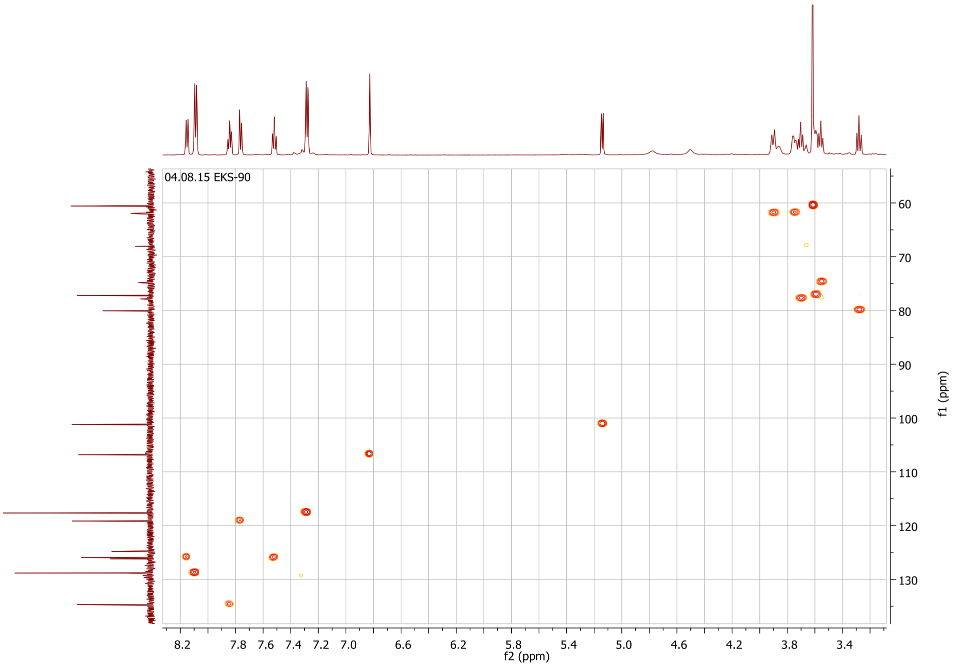

Figure S15. HMBC NMR spectrum of flavone 4′-*O*-*β*-D-(4″-*O*-methyl)-glucopyranoside (1b) (Acetone-d6, 151 MHz)

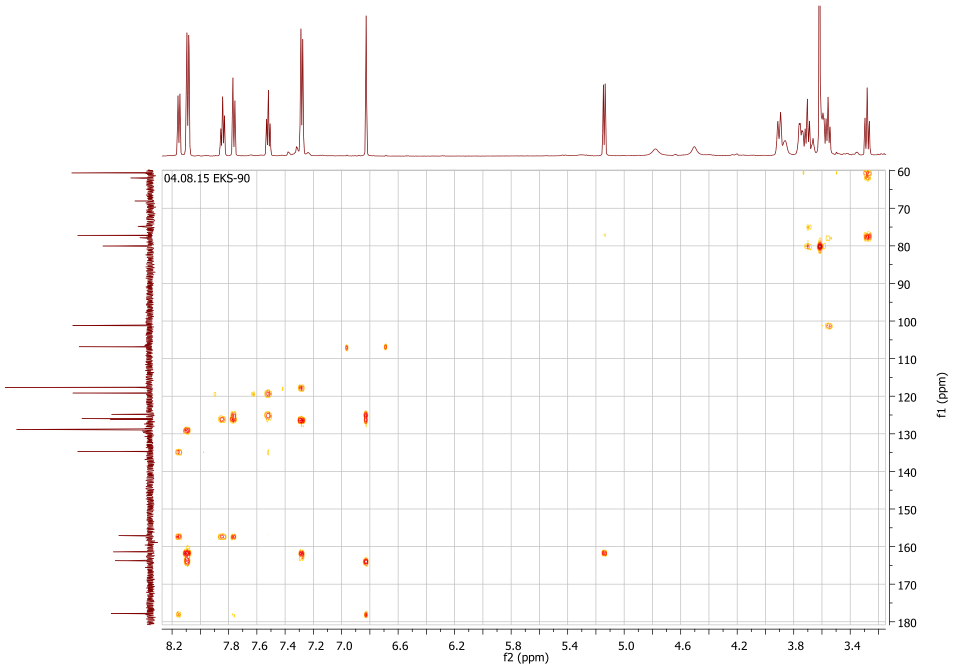

Figure S16. ^{1}H NMR spectrum of 3′-hydroxyflavone 4′-*O*-*β*-D-(4″-*O*-methyl)-glucopyranoside (1c) (Acetone-d6, 600 MHz)

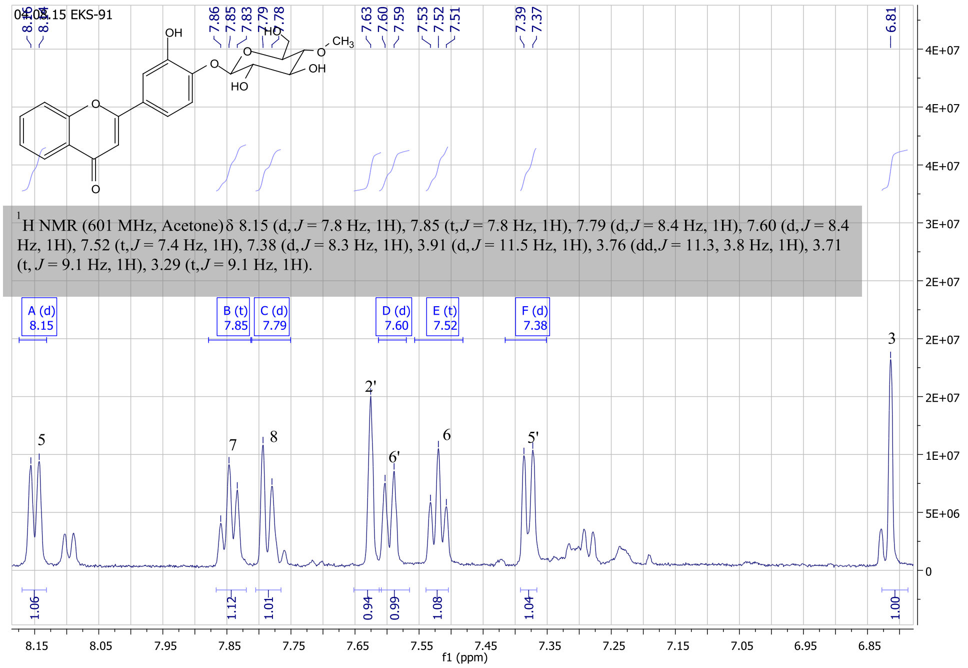

Figure S17. ^{1}H NMR spectrum of 3′-hydroxyflavone 4′-*O*-*β*-D-(4″-*O*-methyl)-glucopyranoside (1c) (Acetone-d$_6$, 600 MHz)

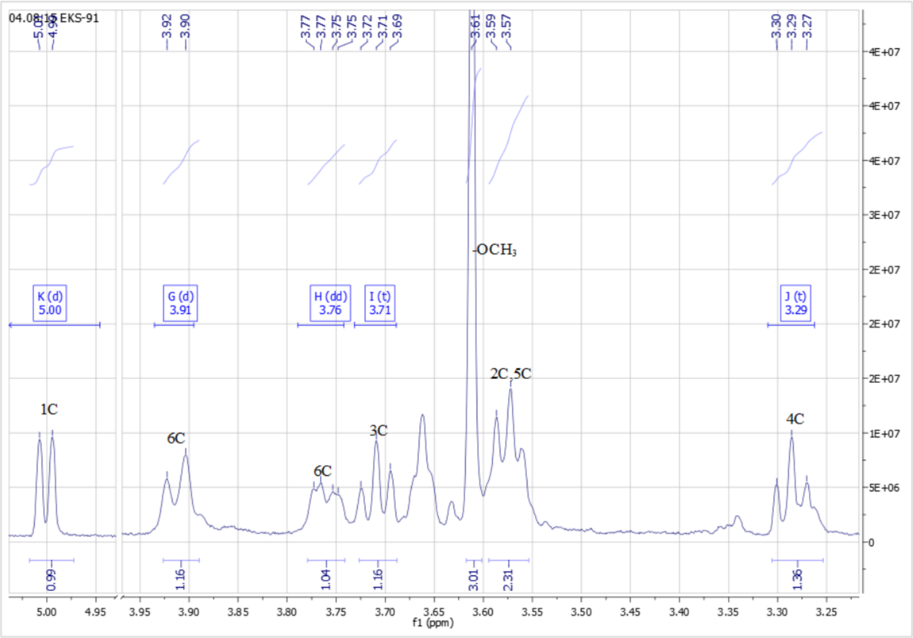

Figure S18. ^{13}C NMR spectrum of 3′-hydroxyflavone 4′-*O*-*β*-D-(4″-*O*-methyl)-glucopyranoside (1c) (Acetone-d$_6$, 151 MHz)

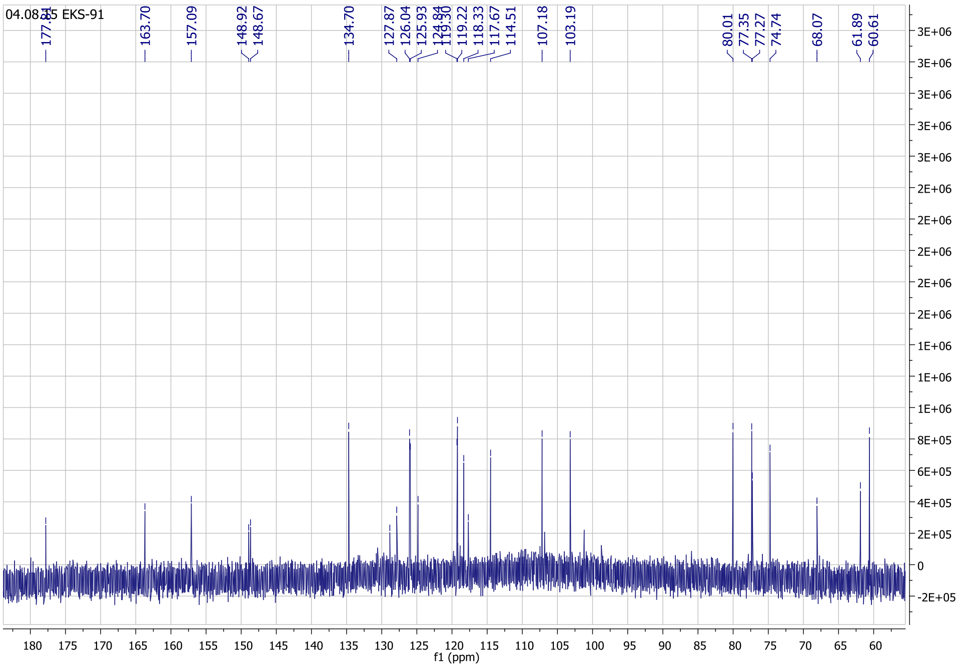

Figure S19. HSQC NMR spectrum of 3′-hydroxyflavone 4′-*O*-*β*-D-(4″-*O*-methyl)-glucopyranoside (1c) (Acetone-d6, 151 MHz)

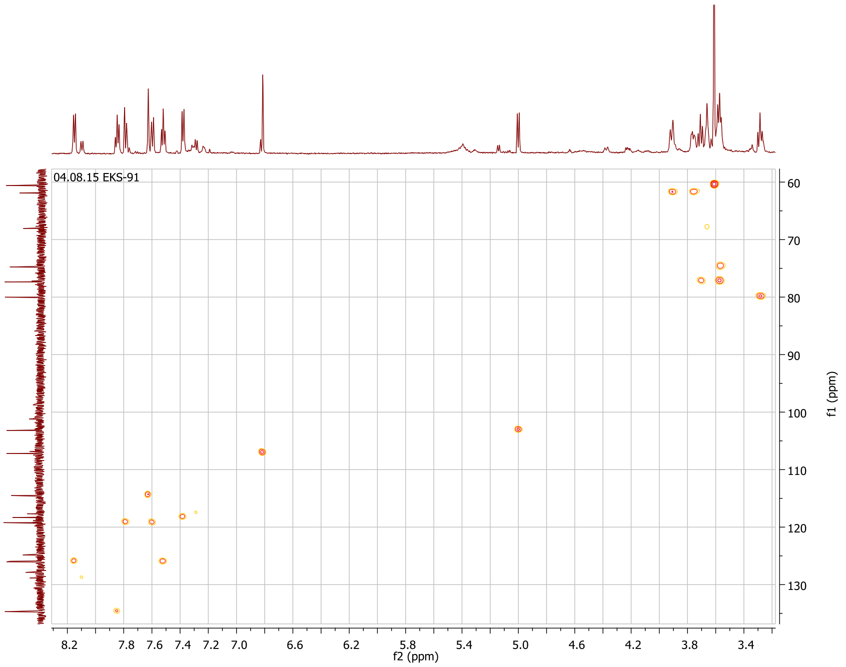

Figure S20. HMBC NMR spectrum of 3′-hydroxyflavone 4′-*O*-*β*-D-(4″-*O*-methyl)-glucopyranoside (1c) (Acetone-d6, 151 MHz)

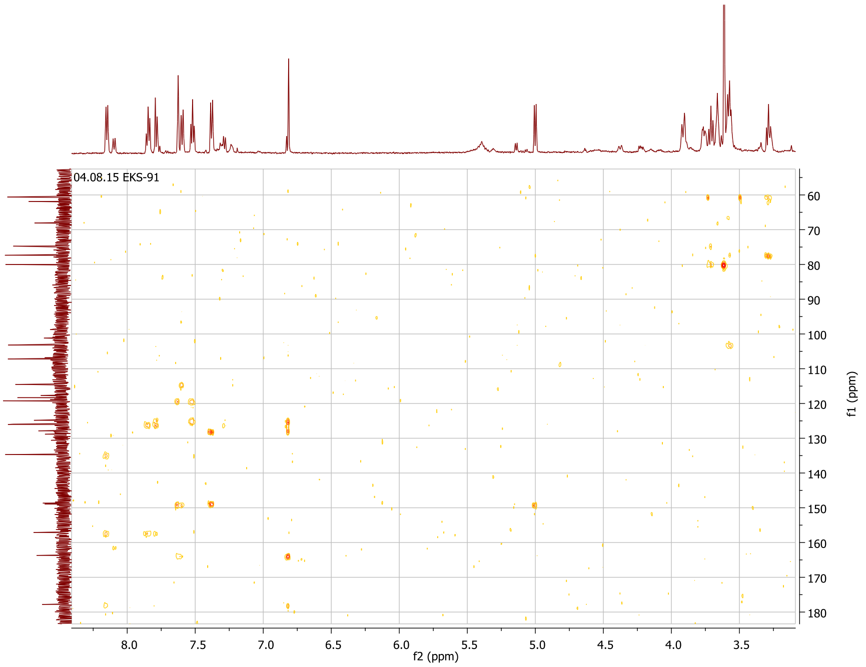

Figure S21. ^{1}H NMR spectrum of 5-hydroxyflavone (2) (Acetone-d$_6$, 600 MHz)

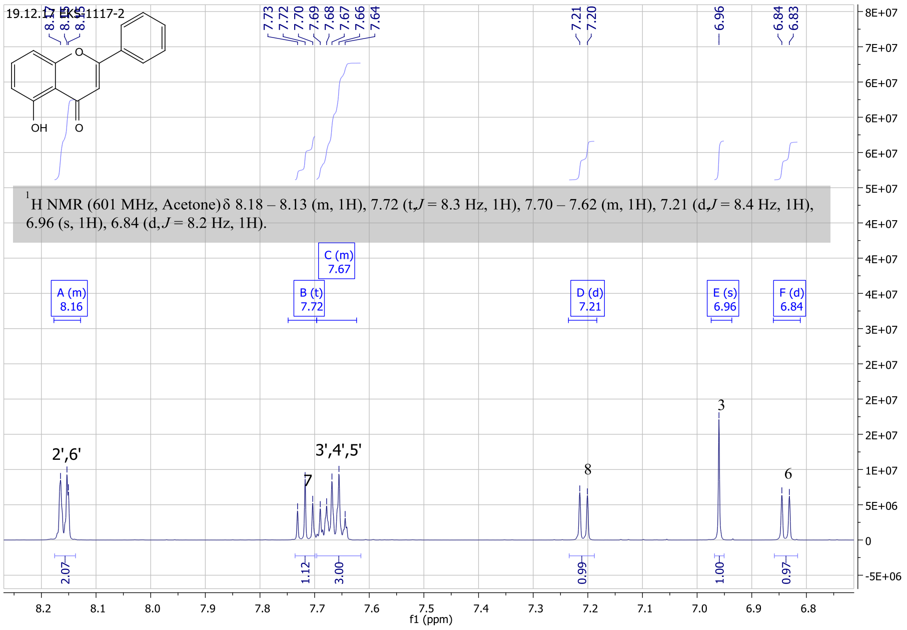

Figure S22. ^{13}C NMR spectrum of 5-hydroxyflavone (2) (Acetone-d$_6$, 151 MHz)

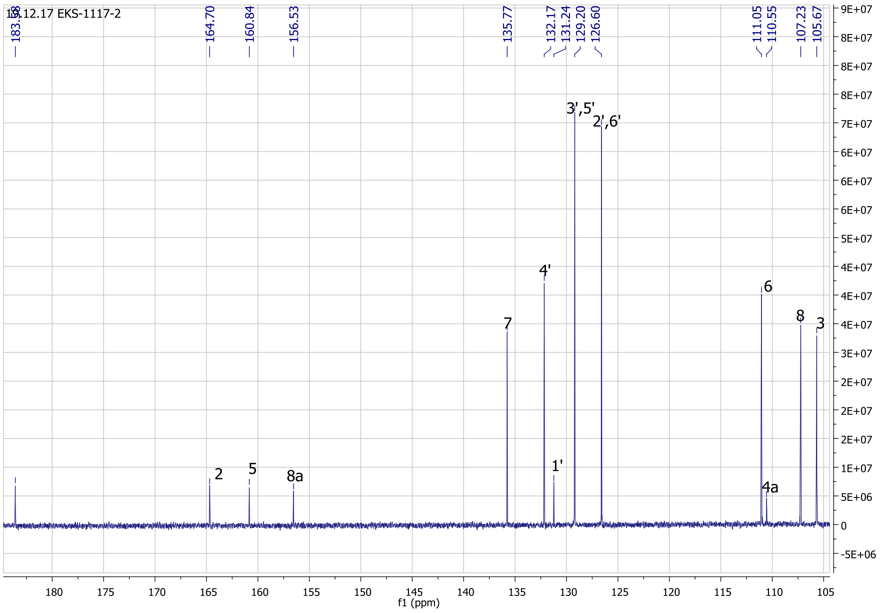

Figure S23. HSQC NMR spectrum of 5-hydroxyflavone (2) (Acetone-d₆, 151 MHz)

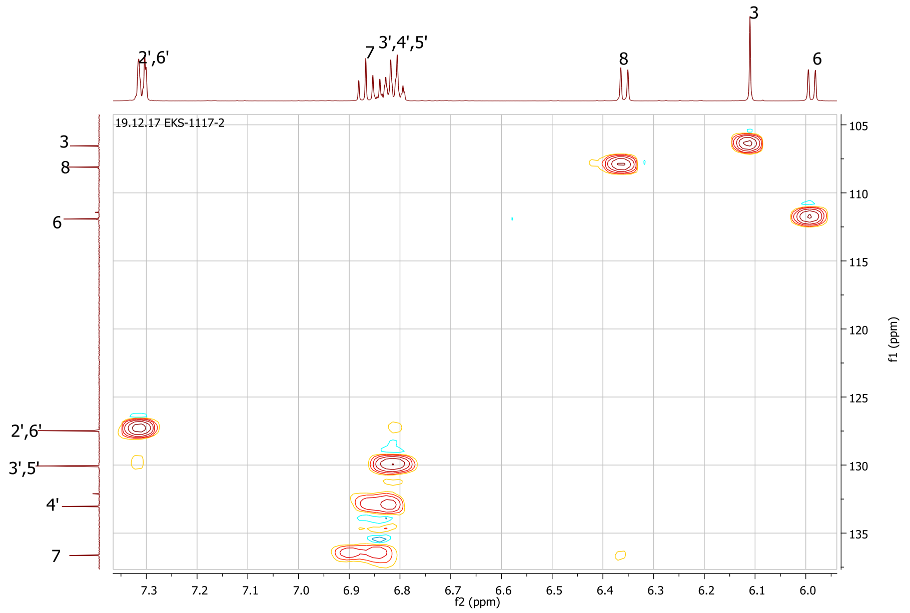

Figure S24. HMBC NMR spectrum of 5-hydroxyflavone (2) (Acetone-d₆, 151 MHz)

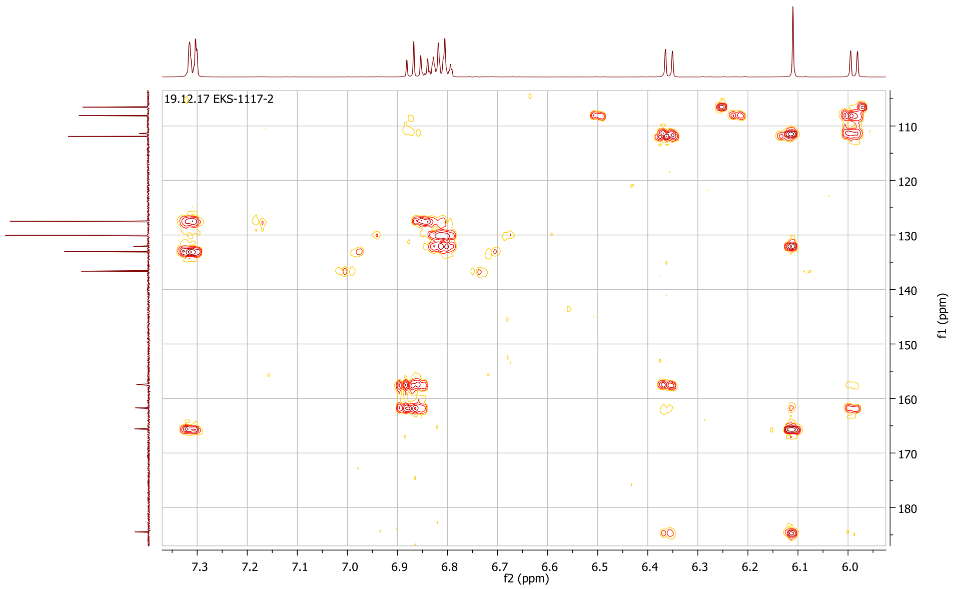

Figure S25. ^{1}H NMR spectrum of 5-hydroxyflavone 4′-*O*-*β*-D-(4″-*O*-methyl)-glucopyranoside (2a) (Acetone-d6, 600 MHz)

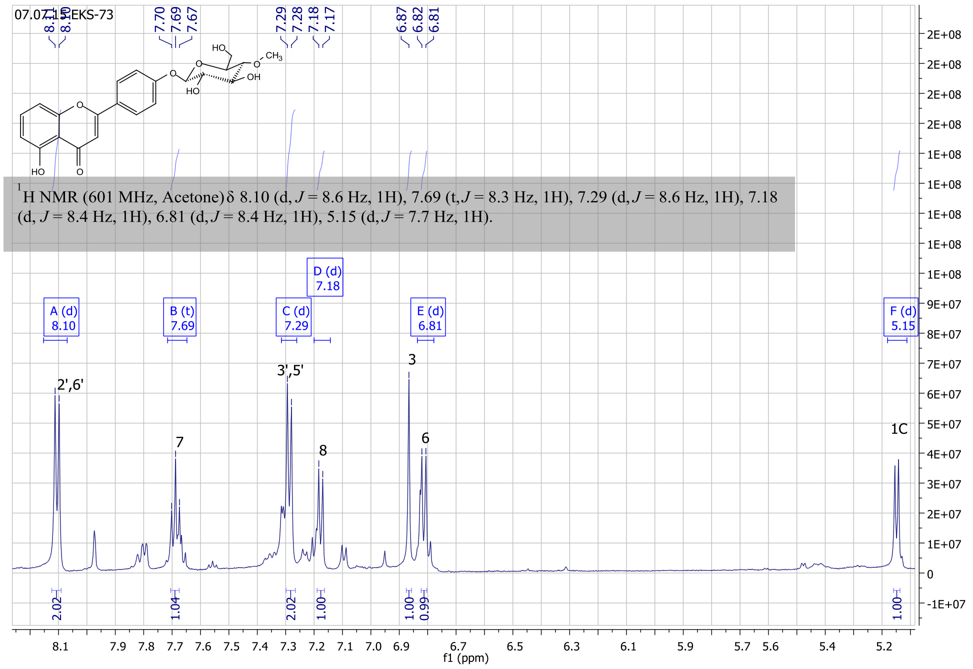

Figure S26. ^{1}H NMR spectrum of 5-hydroxyflavone 4′-*O*-*β*-D-(4″-*O*-methyl)-glucopyranoside (2a) (Acetone-d6, 600 MHz)

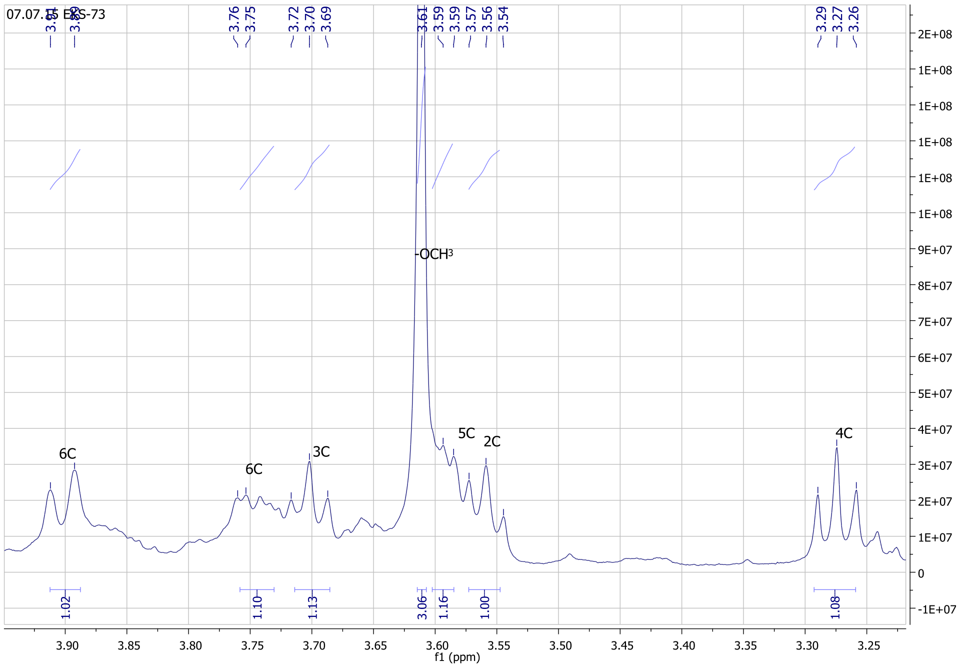

Figure S27. ^{13}C NMR spectrum of 5-hydroxyflavone 4′-*O*-*β*-D-(4″-*O*-methyl)-glucopyranoside (2a) (Acetone-d$_6$, 151 MHz)

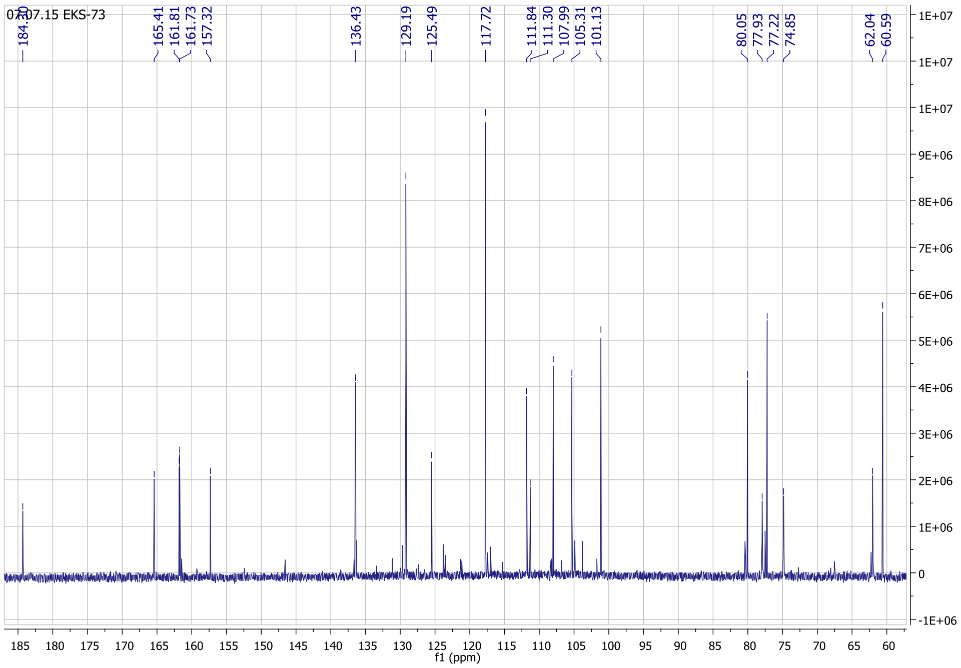

Figure S28. HSQC NMR spectrum of 5-hydroxyflavone 4′-*O*-*β*-D-(4″-*O*-methyl)-glucopyranoside (2a) (Acetone-d$_6$, 151 MHz)

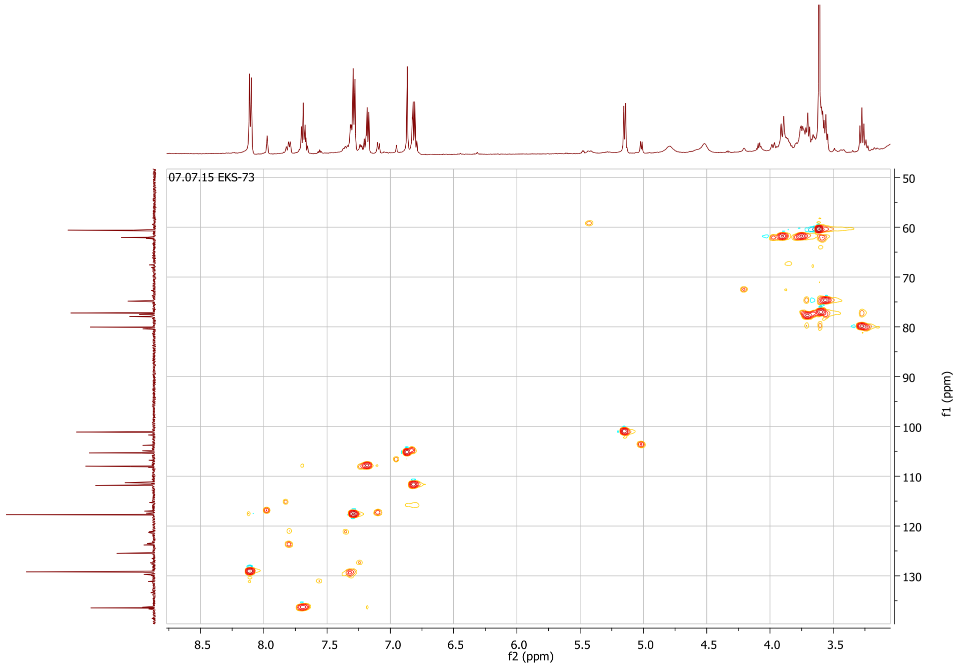

Figure S29. HMBC NMR spectrum of 5-hydroxyflavone 4′-*O*-*β*-D-(4″-*O*-methyl)-glucopyranoside (2a) (Acetone-d$_6$, 151 MHz)

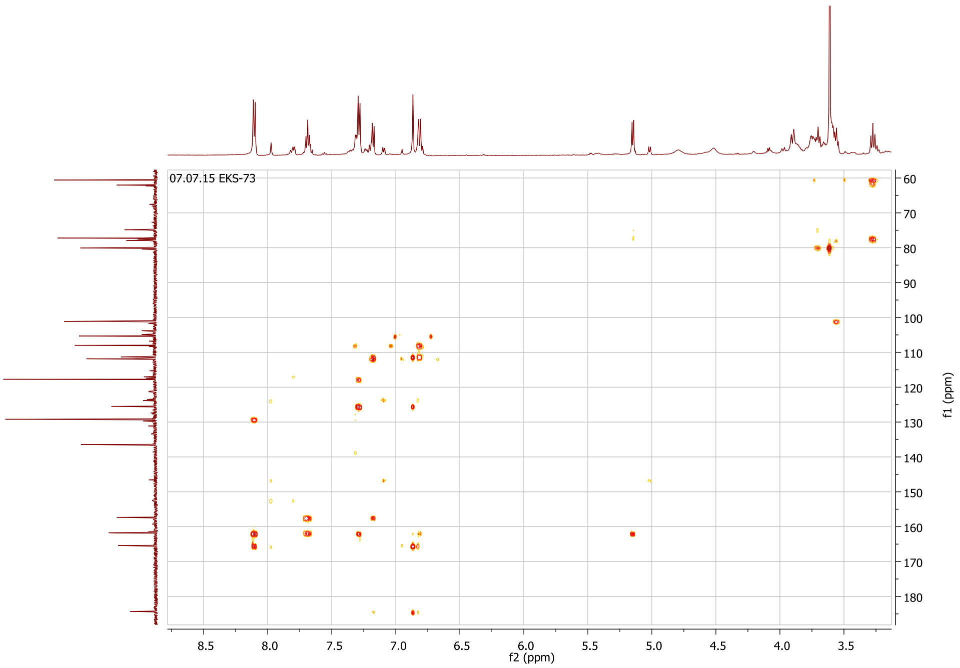

Figure S30. ^{1}H NMR spectrum of 6-hydroxyflavone (3) (Acetone-d$_6$, 600 MHz)

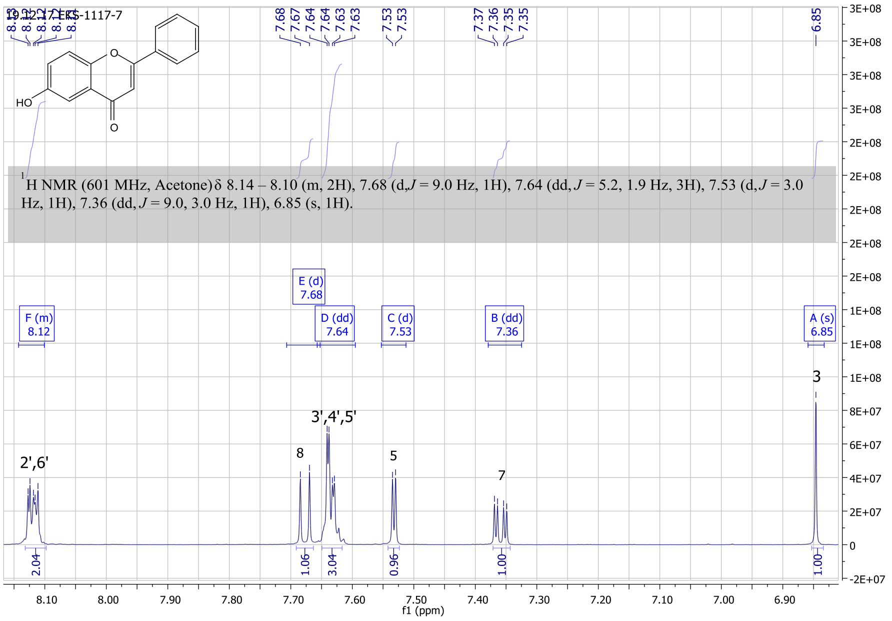

Figure S31. ^{13}C NMR spectrum of 6-hydroxyflavone (3) (Acetone-d_6, 151 MHz)

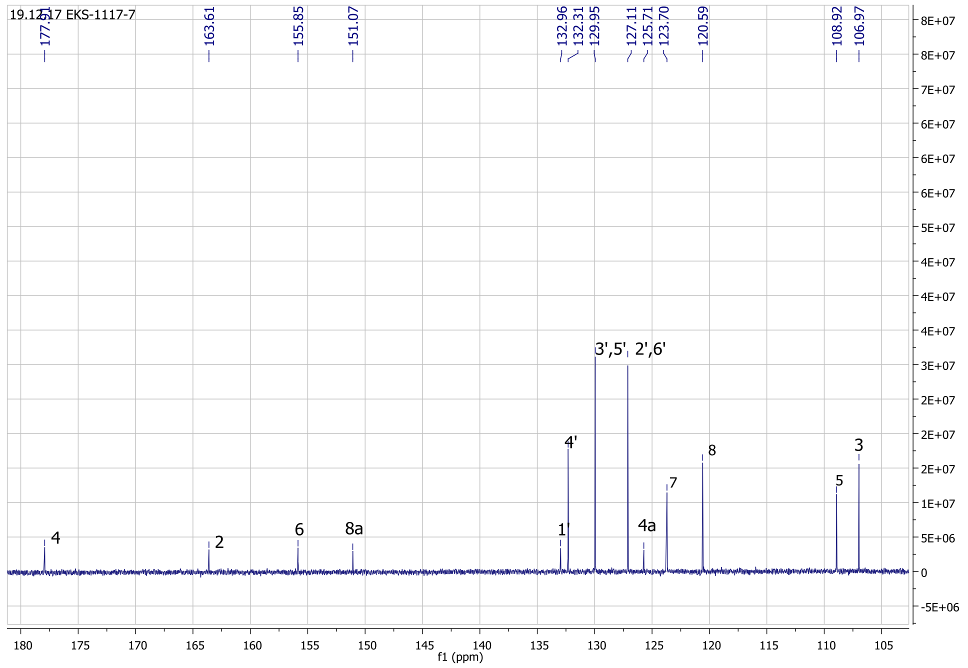

Figure S32. HSQC NMR spectrum of 6-hydroxyflavone (3) (Acetone-d_6, 151 MHz)

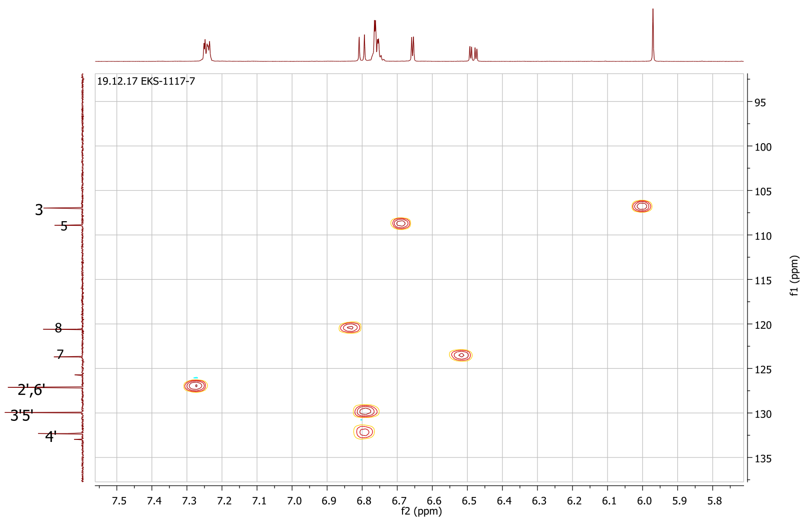

Figure S33. HMBC NMR spectrum of 6-hydroxyflavone (3) (Acetone-d_6, 151 MHz)

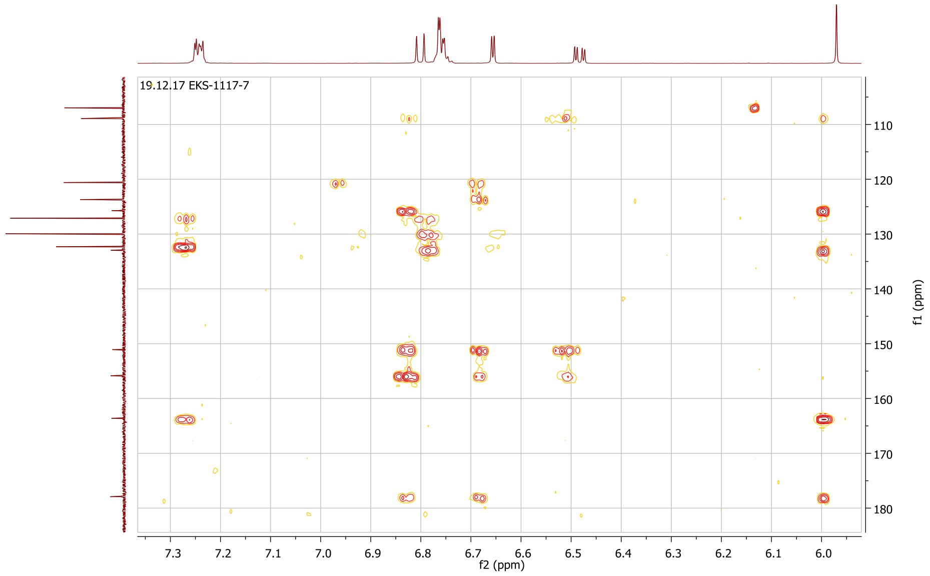

Figure S34. ^{1}H NMR spectrum of flavone 6-*O*-*β*-D-(4′′-*O*-methyl)-glucopyranoside (3a) (Acetone-d_6, 600 MHz)

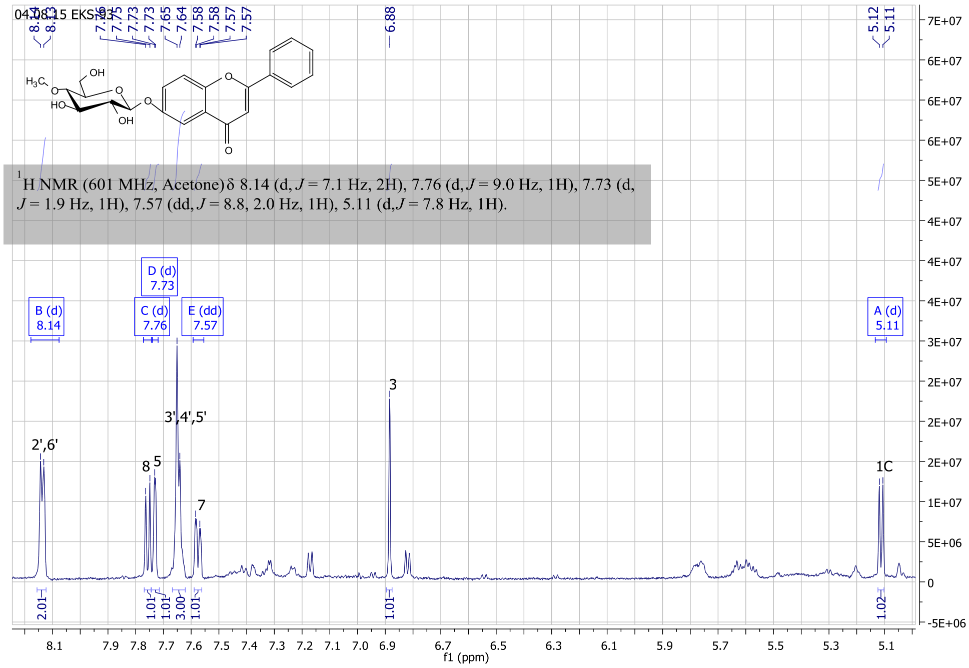

Figure S35. ^{1}H NMR spectrum of flavone 6-*O*-*β*-D-(4″-*O*-methyl)-glucopyranoside (3a) (Acetone-d6, 600 MHz)

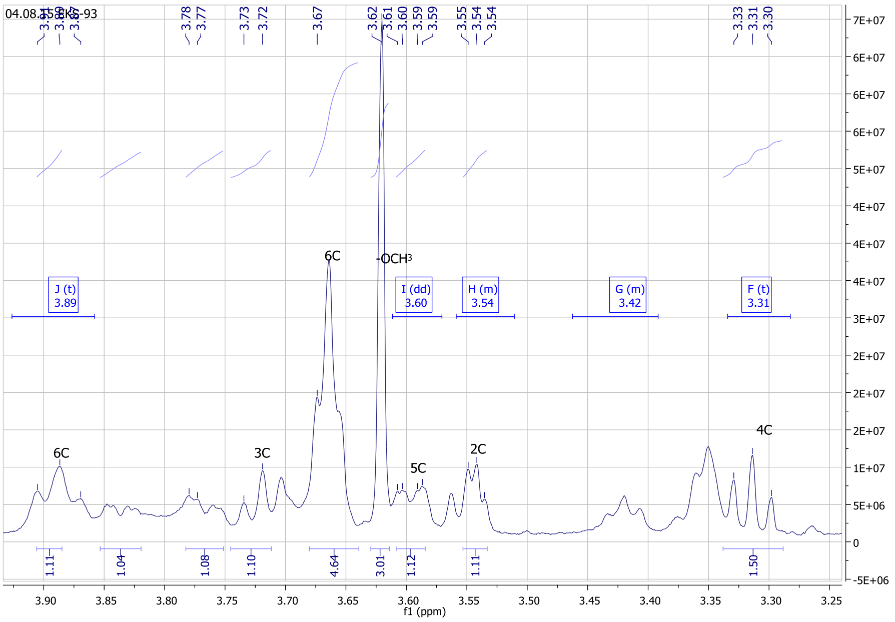

Figure S36. ^{13}C NMR spectrum of flavone 6-*O*-*β*-D-(4″-*O*-methyl)-glucopyranoside (3a) (Acetone-d6, 151 MHz)

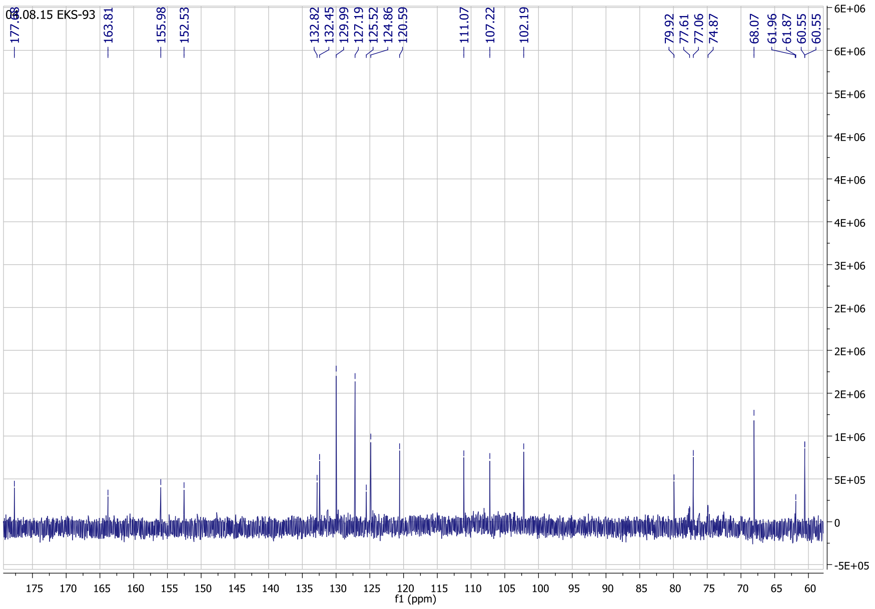

Figure S37. HSQC NMR spectrum of flavone 6-*O*-*β*-D-(4″-*O*-methyl)-glucopyranoside (3a) (Acetone-d6, 151 MHz)

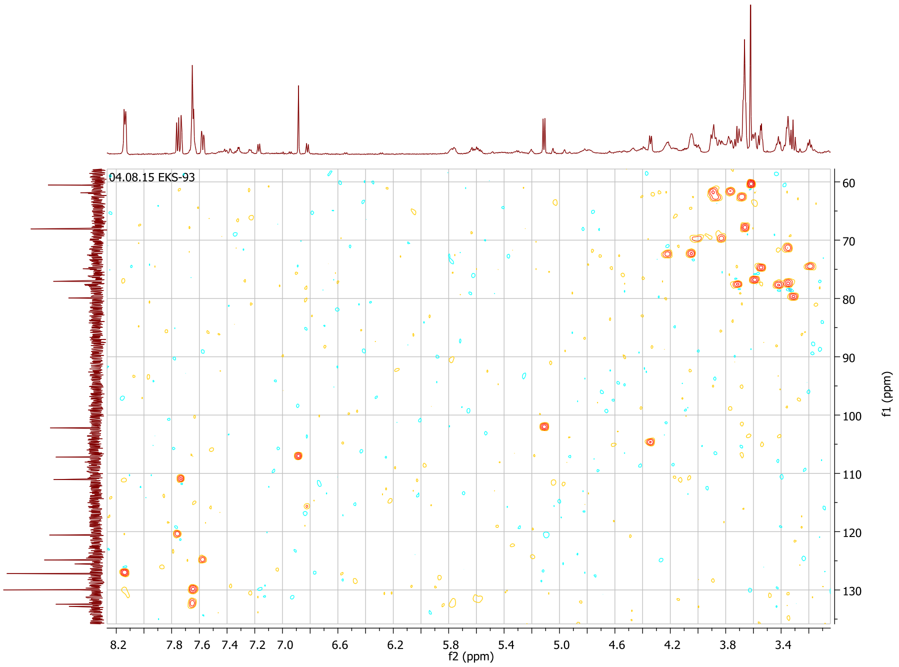

Figure S38. HMBC NMR spectrum of flavone 6-*O*-*β*-D-(4″-*O*-methyl)-glucopyranoside (3a) (Acetone-d6, 151 MHz)

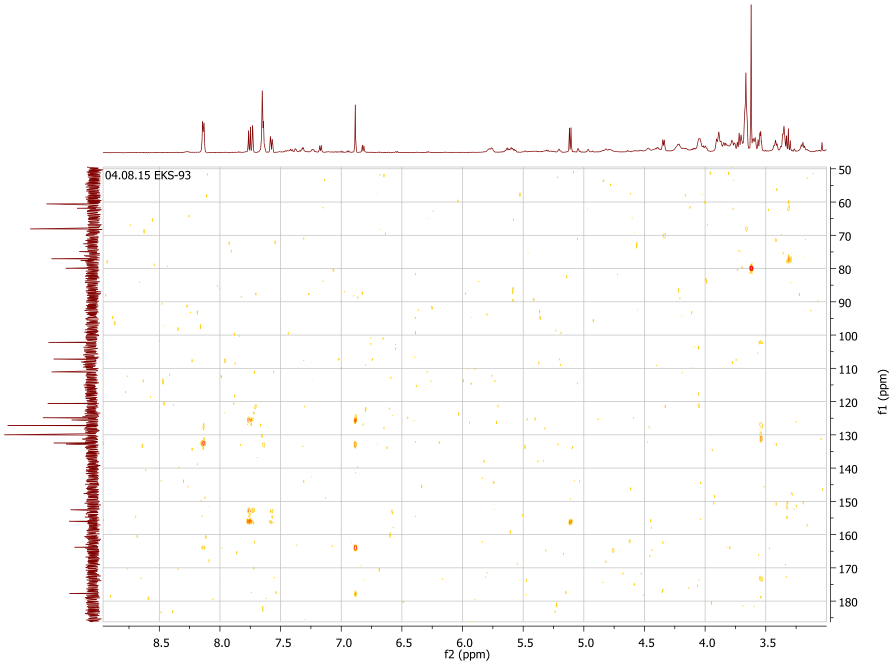

Figure S39. ^{1}H NMR spectrum of 7-hydroxyflavone (4) (Acetone-d$_6$, 600 MHz)

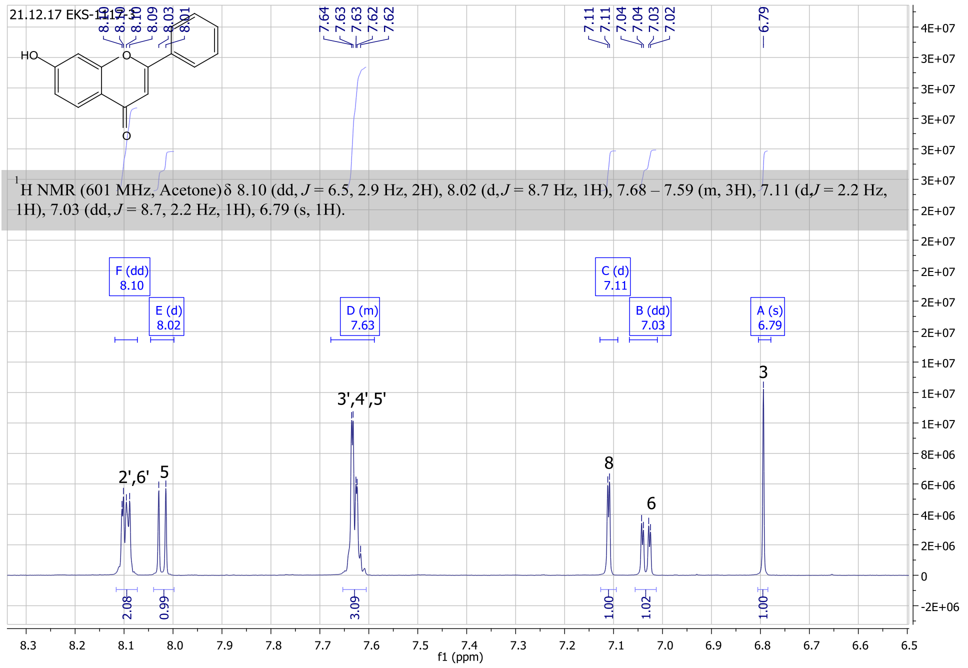

Figure S40. ^{13}C NMR spectrum of 7-hydroxyflavone (4) (Acetone-d$_6$, 151 MHz)

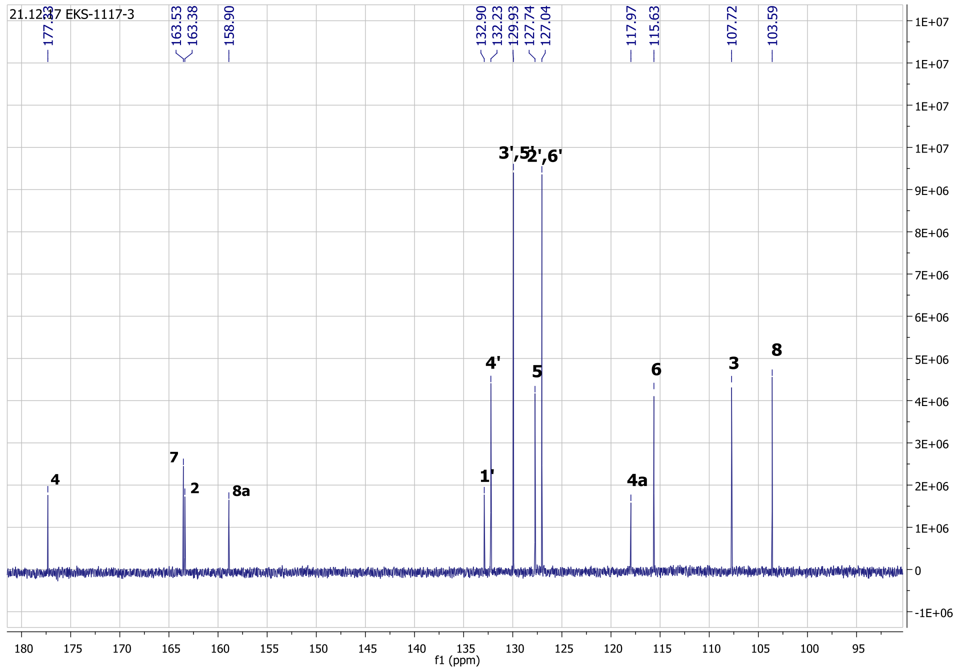

Figure S41. HSQC NMR spectrum of 7-hydroxyflavone (4) (Acetone-d_6, 151 MHz)

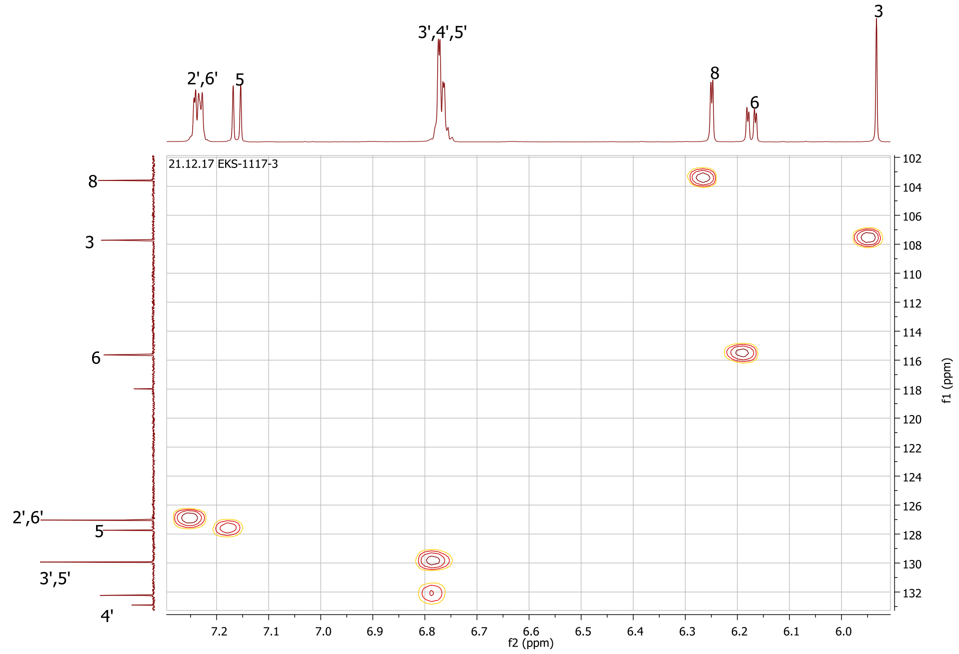

Figure S42. HMBC NMR spectrum of 7-hydroxyflavone (4) (Acetone-d_6, 151 MHz)

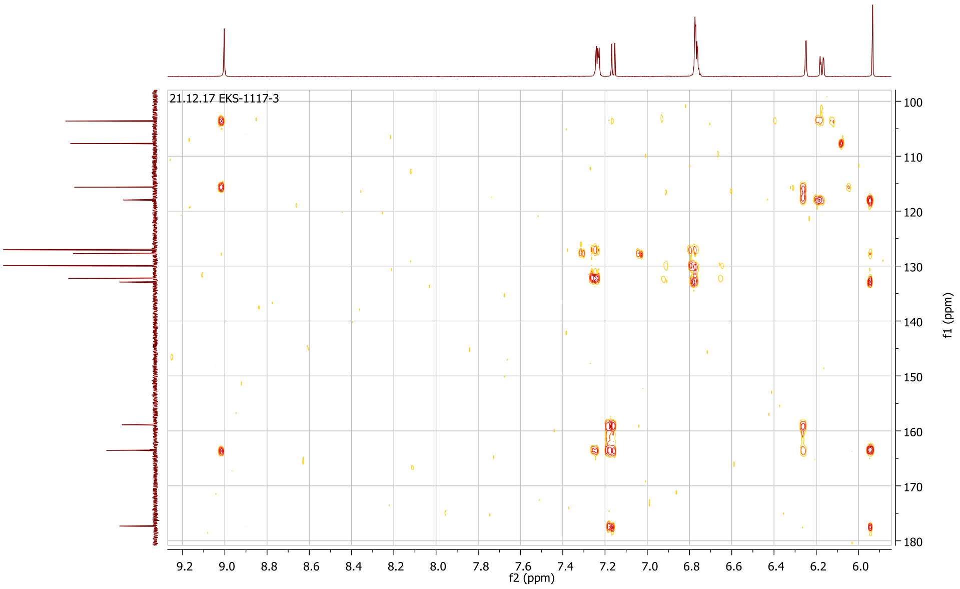

Figure S43. ^{1}H NMR spectrum of flavone 7-*O*-*β*-D-(4″-*O*-methyl)-glucopyranoside (4a) (Acetone-d_6, 600 MHz)

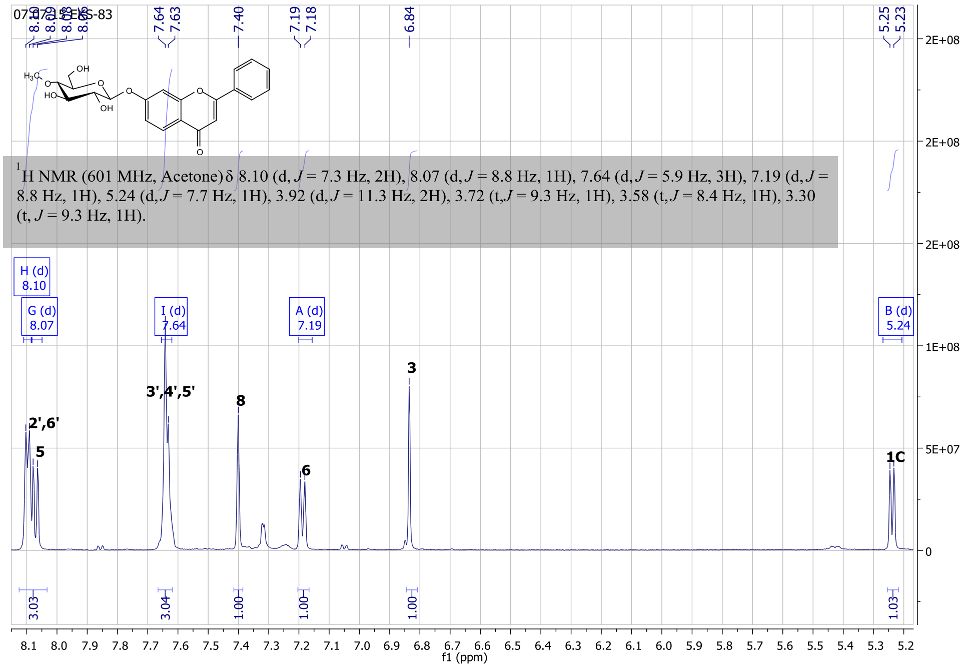

Figure S44. ^{1}H NMR spectrum of flavone 7-*O*-*β*-D-(4″-*O*-methyl)-glucopyranoside (4a) (Acetone-d_6, 600 MHz)

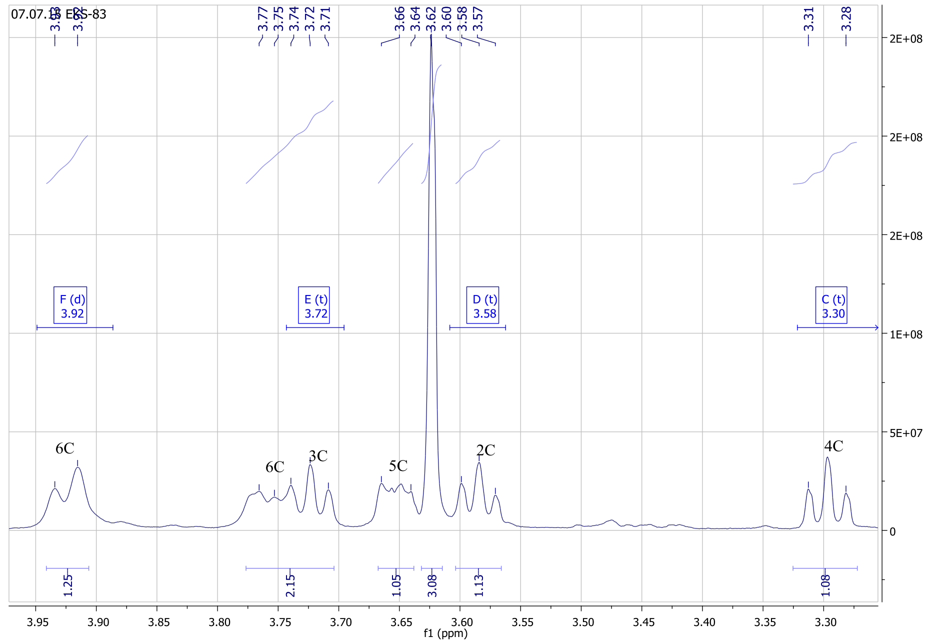

Figure S45. ^{13}C NMR spectrum of flavone 7-*O*-*β*-D-(4″-*O*-methyl)-glucopyranoside (4a) (Acetone-d$_6$, 151 MHz)

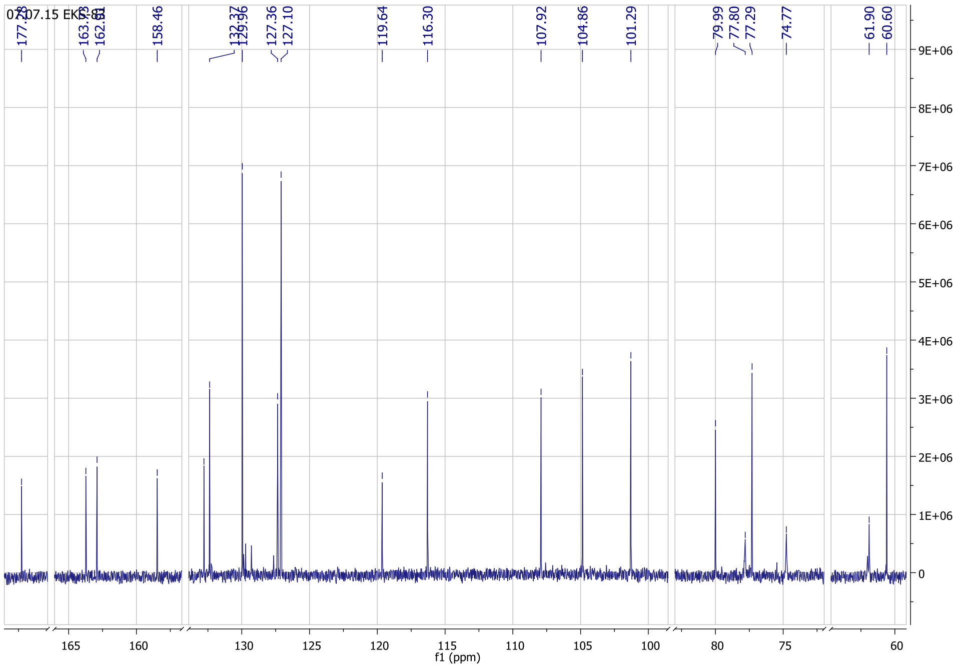

Figure S46. HSQC NMR spectrum of flavone 7-*O*-*β*-D-(4″-*O*-methyl)-glucopyranoside (4a) (Acetone-d$_6$, 151 MHz)

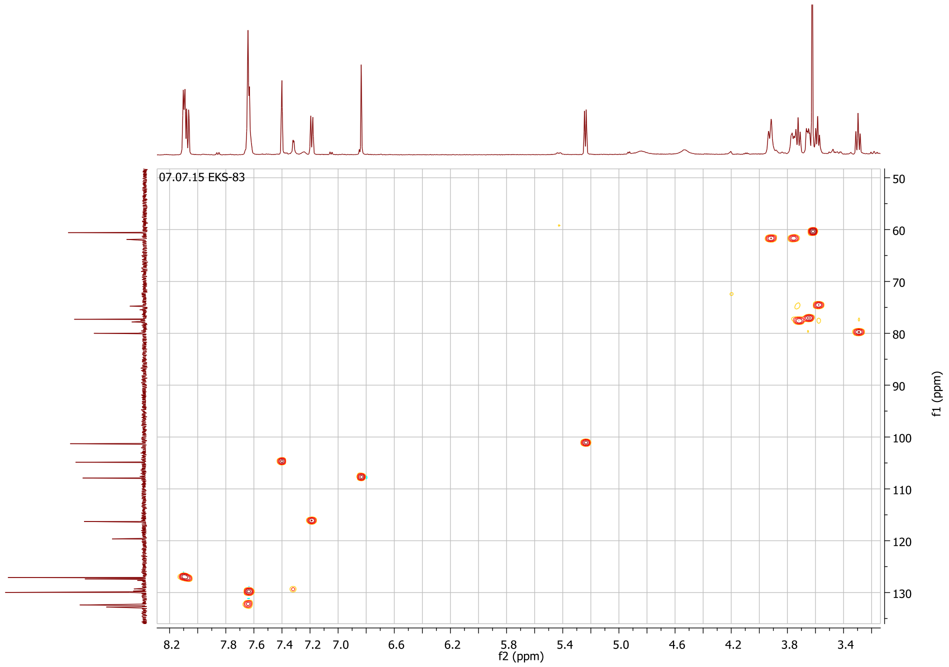

Figure S47. HMBC NMR spectrum of flavone 7-*O*-*β*-D-(4″-*O*-methyl)-glucopyranoside (4a) (Acetone-d6, 151 MHz)

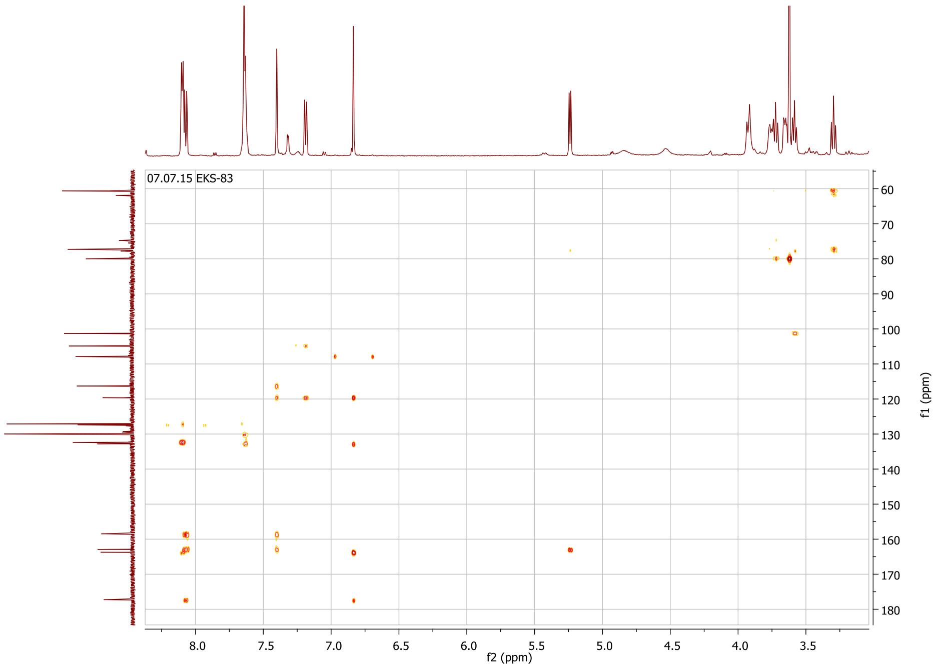

Figure S48. ^{1}H NMR spectrum of 4′,7-dihydroxyisoflavone (5) (Acetone-d6, 600 MHz)

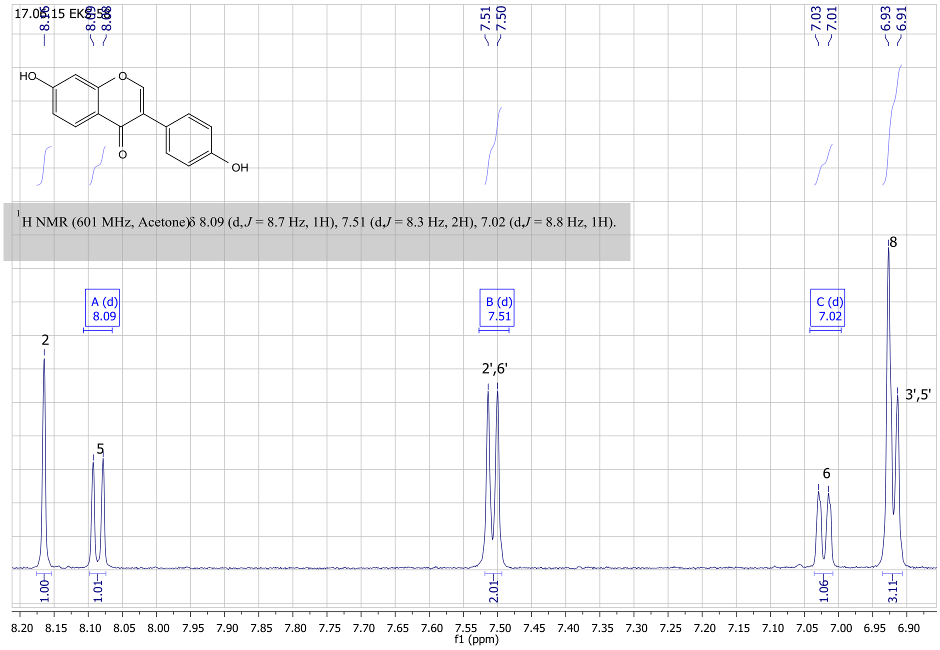

Figure S49. ^{13}C NMR spectrum of 4′,7-dihydroxyisoflavone (5) (Acetone-d$_6$, 151 MHz)

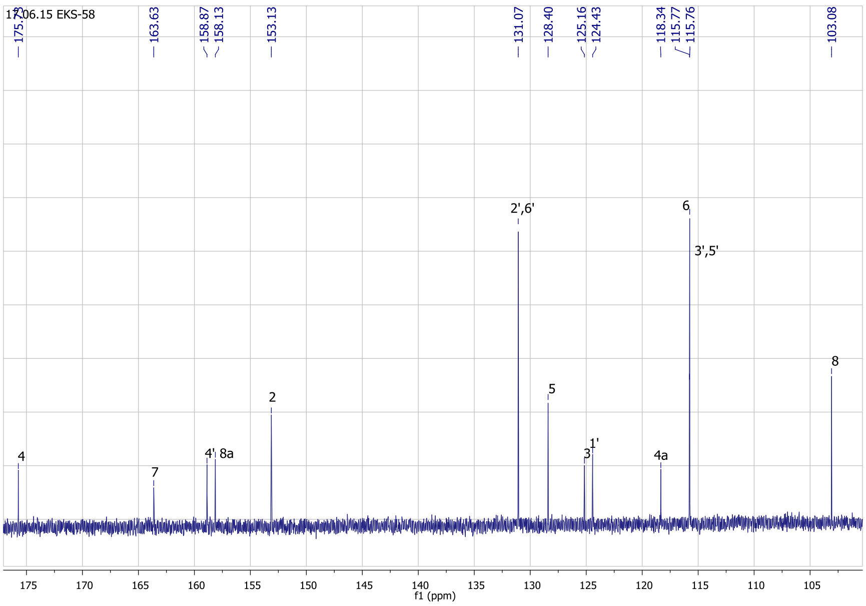

Figure S50. HSQC NMR spectrum of 4′,7-dihydroxyisoflavone (5) (Acetone-d$_6$, 151 MHz)

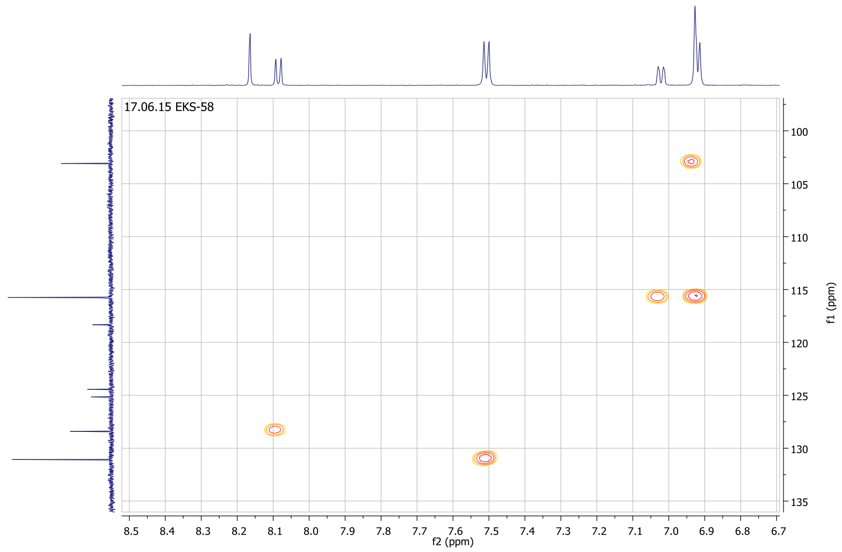

Figure S51. HMBC NMR spectrum of 4′,7-dihydroxyisoflavone (5) (Acetone-d$_6$, 151 MHz)

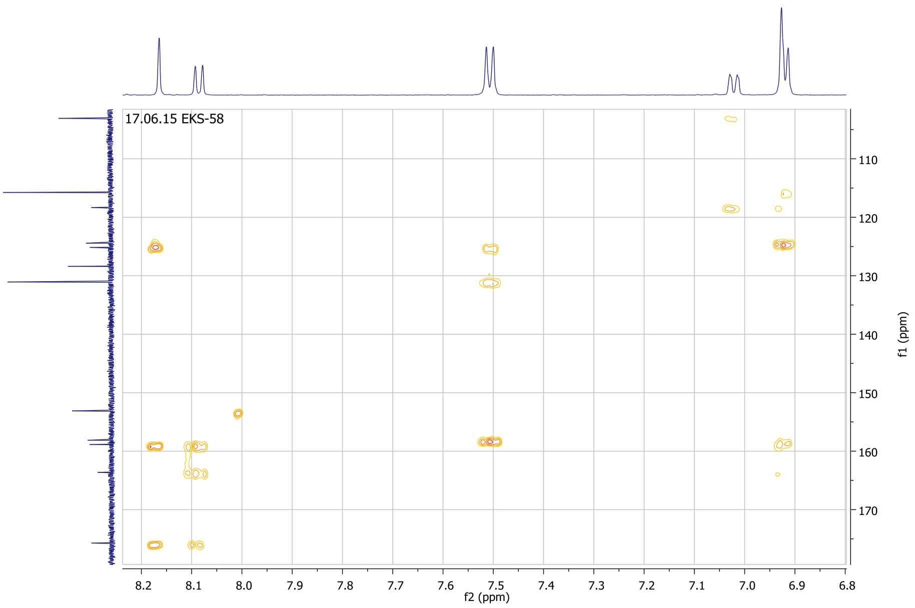

Figure S52. ^{1}H NMR spectrum of 4′-hydroxyisoflavone 7-*O*-*β*-D-(4″-*O*-methyl)-glucopyranoside (5a) (Acetone-d$_6$, 600 MHz)

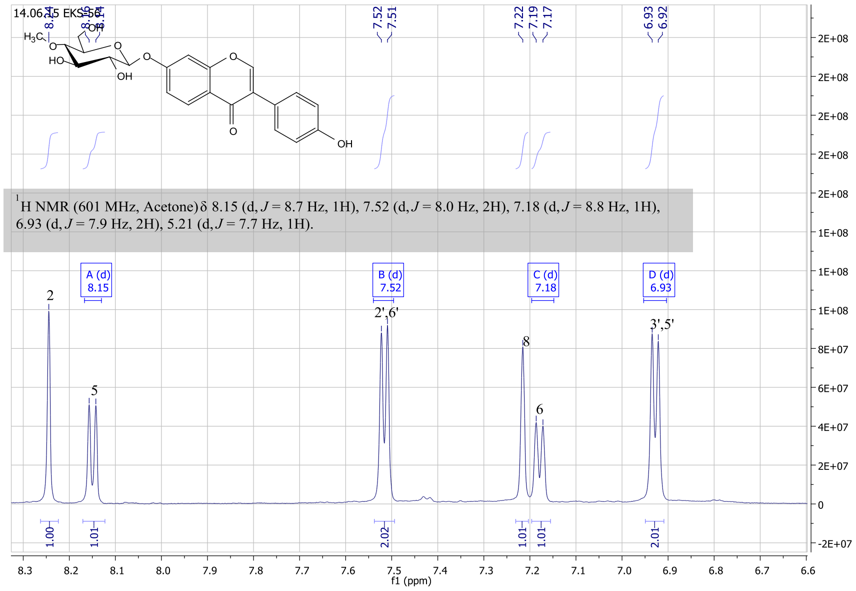

Figure S53. ^{1}H NMR spectrum of 4′-hydroxyisoflavone 7-*O*-*β*-D-(4″-*O*-methyl)-glucopyranoside (5a) (Acetone-d$_6$, 600 MHz)

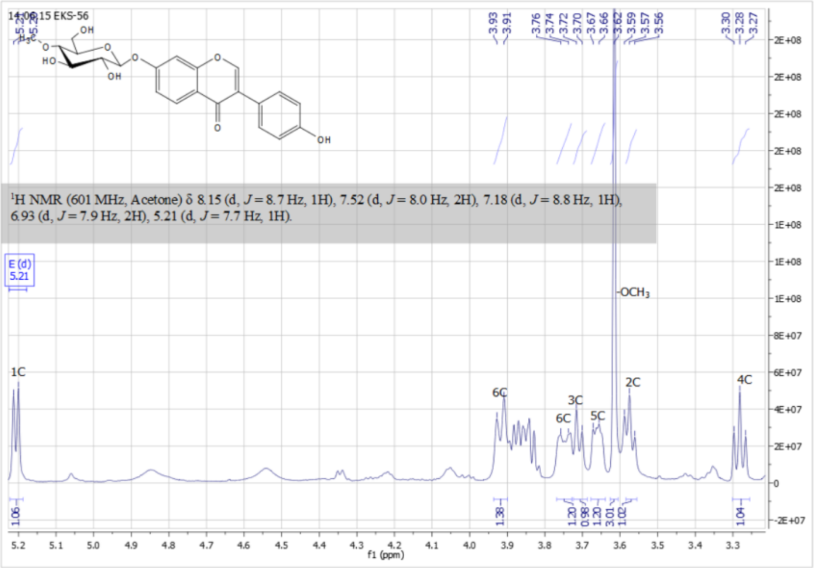

Figure S54. ^{13}C NMR spectrum of 4′-hydroxyisoflavone 7-*O*-*β*-D-(4″-*O*-methyl)-glucopyranoside (5a)(Acetone-d$_6$, 151 MHz)

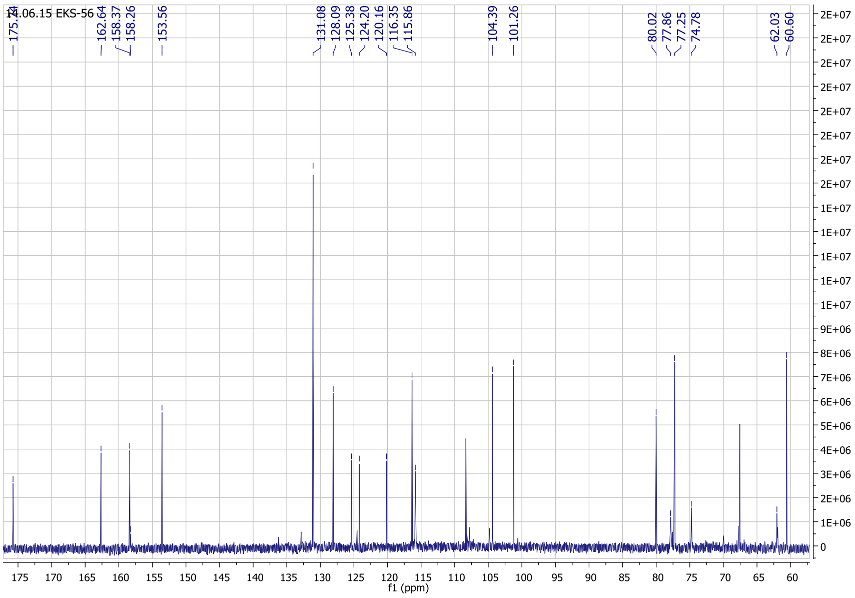

Figure S55. HSQC NMR spectrum of 4′-hydroxyisoflavone 7-*O*-*β*-D-(4″-*O*-methyl)-glucopyranoside (5a) (Acetone-d$_6$, 151 MHz)

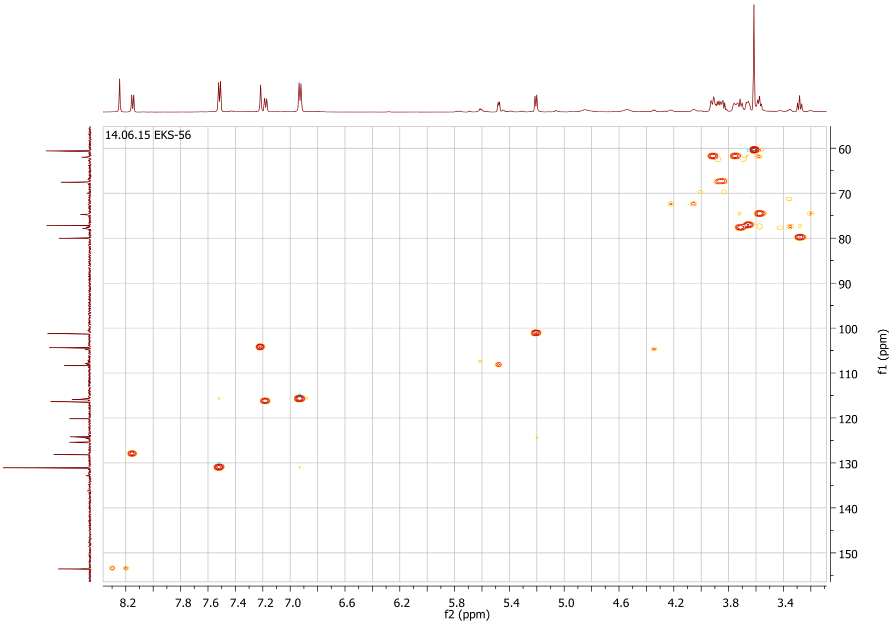

Figure S56. HMBC NMR spectrum of 4′-hydroxyisoflavone 7-*O*-*β*-D-(4″-*O*-methyl)-glucopyranoside (5a) (Acetone-d$_6$, 151 MHz)

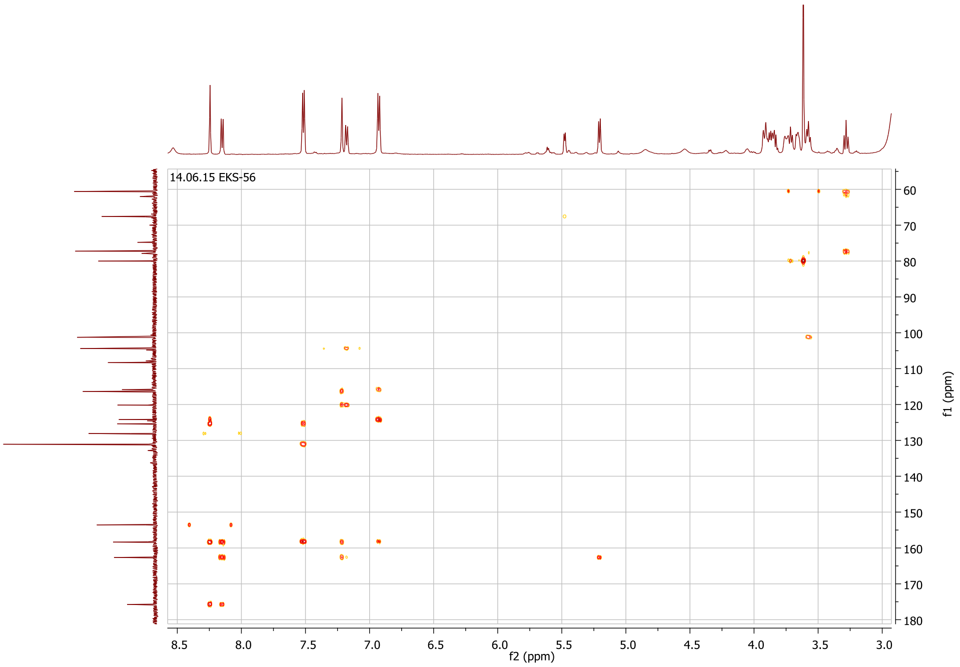

Figure S57. ^{1}H NMR spectrum of 7-aminoflavone (6) (Acetone-d_6, 600 MHz)

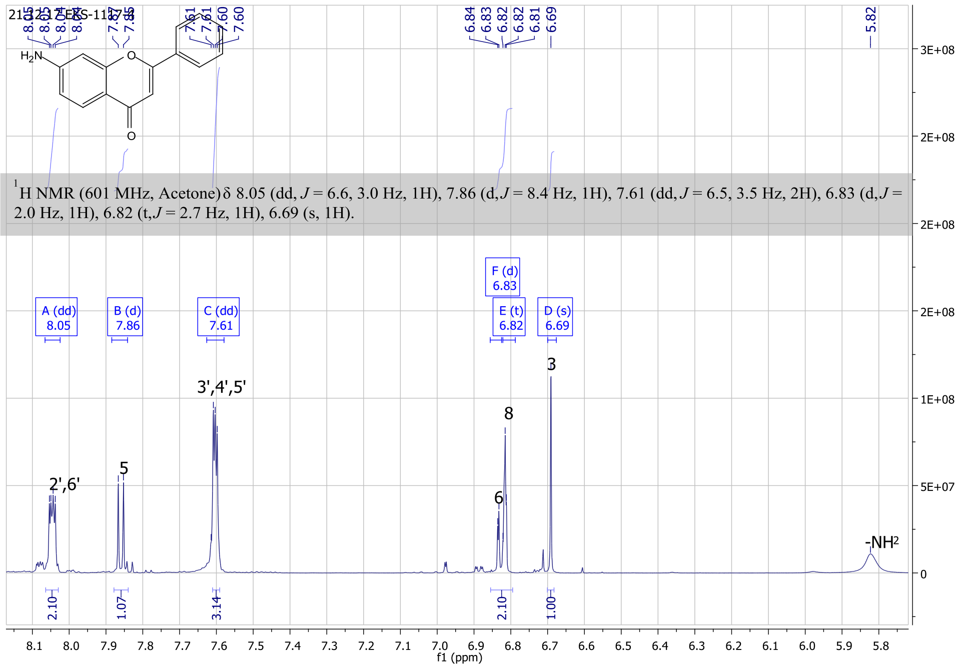

Figure S58. ^{13}C NMR spectrum of 7-aminoflavone (6) (Acetone-d_6, 151 MHz)

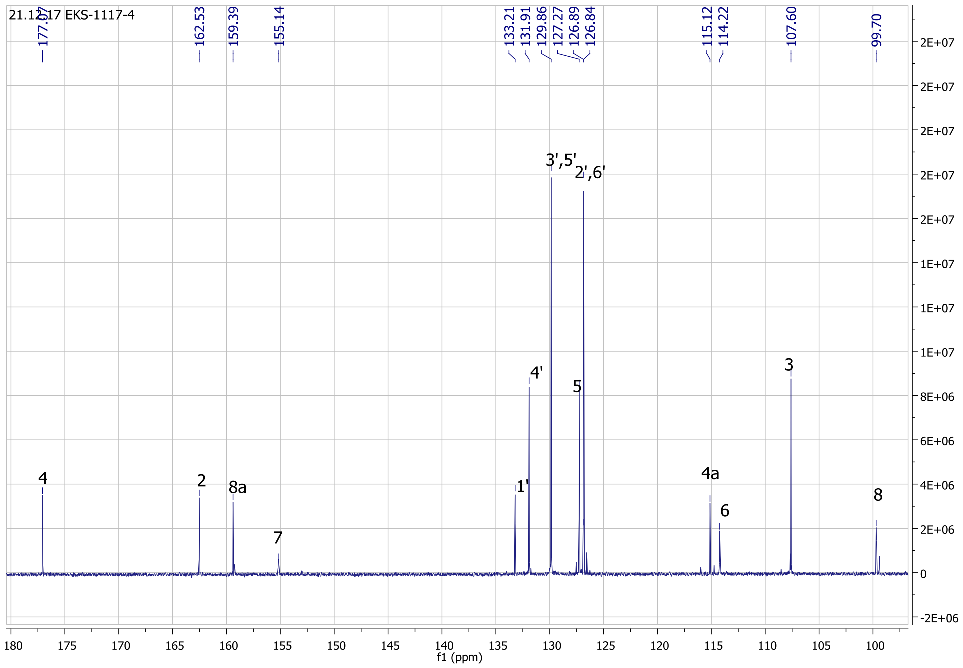

Figure S59. HSQC NMR spectrum of 7-aminoflavone (6) (Acetone-d₆, 151 MHz)

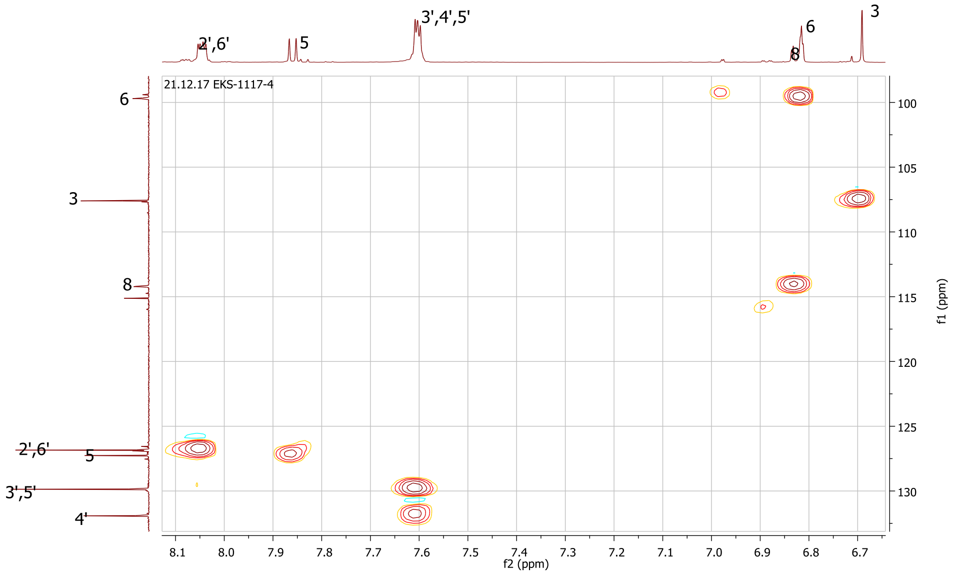

Figure S60. HMBC NMR spectrum of 7-aminoflavone (6) (Acetone-d₆, 151 MHz)

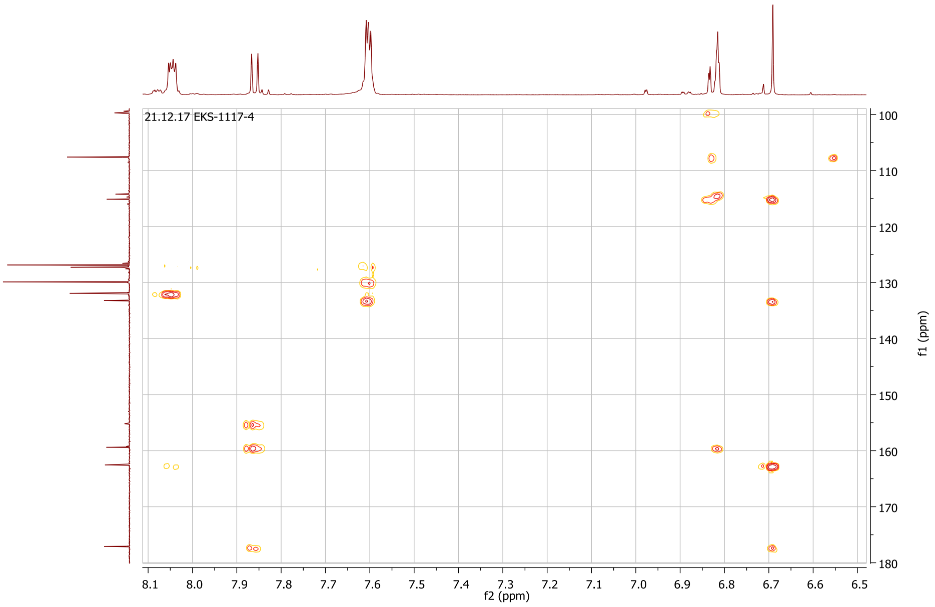

Figure S61. ^{1}H NMR spectrum of 7-acetamidoflavone (6a) (Acetone-d$_6$, 600 MHz)

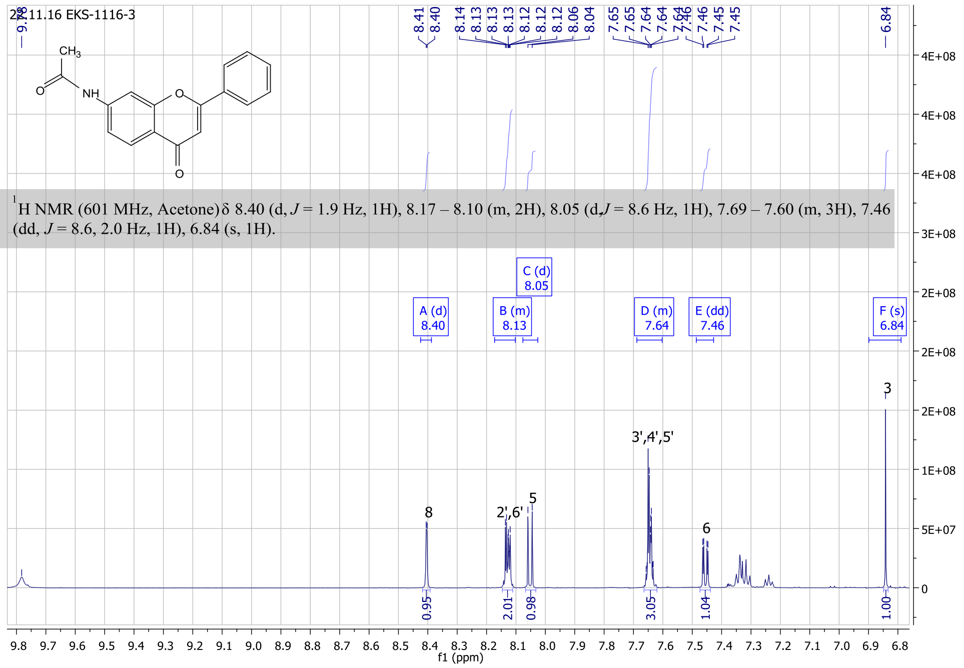

Figure S62. ^{13}C NMR spectrum of 7-acetamidoflavone (6a) (Acetone-d$_6$, 151 MHz)

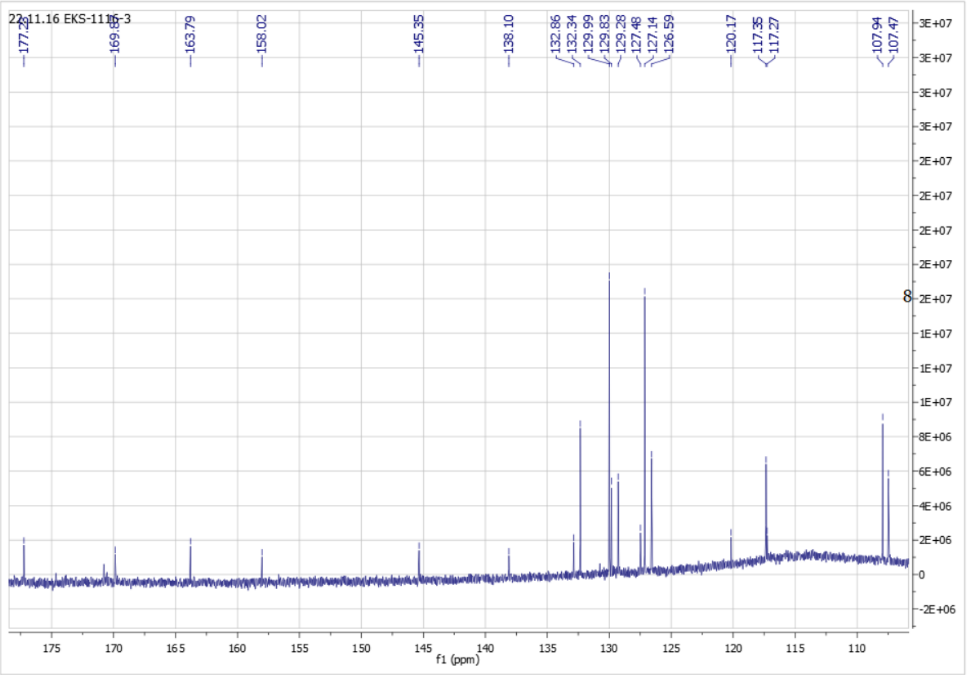

Figure S63. HSQC NMR spectrum of 7-acetamidoflavone (6a) (Acetone-d6, 151 MHz)

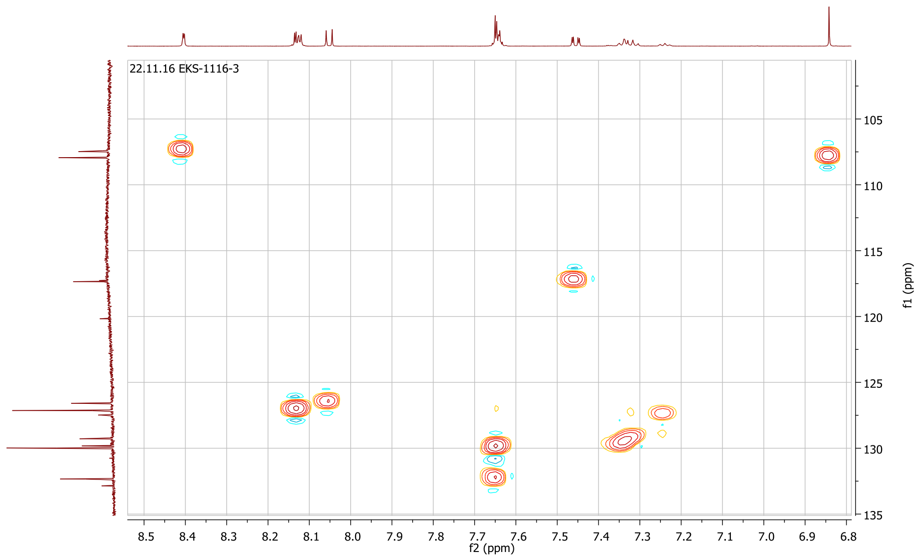

Figure S64. HMBC NMR spectrum of 7-acetamidoflavone (6a) (Acetone-d6, 151 MHz)

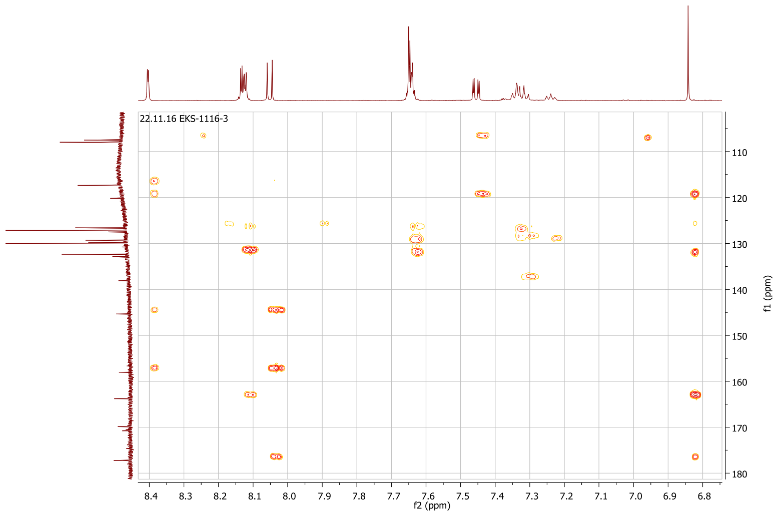

Figure S65. ^{1}H NMR spectrum of 4′-hydroxy-7-acetamidoflavone (6b) (Acetone-d_6, 600 MHz)

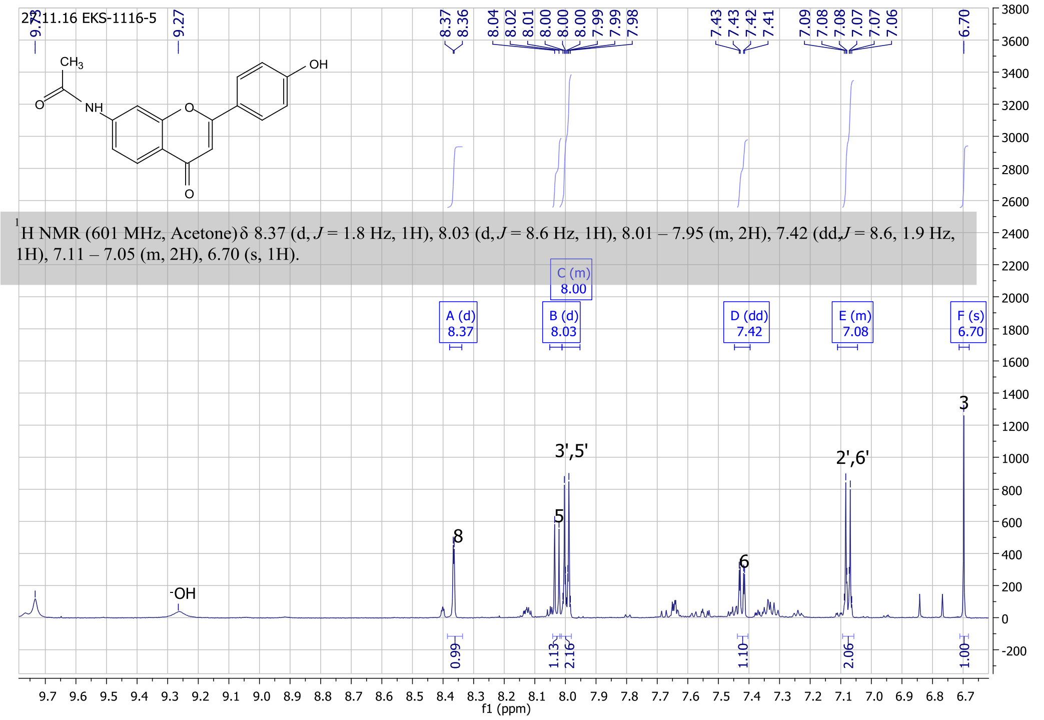

Figure S66. ^{13}C NMR spectrum of 4′-hydroxy-7-acetamidoflavone (6b) (Acetone-d_6, 151 MHz)

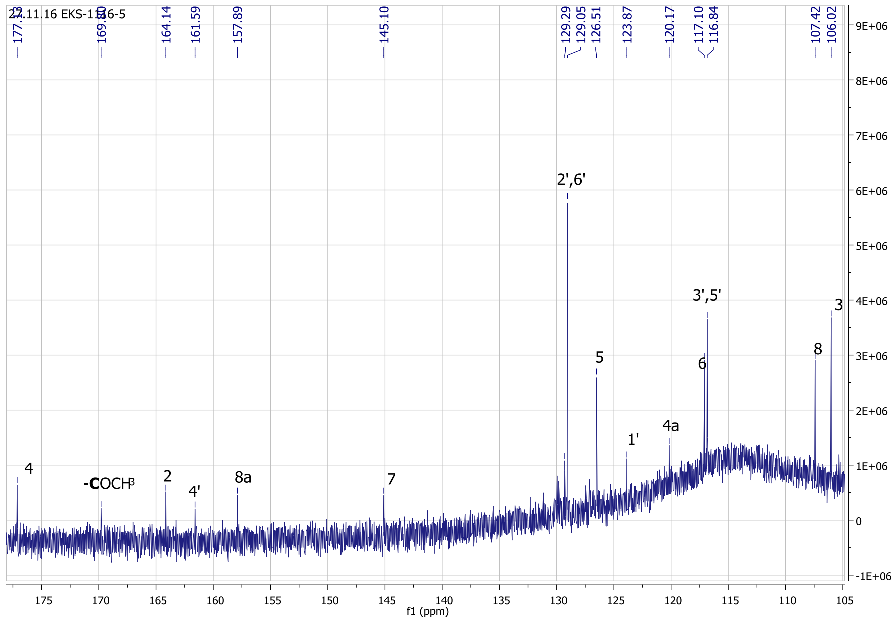

Figure S67. HSQC NMR spectrum of 4′-hydroxy-7-acetamidoflavone (6b) (Acetone-d6, 151 MHz)

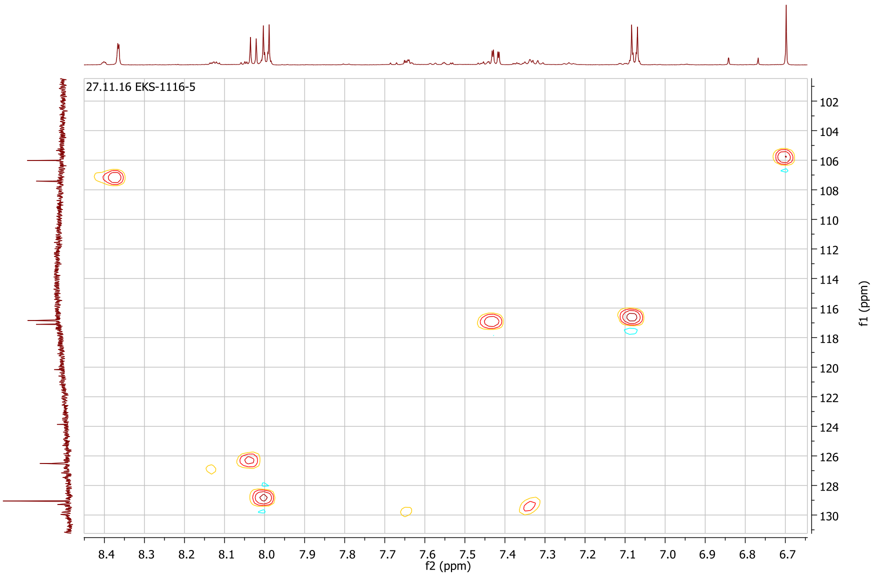

Figure S68. HMBC NMR spectrum of 4′-hydroxy-7-acetamidoflavone (6b) (Acetone-d6, 151 MHz)

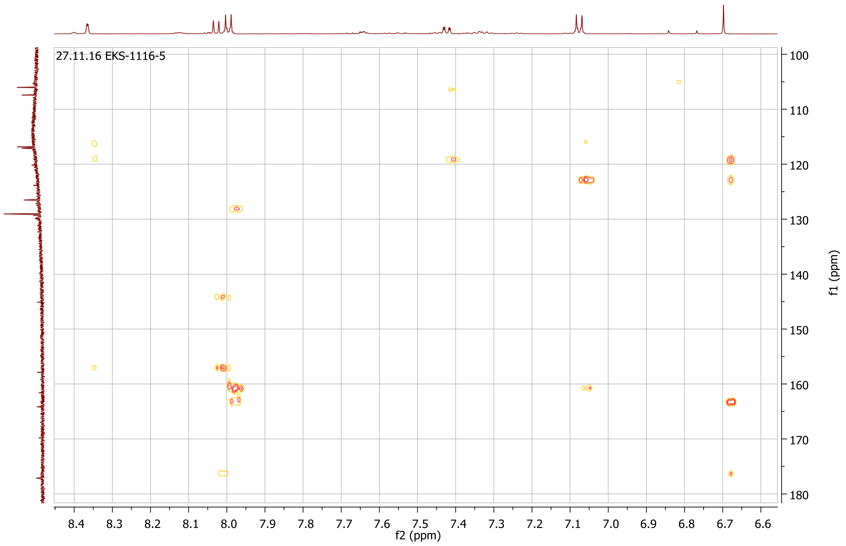

Figure S69. HPLC chromatogram of flavone (1)

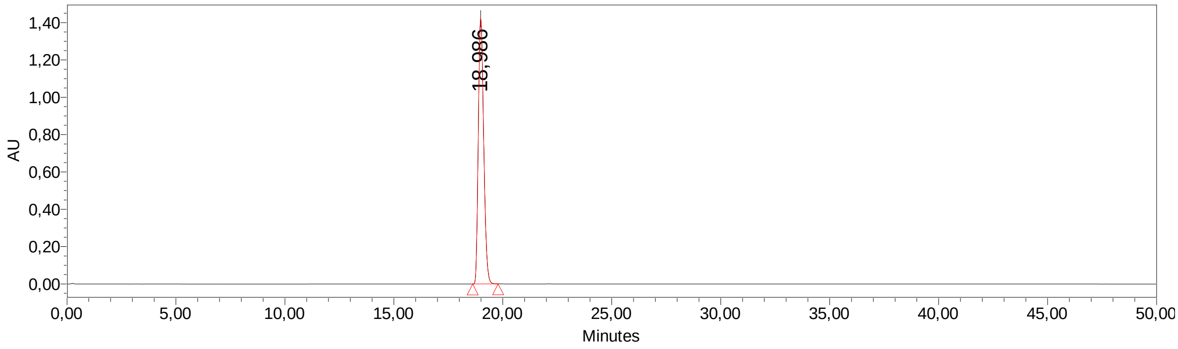

Figure S70. UV maxima of flavone (1) obtained after HPLC analysis

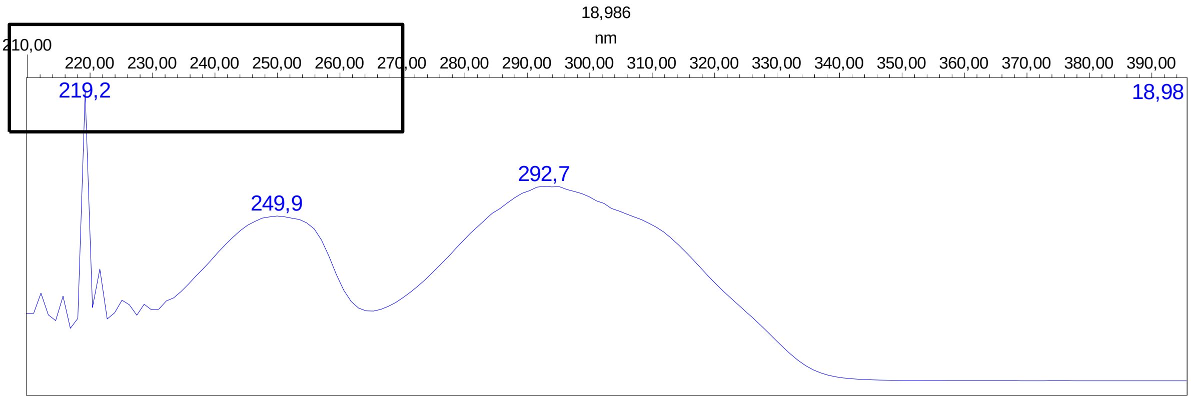

Figure S71. HPLC chromatogram of flavone 2′-*O*-*β*-D-(4″-*O*-methyl)-glucopyranoside (1a)

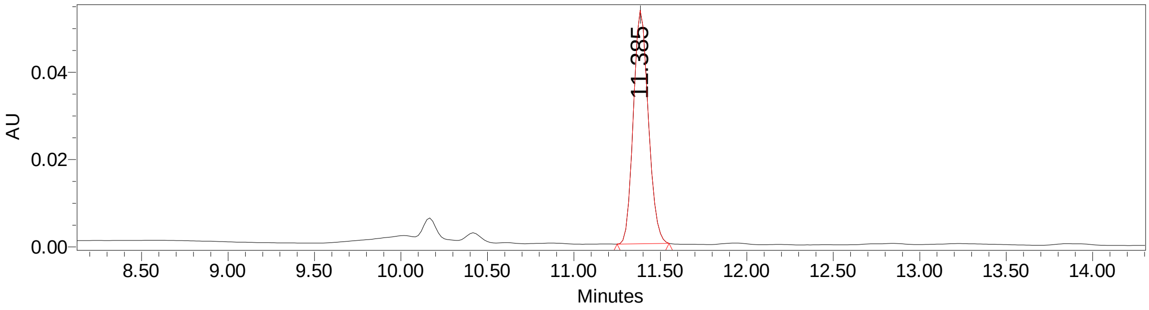

Figure S72. UV maxima of flavone 2′-*O*-*β*-D-(4″-*O*-methyl)-glucopyranoside (1a) obtained after HPLC analysis

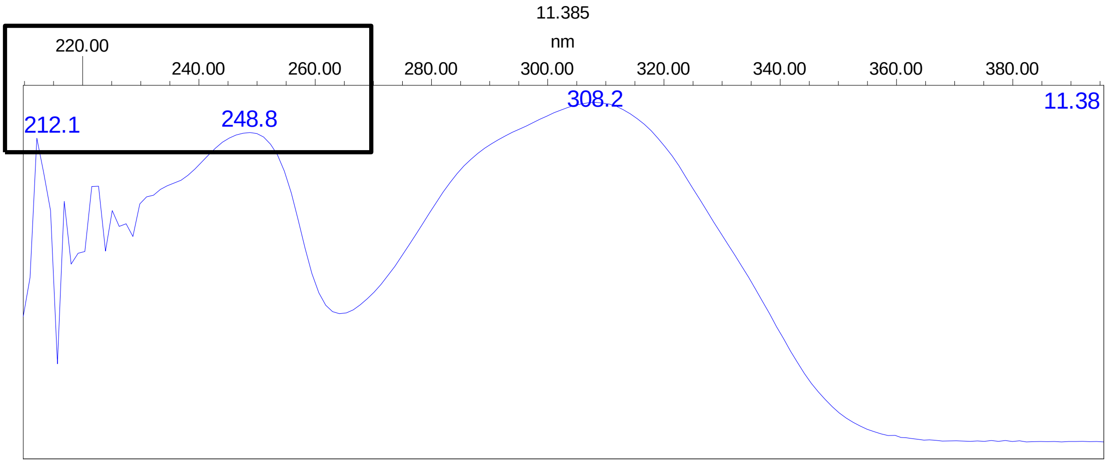

Figure S73. HPLC chromatogram of flavone 4′-*O*-*β*-D-(4″-*O*-methyl)-glucopyranoside (1b)

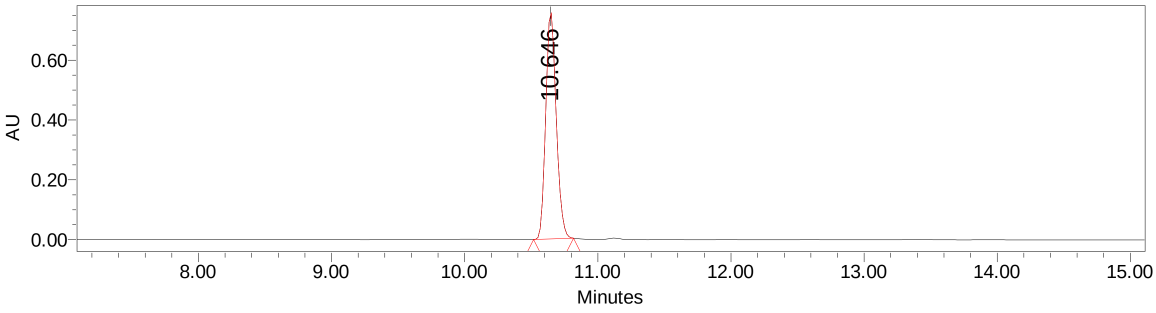

Figure S74. UV maxima of flavone 4′-*O*-*β*-D-(4″-*O*-methyl)-glucopyranoside (1b) obtained after HPLC analysis

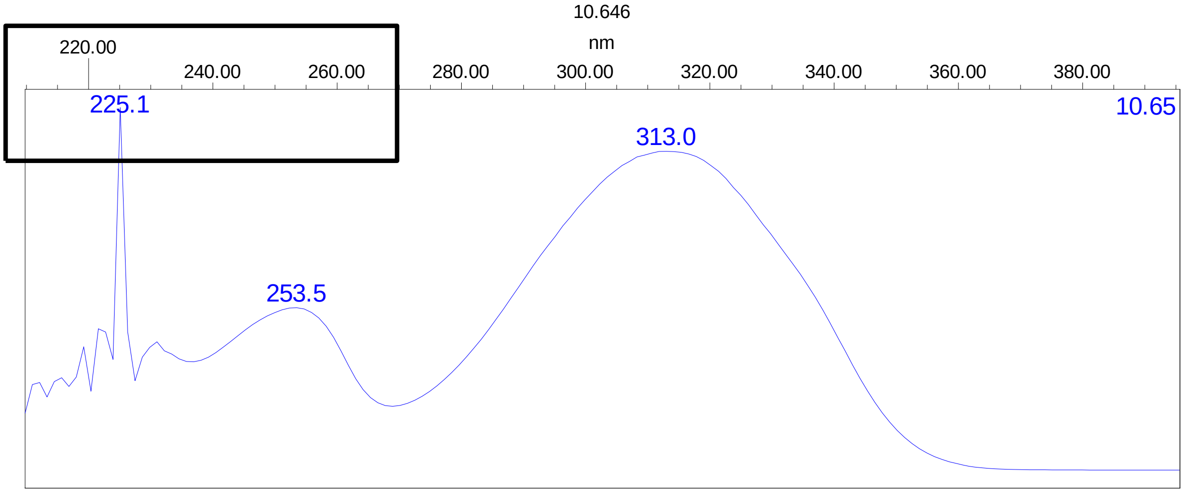

Figure S75. HPLC chromatogram of 3′-hydroxyflavone 4′-*O*-*β*-D-(4″-*O*-methyl)-glucopyranoside (1c)

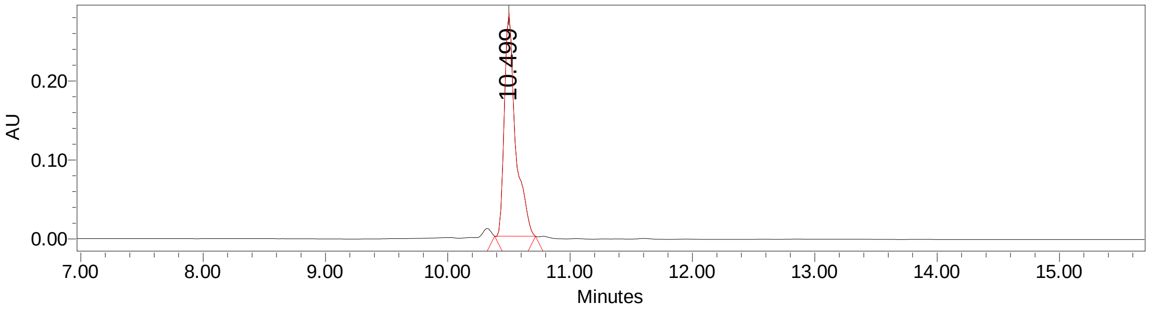

Figure S76. UV maxima of 3′-hydroxyflavone 4′-*O*-*β*-D-(4″-*O*-methyl)-glucopyranoside (1c) obtained after HPLC analysis

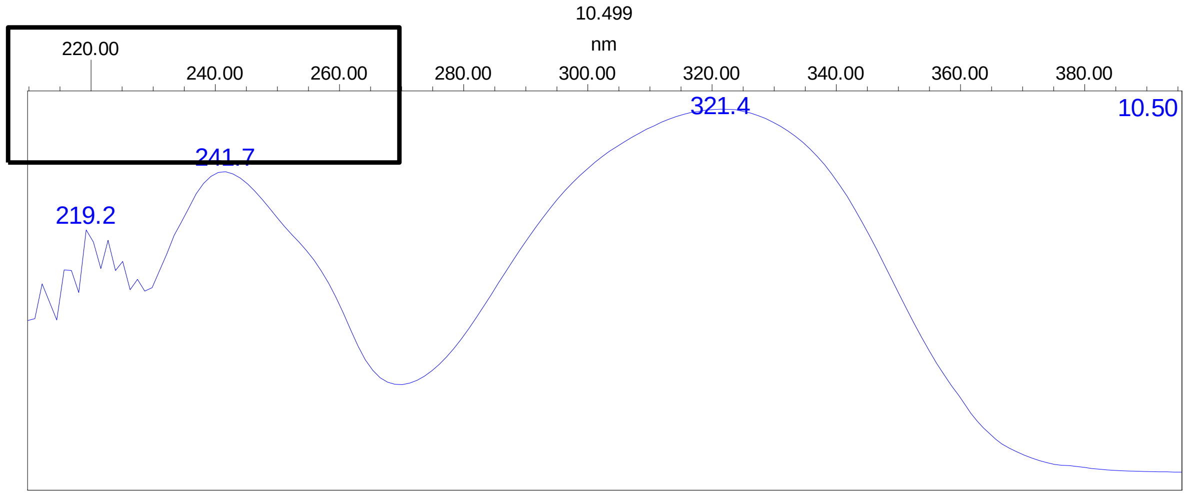

Figure S77. HPLC chromatogram of 5-hydroxyflavone (2)

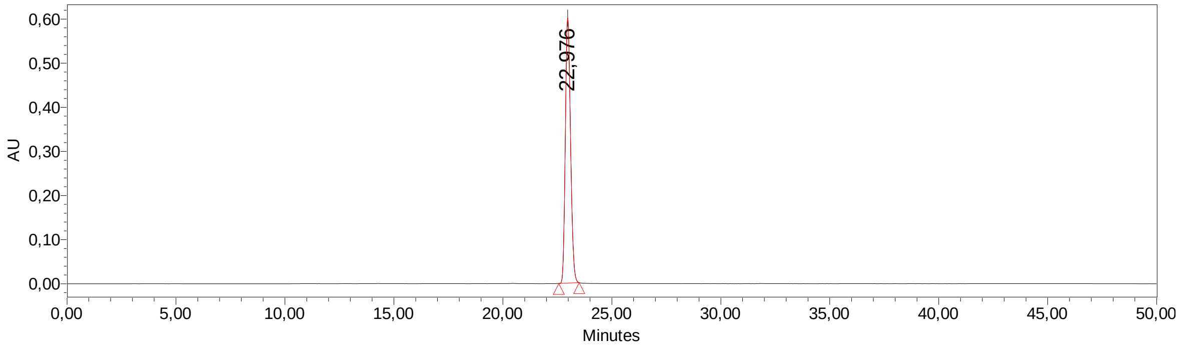

Figure S78. UV maxima of 5-hydroxyflavone (2) obtained after HPLC analysis

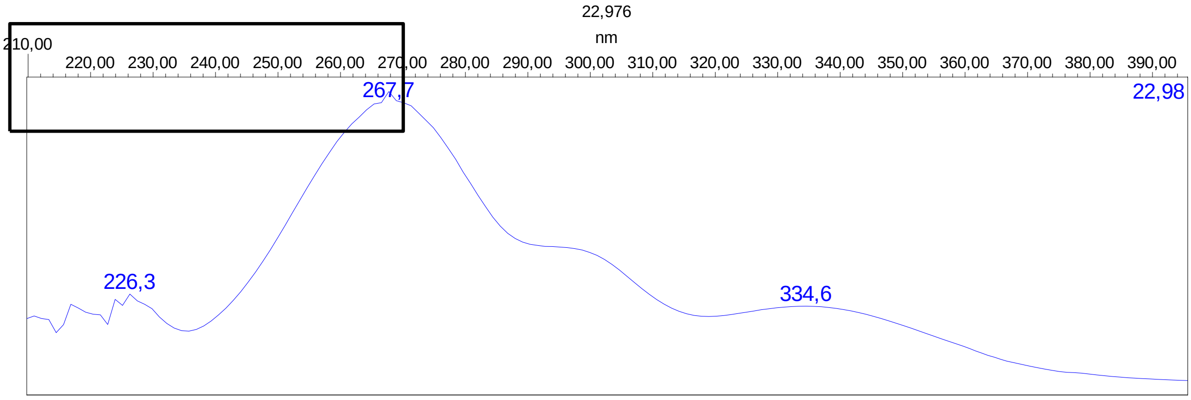

Figure S79. HPLC chromatogram of 5-hydroxyflavone 4′-*O*-*β*-D-(4″-*O*-methyl)-glucopyranoside (2a)

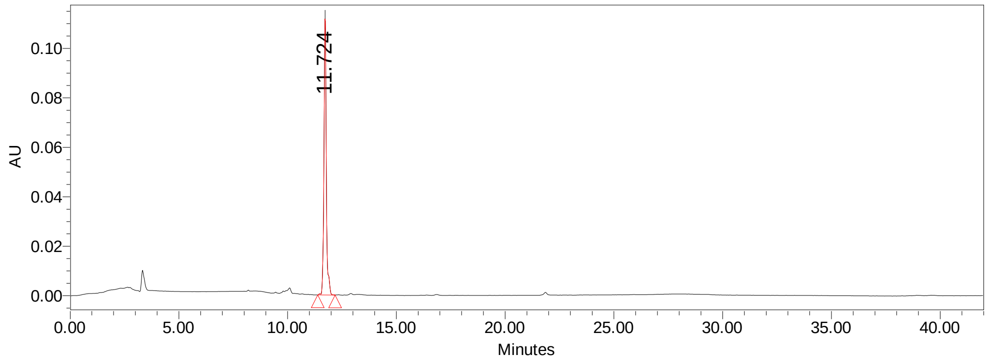

Figure S80. UV maxima of 5-hydroxyflavone 4′-*O*-*β*-D-(4″-*O*-methyl)-glucopyranoside (2a) obtained after HPLC analysis

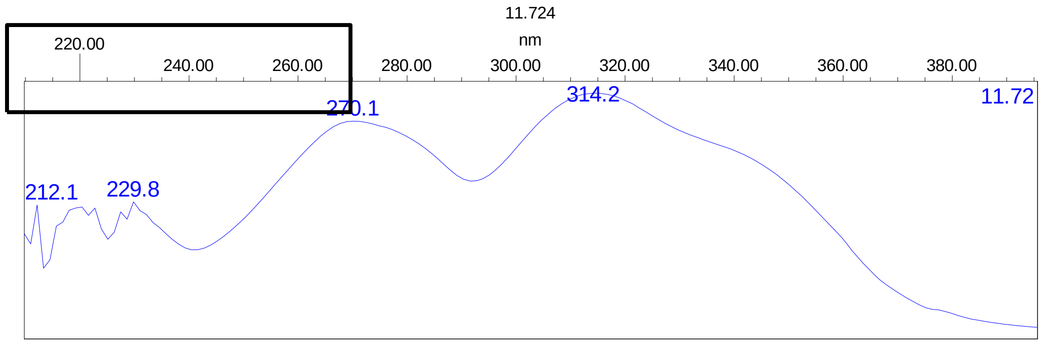

Figure S81. HPLC chromatogram of 6-hydroxyflavone (3)

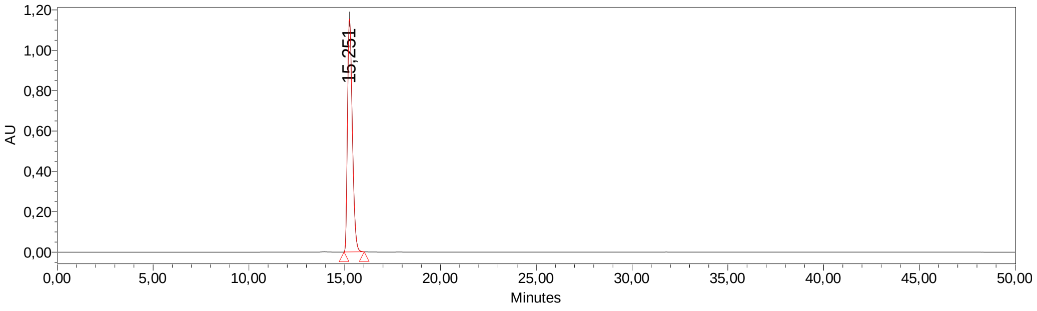

Figure S82. UV maxima of 6-hydroxyflavone (3) obtained after HPLC analysis

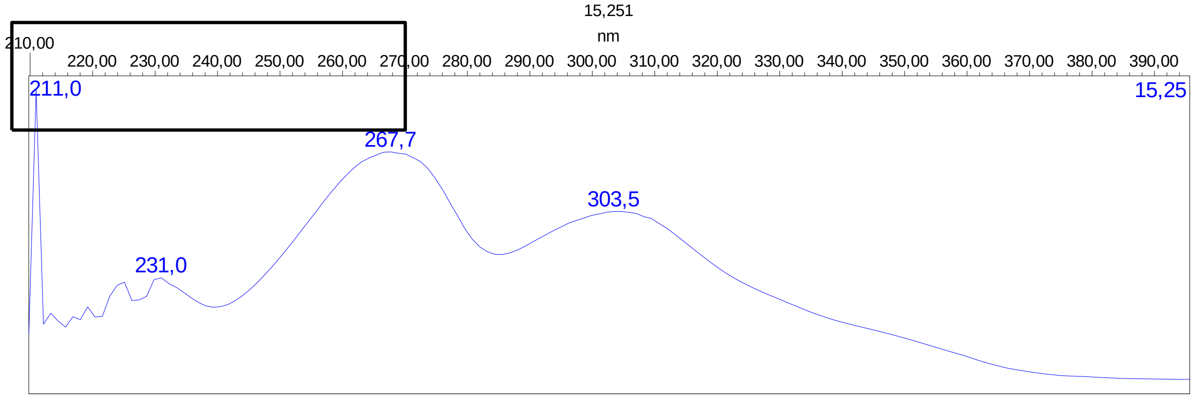

Figure S83. HPLC chromatogram of flavone 6-*O*-*β*-D-(4″-*O*-methyl)-glucopyranoside (3a)

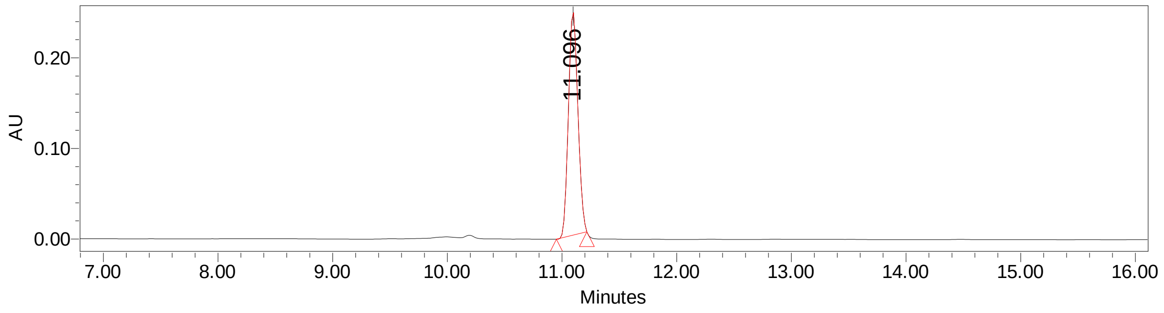

Figure S84. UV maxima of flavone 6-*O*-*β*-D-(4″-*O*-methyl)-glucopyranoside (3a) obtained after HPLC analysis

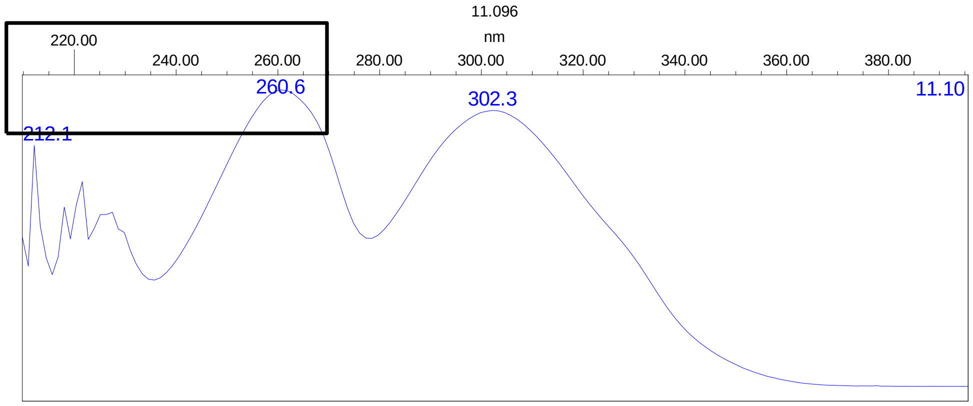

Figure S85. HPLC chromatogram of 7-hydroxyflavone (4)

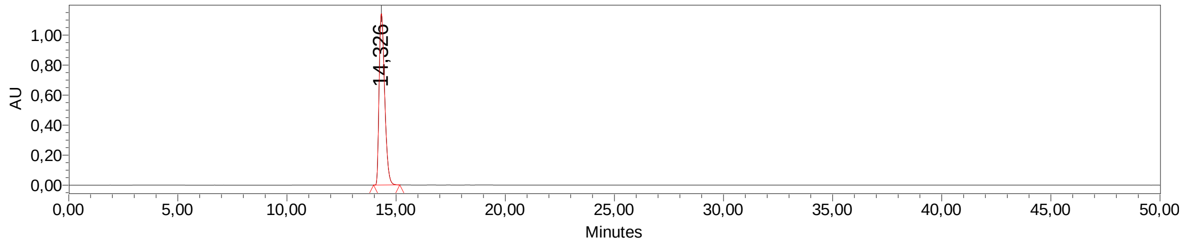

Figure S86. UV maxima of 7-hydroxyflavone (4) obtained after HPLC analysis

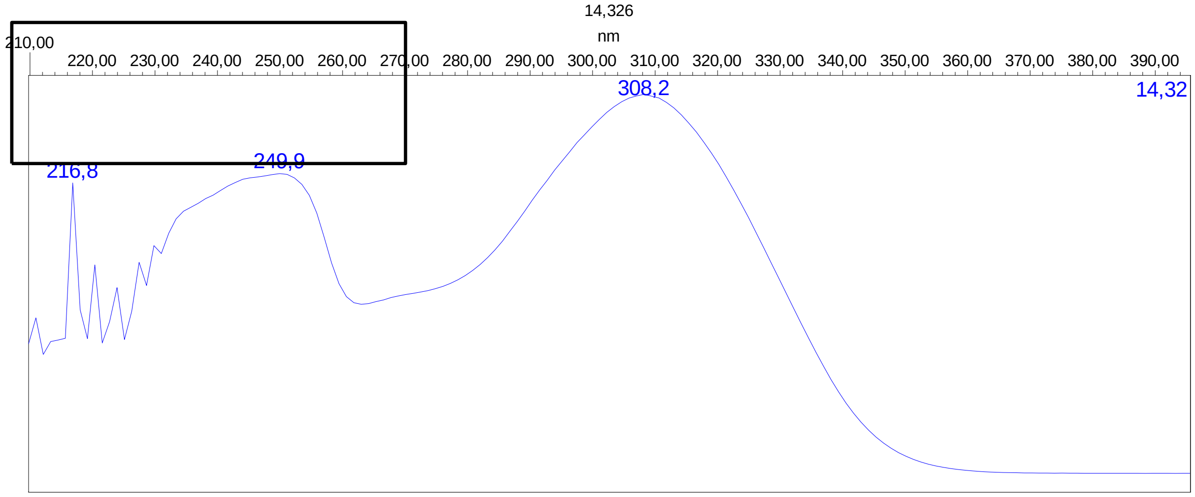

Figure S87. HPLC chromatogram of flavone 7-*O*-*β*-D-(4″-*O*-methyl)-glucopyranoside (4a)

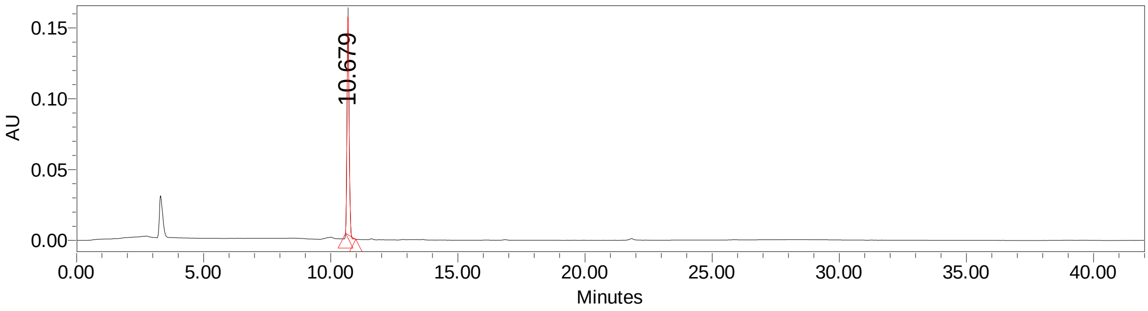

Figure S88. UV maxima of flavone 7-*O*-*β*-D-(4″-*O*-methyl)-glucopyranoside (4a) obtained after HPLC analysis

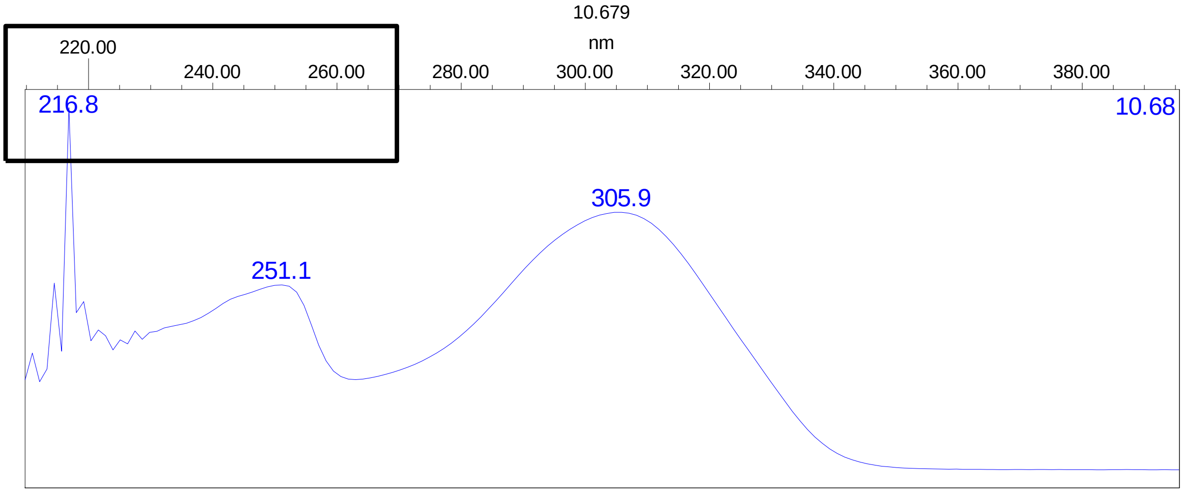

Figure S89. HPLC chromatogram of 4′,7-dihydroxyisoflavone (5)

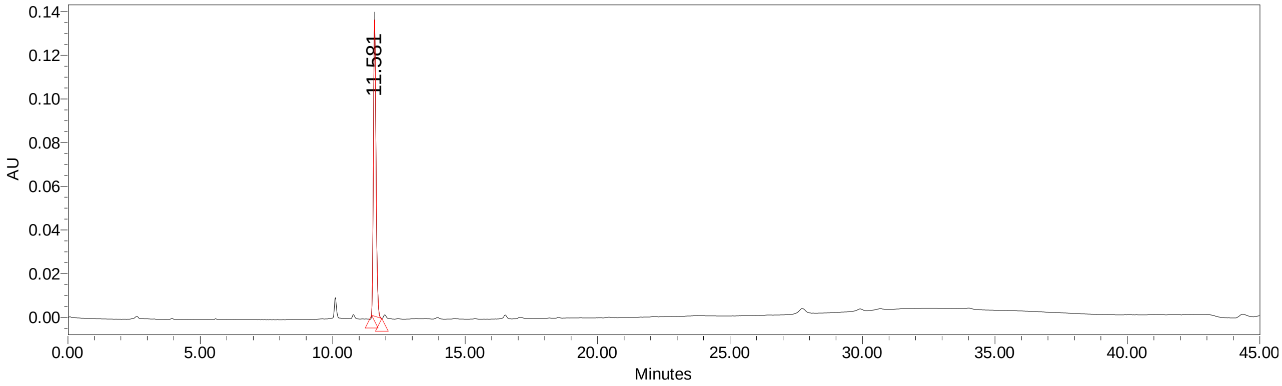

Figure S90. UV maxima of 4′,7-dihydroxyisoflavone (5) obtained after HPLC analysis

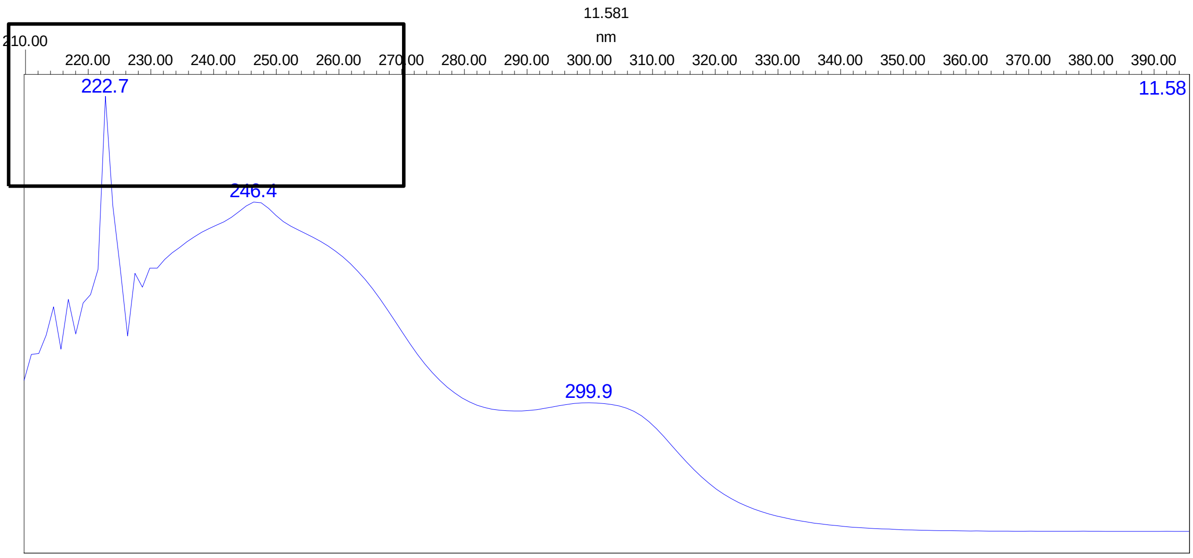

Figure S91. HPLC chromatogram of 4′-hydroxyisoflavone 7-*O*-*β*-D-(4″-*O*-methyl)-glucopyranoside (5a)

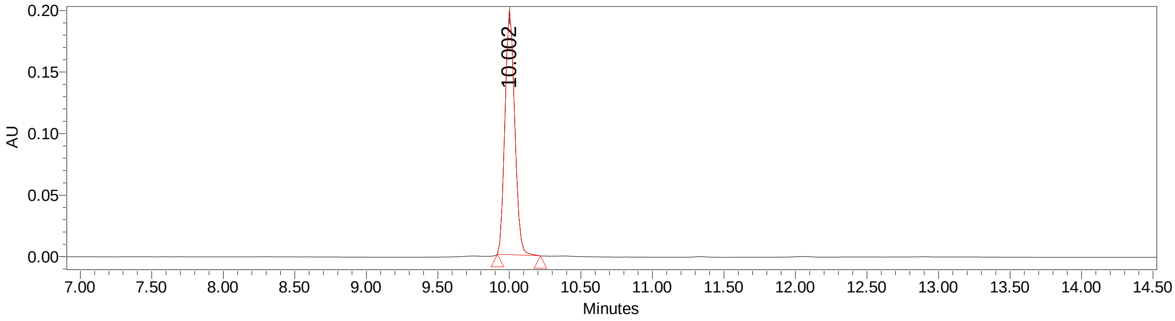

Figure S92. UV maxima of 4′-hydroxyisoflavone 7-*O*-*β*-D-(4″-*O*-methyl)-glucopyranoside (5a) obtained after HPLC analysis

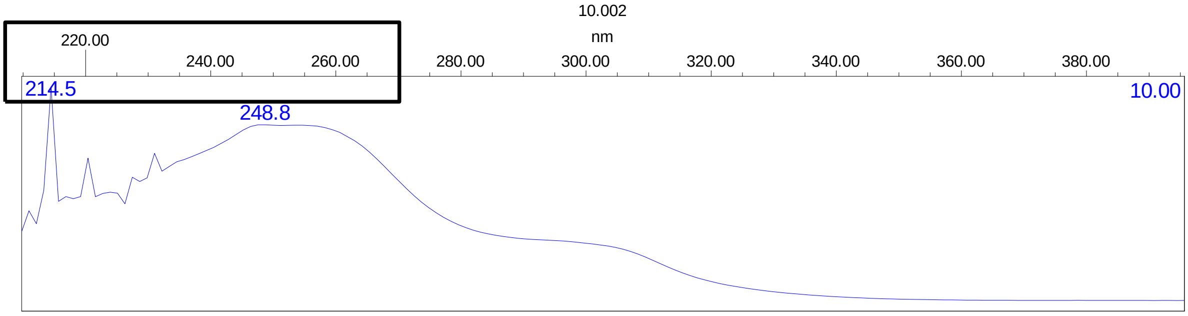

Figure S93. HPLC chromatogram of 7-aminoflavone (6)

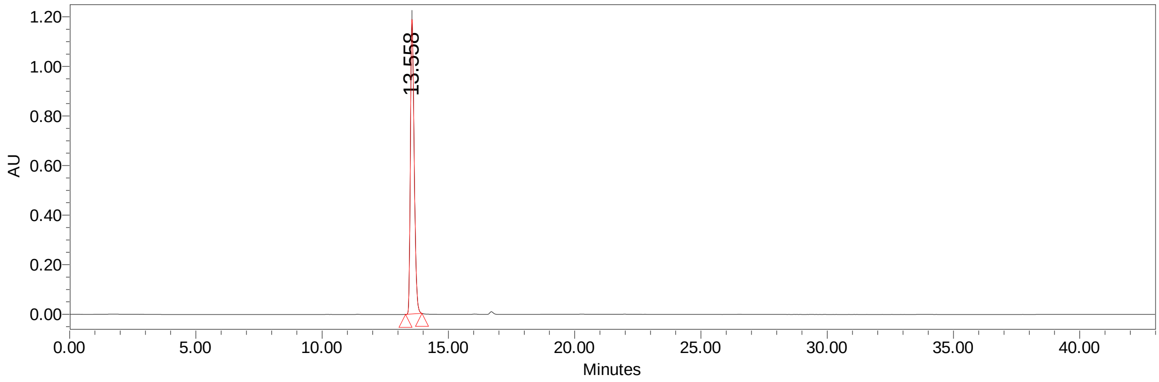

Figure S94. UV maxima of 7-aminoflavone (6) obtained after HPLC analysis

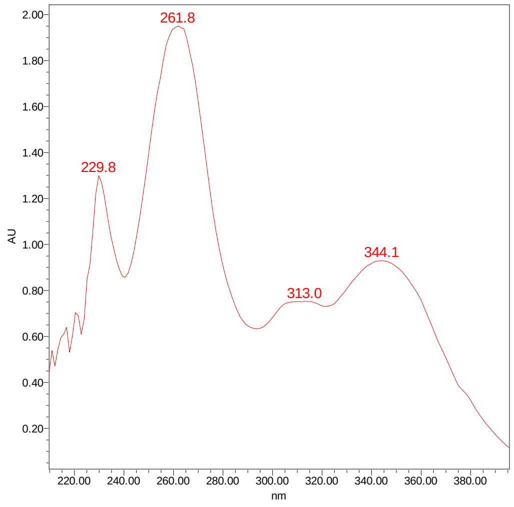

Figure S95. HPLC chromatogram of 7-acetamidoflavone (6a)

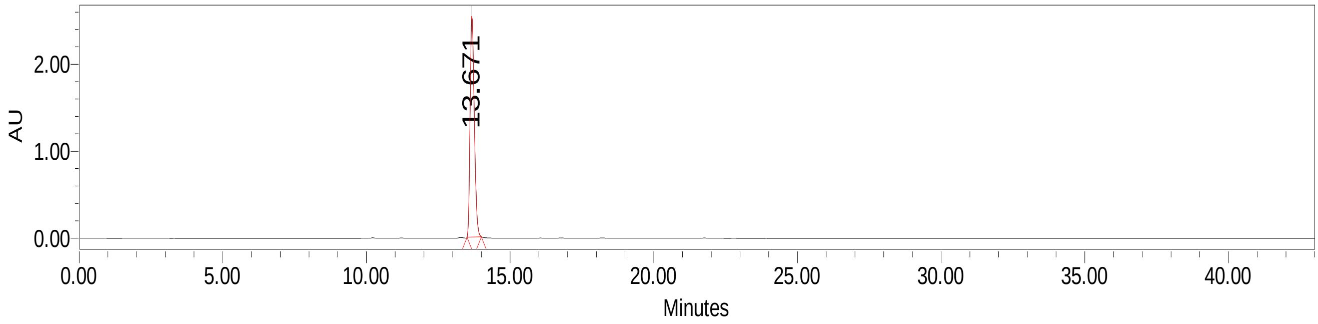

Figure S96. UV maxima of 7-acetamidoflavone (6a) obtained after HPLC analysis

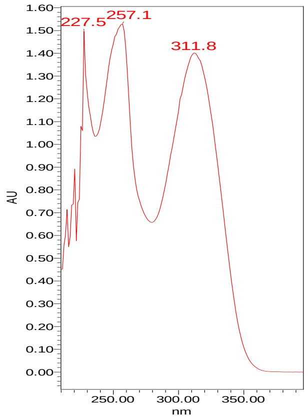

Figure S97. HPLC chromatogram of 4′-hydroxy-7-acetamidoflavone (6b)

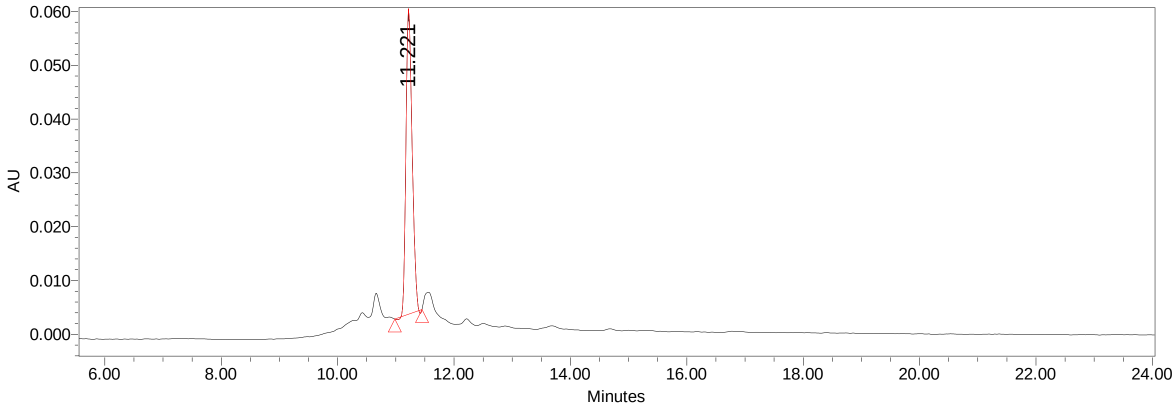

Figure S98. UV maxima of 4′-hydroxy-7-acetamidoflavone (6b) obtained after HPLC analysis

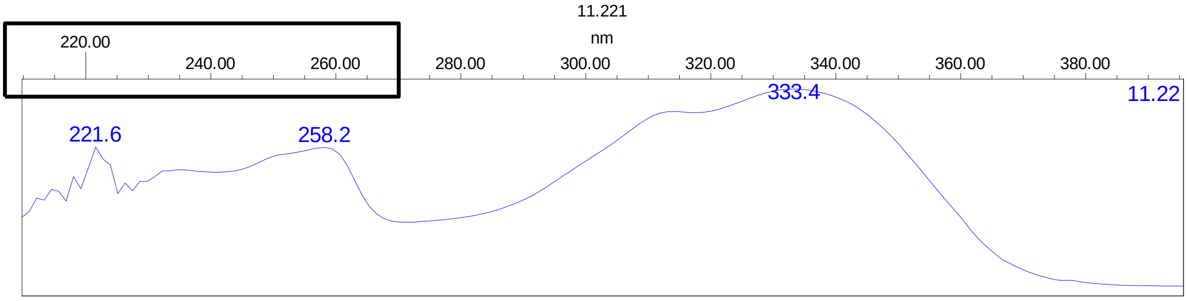

Supplement: Supplementary file 1 [file molecules-23-01356-s001.pdf]
